# Development and validation of material selection method for cost-effective lab-scale 3D-printed platforms in biotechnology

**DOI:** 10.3389/fbioe.2026.1850515

**Published:** 2026-08-31

**Authors:** Ayur Jitendra Ghalot, Alexander Gießel, Prathamesh Thakare, Melika Asvadi, T. A. Stefanie Nguyen, Thomas Walther, Nandor Ziebart

**Affiliations:** Chair of Bioprocess Engineering, Institute of Natural Materials Technology, TUD Dresden University of Technology, Dresden, Saxony, Germany

**Keywords:** 3D-printing, biocompatibility testing, biotechnology, mechanical properties, microbial cultivation, photopolymer resins, PLA filaments

## Abstract

Additive manufacturing is increasingly explored in microbial biotechnology, offering cost-effective, customizable, and scalable solutions. However, the lack of standardized methods for evaluating 3D-printed materials in microbial cultivation applications limits their broader adoption. This study proposes a reproducible framework to assess 3D-printed materials, integrating printability, autoclavability, mechanical testing (Shore D hardness and 3-point bending), and microbial biocompatibility testing. Five polylactic acid (PLA) filaments and twelve photopolymer resins were evaluated. Two polylactic acid filaments were identified as suitable for single-use applications, while a biocompatible photopolymer resin demonstrated potential for reusable components. Autoclaving emerged as the most critical step, eliminating half of the tested materials due to cracking or degradation. Notably, cost-efficient materials (e.g., eSun Standard Peach Pink and Elegoo plant-based Tough Grey resin) performed comparably to high-priced alternatives within the scope of microbial biotechnology. The method’s applicability was validated by 3D-printing shake flasks using Fused Deposition Modelling and Stereolithography, which achieved microbial cultivation outcomes with *Escherichia coli* comparable to glass flasks. This work underscores the importance of robust material selection criteria and provides a systematic framework for advancing additive manufacturing in microbial biotechnology.

## 1 Introduction

Additive manufacturing is increasingly pivotal in the development of flexible, rapid, and cost-efficient solutions for experimental and industrial biotechnology (Priyadarshini et al., 2024; Raddatz et al., 2017). The expanding biotechnology market demands workflows and production platforms that are not only affordable but also quickly adaptable to changing research requirements and experimental parameters. While established stainless steel and glass bioreactors offer reliability, they often lack the versatility needed for rapid iteration, on-demand custom geometries, or integration of novel functionalities that modern research and small-batch production require.

Additive manufacturing addresses these challenges by enabling the custom fabrication of bioreactor components and entire lab-scale reactors, disposable labware, and tailored experimental setups directly from CAD (Computer-Aided Design) models on demand (Raddatz et al., 2017; Satzer and Achleitner, 2022). This approach allows for the creation of complex geometries and experimental devices that cannot be manufactured using conventional methods, supporting laboratory applications. The rapid development and widespread availability of consumer-grade 3D-printers have expanded the accessible material palette, increased the variety of achievable mechanical and functional properties, and lowered the cost and lead time of device development (Satzer and Achleitner, 2022; Quan et al., 2020; Sandanamsamy et al., 2023). Importantly, additive manufacturing enables rapid, adaptable prototyping with minimal material waste, facilitating comprehensive evaluation of mechanical properties and biocompatibility under standardized conditions (Nelson et al., 2024; Kumar et al., 2023).

FDM (Fused Deposition Modelling) and SLA (Stereolithography) printing are the two most widely used additive manufacturing approaches for producing low-cost laboratory components (Raddatz et al., 2017; Satzer and Achleitner, 2022; Sandanamsamy et al., 2023; Husna et al., 2024; Lücking et al., 2015). In FDM, filaments of thermoplastic polymers such as PLA (polylactic acid) are melted and extruded layer by layer through a nozzle, whereas SLA relies on the photopolymerization of liquid resins using defined irradiation wavelengths. Due to these fundamentally different printing mechanisms, both methods exhibit characteristic process-dependent limitations (Sandanamsamy et al., 2023; Husna et al., 2024). For FDM printing, parameters such as nozzle temperature, bed temperature, and printing speed control the thermal history and porosity of the printed part, which in turn influence both the amount and composition of leachable additives and degradation products, as well as overall print quality (Sandanamsamy et al., 2023; Kumar et al., 2023; Rindelaub et al., 2019; Zennaki et al., 2022). In SLA printing, reproducibility is affected by variations in printing parameters (exposure times, print orientation and layer height) as well as post-processing steps, particularly the uniformity of post-curing light exposure and the efficiency of solvent washing, which lead to inconsistencies in polymer crosslinking and monomer content (Brooks and Yadavalli, 2025; Oh et al., 2023; Mohammed and Mohammed, 2024; Bagheri and Jin, 2019). For SLA-printed parts specifically, mitigating leachate-related effects requires defined post-curing protocols, including UV-curing of surfaces and thermal post-curing at elevated temperatures (Husna et al., 2024; Bagheri and Jin, 2019; Mahmud et al., 2025; Katheng et al., 2021). Therefore, the characteristic parameters for both types of printing must be iterated in a first step to ensure satisfying printing results with validation for mechanical properties of the printed parts (Oh et al., 2023; Mohammed and Mohammed, 2024; Mahmud et al., 2025). Beyond basic printability and mechanical testing, evaluation frameworks must systematically address factors such as the impact of over-curing (especially brittle failure in SLA parts) and identification of design flaws (Quan et al., 2020; Mahmud et al., 2025; Katheng et al., 2021).

The potential release of uncured polymer precursors and volatile organic compounds (VOCs) from printed materials under biological exposure conditions influences the applicability of printed parts for biological applications (Achleitner et al., 2024; Shalem et al., 2024; Licata et al., 2024; Pérez-Davila et al., 2022). The harmful effects can be evaluated through biocompatibility testing (Rindelaub et al., 2019; Licata et al., 2024; Oskui et al., 2016). To assess material stability for biotechnology applications, autoclaving compatibility must be verified to ensure components can withstand standard sterilization cycles (Pérez-Davila et al., 2022; Kronenberger et al., 2025). 3D-printed materials for biotechnology applications require standardized biocompatibility validation analogous to ISO 10993 protocols (ISO 10993 - Part 1, DIN German Institute for Standardization, 2024; ISO 10993 - Part 5, DIN German Institute for Standardization, 2009; ISO 10993 Part 12, DIN German Institute for Standardization, 2021). Although previous studies demonstrate that PLA filaments and several photopolymer formulations can exhibit biocompatibility when appropriate sterilization procedures are applied (Achleitner et al., 2024; Pérez-Davila et al., 2022; Neijhoft et al., 2023). However, their actual performance remains sensitive to process variables, batch-to-batch differences, and environmental conditions.

Commercial PLA filaments and photopolymer resins may exhibit batch-to-batch inconsistencies in molecular weight, crystallinity and composition of additives. These formulation differences can substantially affect mechanical properties post-autoclaving and biological compatibility with microbial cultures but also the dimensional accuracy of the printed components (Nelson et al., 2024; Brooks and Yadavalli, 2025; Schittecatte et al., 2023; Klimczuk et al., 2024). Batch variability and cytotoxic leachates even in resins certified as biocompatible under set conditions and printing parameters which necessitate thorough testing for reliable biological use and make it difficult to compare 3D-printed materials for application-relevant use (Brooks and Yadavalli, 2025; Oskui et al., 2016; Guttridge et al., 2022). Since manufacturer datasheets do not typically include lot-specific stability data on molecular weight, crystallinity, additive composition, or leachate profile, batch consistency cannot be ensured from datasheets alone and must be verified experimentally. Consequently, inherent dimensional inaccuracies and workflow-dependent due to batch variations in both FDM- and SLA-printing frequently require iterative design–print–test cycles to ensure that printed components meet functional requirements such as tight dimensional tolerances, fluid pathways, and mechanical interfaces needed in biotechnology equipment (Katheng et al., 2021; Solouki et al., 2024; Gordeev et al., 2018; Panico et al., 2025). These combined challenges necessitate rigorous validation combined with mechanical testing to ensure dimensional accuracy and long-term durability of printed components.

Therefore, a standardized step-by-step method is essential to systematically validate low-cost, commercially available PLA filaments and photopolymer resins under controlled parameters, ensuring safe, consistent, accessible, and biocompatible materials for biotechnological applications. Given the large variety of commercially available filaments and photopolymers, a structured approach is essential to determine which materials remain mechanically stable and are compatible with microbial cultivation. Although photopolymer resins are formulated to minimize cytotoxicity and ensure biomedical compatibility, their high cost limits their widespread adoption in routine biotechnological workflows (Satzer and Achleitner, 2022; Guttridge et al., 2022). This research addresses the gap by proposing a method for systematic evaluation 3D-printed materials to use in microbial biotechnology applications through three key approaches: (i) print optimization and printability evaluation to ensure reproducible, defect-free prints across smaller (30 mm × 30 mm × 3 mm) and bigger (70 mm × 75 mm × 7 mm) geometry scales (ii) mechanical characterization after each step of manufacturing to assess material stability for autoclaving (iii) biocompatibility testing using microbial cultivation for test samples to examine the effects of potential leachates (Sandanamsamy et al., 2023; Husna et al., 2024; Neijhoft et al., 2023; Rizal et al., 2023; Zwietering et al., 1990).

A systematic evaluation method was developed to identify and validate cost-effective, large batch comprising commercially available five PLA filaments (< € 40 / kg / brand) and twelve photopolymer resins (< € 200 / kg / brand) for microbial biotechnology applications. This process method involved market analysis, community reviews, and successive stages of material screening, including printability testing, autoclavability, mechanical testing, and biocompatibility test aimed at microbial cultivation (Mohammed and Mohammed, 2024; Licata et al., 2024; Mukarramah et al., 2020). Materials that met robust criteria for print quality and consistency were subjected to further testing, while those failing to meet reproducibility were excluded early in the process. Remarkably, the findings reveal that an evaluated batch of low-cost materials priced at approximately €20 per kg can perform competitively with premium formulations costing six times more in a test application with printed shaking flasks. This research also illustrates the practical utility of the developed method and highlights how it can guide cost-efficient material selection for modular and scalable bioprocessing components in microbial biotechnology.

It should be emphasized that the proposed evaluation method is developed and validated to check for autoclavable 3D-printed materials (PLA filaments and photopolymer resins).

## 2 Materials and methods

The core objective of this research is to develop a method to systematically identify cost-effective materials for 3D-printed designs used for microbial cultivations and other bioprocessing applications. Using this method, we curated a set of photopolymer resins and PLA filaments, selected based on commercial availability, industry standards, and community insights. By going through the evaluation steps of the method, a printability assessment, an autoclaving test and a mechanical performance test followed by biocompatibility testing. In order to validate the materials selected through the process, widely used shake flasks were 3D-printed for application check.

### 2.1 Material selection

The Creality K1 Max (Creality 3D Technology Co., Ltd., Shenzhen, PR China) was used for FDM printing. For SLA printing, the Prusa SL1S Speed and Prusa CW1S post-processing unit (both Prusa Research s.r.o., Prague, Czech Republic) were employed. Slicing for both platforms was performed using Prusa Slicer version 2.9 (Prusa Research s.r.o., Prague, Czech Republic).

PLA was chosen because it is easy to process by fused deposition modelling, inexpensive, and already widely used in biomedical and biotechnology applications, making it a practical and well-studied material for accessible, reproducible designs (Satzer and Achleitner, 2022; Sandanamsamy et al., 2023). PLA is generally regarded as non-toxic and biodegradable, but commercial PLA filaments contain pigments and other additives, so each specific formulation still requires validation for safe use in microbial cultivation and biocompatible applications21. Standard PLA, alongside enhanced variants PLA+ and PLA HP, which offer superior mechanical properties via additives, were tested to evaluate improvements in mechanical performance and microbial cultivation. The PLA filaments selected for this research are Creality PLA HP Black (Creality, Shenzhen, PR China), Elegoo PLA+ Grey (Elegoo, Shenzhen, China), eSun PLA + White (Shenzhen Esun Industrial Co., Ltd., Shenzhen, China), colorFabb PLA HP White (colorFabb B.V., Belfeld, Netherlands), and Verbatim PLA Blue (Verbatim Americas LLC, Charlotte, United States). All filaments were stored in a closed container with silica gel pellets. One standard PLA served as the FDM reference material due to its standard availability and baseline properties, while PLA+ and PLA HP variants were tested to evaluate improvements in mechanical performance and microbial cultivation as suggested by Satzer and Achleitner (2022).

SLA was selected as another printing technology utilizing the photopolymerization of suitable resins. SLA enables high-resolution (as low as 25 µm) printing with better surface quality and precision than FDM printing (0.2 mm layer height and 0.4 mm nozzle), making it suitable for producing intricate parts that tight tolerances (Quan et al., 2020; Husna et al., 2024). The photopolymer resins selected for this study are SirayaTech Blu Clear V2, SirayaTech Build Sonic Grey and SirayaTech Craft Ultra Clear (all three SirayaTech, CA, United States), Phrozen Protowhite Rigid, Phrozen Aqua Snow Grey and Phrozen Aqua Hyperfine Graphite (all three Phrozen Technology Inc., Kaohsiung, Taiwan (R.O.C.)), Liqcreate Bio Med Clear, Liqcreate Premium Tough and Liqcreate Tough X (all three Liqcreate BV, Eindhoven, Netherlands), Prusament Tough Orange (Prusa Research s.r.o., Prague, Czech Republic), eSun Standard Peach Pink (Shenzhen eSun Industrial Co., Ltd., Shenzhen, PR China), and Elegoo plant based Tough Grey (Elegoo, Shenzhen, PR China). All resins were used after thorough shaking and without any modifications and were stored in the dark when not used. One standard photopolymer resin served as the SLA reference material due to its availability and baseline properties with other variants to validate the test method.

### 2.2 Laboratory equipment

Sterilization of 3D-printed parts were performed using a Laboklav 25 Autoclave (SHP Steriltechnik AG, Detzel, Germany). Spectrophotometric measurements, e.g., determining the optical density (OD600) at 600 nm of cultivation experiments were conducted using a Thermo Scientific Genesys 150 UV-Vis Spectrophotometer (Thermo Fisher Scientific, Waltham, United States). Further OD600 measurements were validated with an in-house developed measurement system (SeCaT-meter 600) as part of one shake flask experiment (Michl et al., 2026). Cultivation experiments were conducted in Minitron and Multitron Large Capacity Incubator Shakers (both Infors HT, Einsbach, Switzerland), and in an Incubator SalvisLab AG6015 (SalvisLab, Baar, Switzerland). All printing and post-processing steps were completed before final dimensional measurements using a vernier caliper (Brüder Mannesmann Werkzeuge GmbH, Remscheid, Germany).

### 2.3 Process flow for FDM and SLA printing

The first step of material validation starts with determining print parameters for each material with an iterative approach. This process ensured that each 3D-printed material was evaluated for printability, printing consistency, and optimization of both printing with post-processing parameters. After selecting a resin or filament brand, initial print settings were determined using manufacturer recommendations or, where unavailable, by drawing on community-shared experiences, utilizing the Maker Trainer community (Maker Trainer, 2022). Temperature towers and boat-shaped test objects were printed for FDM to calibrate nozzle temperature, heat bed temperature and printing speed. For SLA, the Prusa calibration object and a lattice test cube were used to optimize exposure times and layer heights and to evaluate the influence of print orientation. Iterative adjustments to printing parameters were made following any print failures until consistent and reliable printability was achieved across all calibration objects. FDM print failures included poor first-layer adhesion where the print does not stick to the heated bed causing detachment; stringing, which leaves thin plastic threads between parts of the model; and blobs or zits. These failures results due to inconsistent material flow due to incorrect retraction settings, over-extrusion, excessive nozzle temperature, or presence of elevated material moisture content (Sandanamsamy et al., 2023). SLA print failures involve first-layer adhesion problems to the build plate, insufficient exposure time causing poor layer bonding, and over-curing or prolonged solvent exposure during post-processing that leads to brittleness, compromised fine detail accuracy, and reduced mechanical integrity. For SLA-printed parts post-processing steps including washing in isopropyl alcohol (IPA), drying, and UV-curing critically influence final surface quality, mechanical properties, and dimensional stability. More details of FDM and SLA printing adaptations can be found in the Supplementary Materials S1, S2, respectively. All printing and post-processing steps were completed before final measurements using a standard vernier caliper (±0.02 mm accuracy), with additional iterations on exposure time, layer height, wash and curing duration performed as needed to ensure dimensional accuracy (Brooks and Yadavalli, 2025; Katheng et al., 2021). The reliability of a print was defined by achieving reproducible dimensional accuracy and surface quality in the benchmark tests. The post-printing processes, particularly for SLA, were iteratively adjusted starting from manufacturer’s recommendations. A single standardized protocol (washing of 10 min in total, 10 min of drying, and 20 min UV-curing time in total) was then selected and applied to all subsequent prints, representing a compromise across the tested materials rather than a material-specific optimization. A curing-time sensitivity analysis was subsequently performed on three representative resins with different curing times with mechanical tests suggesting that the 20-min endpoint represents the balanced operating point across biocompatibility, Shore D hardness, and 3-point bending test with HPLC analysis (see Supplementary Materials S16, S17). Moreover, the protocol was also chosen based on visual inspection for surface defects, preservation of fine features, and absence of cracks or warping. These criteria provide readers with clear guidance to establish suitable conditions for their own setups. Throughout, the Maker Trainer community served as a valuable starting point for troubleshooting and refining settings, particularly in the absence of explicit manufacturer guidance (Maker Trainer, 2022). This method ensured that all materials were processed under conditions facilitating reliable fabrication and reproducibility (Brooks and Yadavalli, 2025; Oh et al., 2023; Maker Trainer, 2022).

Upon establishing reliable printability, each material was validated using lattice cubes (20 mm × 20 mm × 20 mm), followed by a custom rectangular test model (68 mm × 75 mm × 3 mm) to assess parameter transferability to larger surface area parts (provided in Supplementary Materials S3, S4). This evaluation demonstrated that the printing parameters optimized using standard calibration objects do not necessarily transfer directly to parts with larger surface areas (especially with SLA), which frequently require adjusted settings in both printing techniques to achieve comparable print quality (Quan et al., 2020; Husna et al., 2024; Katheng et al., 2021).

As the mechanical and sterilization response of 3D-printed parts is strongly geometry-dependent, three distinct standardized specimen geometries were used throughout this study, each matched to its intended test purpose: (i) a rectangular plate (52.5 mm × 70 mm × 7 mm) for Shore D hardness testing; (ii) a rectangular beam (80 mm × 10 mm × 4 mm, span 50 mm) for 3-point bending testing and (iii) a disc-shaped specimen (Ø 27 × 2 mm) for steam sterilization and leachate biocompatibility testing. All specimens were printed with 100% infill (rectilinear pattern for FDM) and with print orientation held constant across replicates so that the applied load in the bending test was oriented perpendicular to the layer lines, accounting for anisotropy caused by weaker interlayer adhesion. The STL files for all specimen geometries are provided in the Supplementary Materials S6–S8.

### 2.4 Steam sterilization test

Steam sterilization test was carried out in accordance with ISO 10993-12 (ISO 10993 Part 12, DIN German Institute for Standardization, 2021) to ensure reliability. The autoclave cycle was performed at 121 °C for 20 min, in accordance with commonly accepted sterilization protocols (Kronenberger et al., 2025; Neijhoft et al., 2023). The sterilization test serves a dual purpose, evaluating mechanical stability of printed materials after autoclaving and sterility during biocompatibility testing (Pérez-Davila et al., 2022; Kronenberger et al., 2025). For each 3D-printed material, a total of nine standardized disc-shaped (Ø 27 × 2 mm) test samples were printed (see Section 2.7.1, calculations found in Supplementary Material S7).

### 2.5 Shore D hardness test

Shore D hardness testing was employed as a rapid, cost-effective approach to assess the mechanical properties of 3D-printed samples. The testing utilized a Sauter Durometer HDD 100 1, mounted on a manual shore test stand TI-DL (both Sauter GmbH, Balingen, Germany), with a calibrated constant load of 50 N for all measurements. The precision of the durometer is ±1 D of maximum value, with a resolution of 0.1 D. Hardness comparison plates AHBD-01 (KERN and SOHN GmbH, Balingen, Germany) were employed for reference to verify durometer calibration, prior to each measurement. Test samples for hardness (found in Supplementary Material S7) were rectangular in shape (52.5 mm × 70 mm × 7 mm), meeting the ISO 868: 2003 minimum thickness of 4 mm (ISO 868: DIN German Institute for Standardization, 2003). This method provides a standardized, quantitative assessment of material surface stability and process consistency following autoclaving, where consistent hardness values across multiple specimens indicate uniform printing and effective post printing process (Oh et al., 2023; Mahmud et al., 2025; Hsueh et al., 2021). Shore D hardness was measured at eight distinct points per sample, spaced at least 6 mm apart (ISO 868: 2003 DIN German Institute for Standardization, 2003), and the average of maximum values were reported, since the durometer has a built-in function to record the maximum hardness value during each measurement. Hardness test was conducted on PLA samples (100% infill) both after printing and following autoclave sterilization. The photopolymer resins samples were tested sequentially after the second IPA wash, 10 min of drying, 10 min of curing the top side, 10 min of curing the lower side, after thermal curing at 80 °C for 120 min (Mahmud et al., 2025), and finally after autoclaving. Thermal post-curing (annealing) was applied specifically for Shore D hardness characterization (80 °C, 30 min), as it maximizes crosslinking density and surface hardness beyond standard UV post-processing (Bagheri and Jin, 2019; Kronenberger et al., 2025). Eight Shore D values for hardness for each material were taken from each material to conduct three independent tests. Material stability was assessed by quantifying the percentage change in hardness values before and after autoclaving, validating both photopolymers curing efficiency and resistance to sterilization of all samples.

### 2.6 3-Point bending test

To determine the flexural mechanical properties of both PLA and photopolymer resin samples, 3-point bending test was performed using an Instron 5564 universal testing machine (Instron, Norwood, United States) equipped with a 1 kN load cell. As part of the mechanical characterization of 3D printed test samples, the measurement of applied force has an accuracy of ±0.4%–0.5% of recorded reading with a resolution of 0.01 N. The distance of deflection is measured with an accuracy of ±0.01 mm and a resolution ±0.015 mm. Test specimens were 3D-printed as rectangular beams with nominal dimensions of 80 mm length, 10 mm width, and 4 mm thickness, following ISO 178:2019 standards for plastics testing (ISO 178: 2019 DIN German Institute for Standardization, 2019). The STL file for the bending test specimen is available in the Supplementary Material S8. For the FDM printed samples an infill of 100% was used with rectilinear infill pattern.

For the bending test, each specimen was supported horizontally on two anvils spaced 50 mm apart. A rounded loading nose with a radius of 5 mm was positioned centrally above the specimen, manually aligned between the supports. Loading was applied at a rate of 1 mm/min following a 10 N preload (with zero reset), then continued at 10 mm/min until reaching a maximum displacement of 15 mm at the beam centre or specimen fracture, whichever occurred first. Load (N) and deflection at maximum load (mm) were recorded throughout the test. The force applied during the test was oriented perpendicular to the layer lines of the FDM and SLA-printed samples, accounting for anisotropy caused by weaker adhesion between layers.

Flexural strength (σf) was calculated according to ISO 178 using Equation 1 (ISO 178: 2019 DIN German Institute for Standardization, 2019):

$$\sigma_{f}=\frac{3FL}{2bh^{2}}$$ (1)

Where F is the maximum load at fracture or maximum bending (N), L is the support span length (50 mm), b is the specimen width (10 mm), and h is the specimen thickness (4 mm). Subsequent analysis included plotting flexural strength against deflection and comparing mechanical behaviour before and after autoclaving. Visual observations of deformation modes, i.e., bending, or brittle fracture, were documented for all samples. For the 3-point bending tests, material stability was evaluated through percentage changes in maximum deflection and flexural strength post-autoclaving. For the bending test, five samples per material were tested across three independent sets.

### 2.7 Biocompatibility test of selected 3D-printed materials for microbial cultivation

Biocompatibility is defined as “an ability of a medical device or material to perform with an appropriate host response in a specific application” by the FDA (U.S. Food and Drug Administration, 2023). The developed method refers to ISO 10993 (parts 1, 5 and 12) and aims to adapt biocompatibility testing for industrial biotechnology applications by using microbial cultivation with *E. coli* MG1655 (*E. coli*) (ISO 10993 - Part 1 DIN German Institute for Standardization, n.d.; ISO 10993 - Part 5 DIN German Institute for Standardization, 2009; ISO 10993 Part 12 DIN German Institute for Standardization, 2021). The rationale of this test is to obtain eluates representative of the potential release of uncured polymer precursors and VOCs from 3D-printed materials under biological exposure conditions (Rindelaub et al., 2019; Pérez-Davila et al., 2022; Oskui et al., 2016).

**FIGURE 1:**
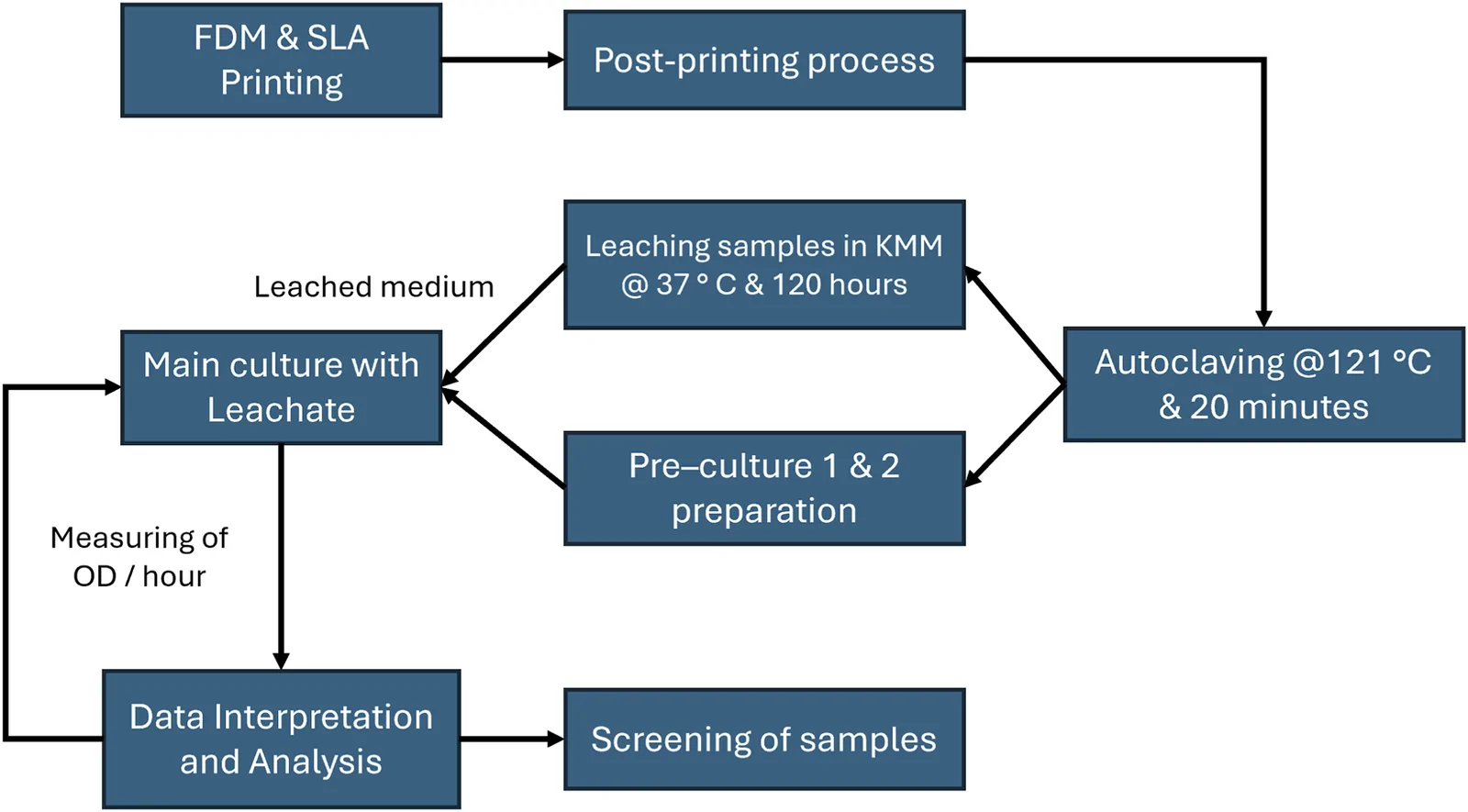
Process flowchart for preparation, biocompatibility testing as leachate test, and evaluation of 3D-printed materials for use in microbial cultivation.

The process flowchart in Figure 1 outlines the experimental method for assessing biocompatibility of 3D-printed materials in microbial cultivation. A comprehensive data analysis procedure was employed to evaluate the growth behaviour of *E. coli* in the presence of the leachates. Data was derived from two independent of triplicate tests conducted on the selected 3D-printed brands, with each sample compared to the corresponding reference samples (negative control) from their respective tests.

#### 2.7.1 Design of test sample for biocompatibility

The test sample design (Ø 27 × 2 mm) used for biocompatibility assessment is identical to that employed for the steam sterilization test detailed in Section 2.4. The test sample was designed in guidance accordance with ISO 10993-12 (ISO 10993 Part 12 DIN German Institute for Standardization, 2021) to ensure reliability. In this study, the extraction ratio (the ratio of surface area of the test sample to the volume of extraction medium), was set at 3 cm^2^/mL. This ratio is critical to achieving accurate assessments of material safety, as it determines both the quantity of leachates evaluated and the biological relevance of test conditions. The test samples were printed to maintain the extraction ratio using 13.1 mL of extraction medium. The calculations of the surface area of the test samples with respect to the extraction ratio and the design of the test sample in accordance to a 100 mL bioreactor which can be found in Supplementary Material S6. The printed samples were stored in an air-conditioned room (∼20 °C) under protection from light exposure.

#### 2.7.2 Test samples for control

For the biocompatibility evaluations conducted adapted from ISO 10993-12, high-density polyethylene (PE-HD, S-Polytech GmbH, Kranenburg, Germany) was selected as the control material for both the positive (+ve) and negative (–ve) references (ISO 10993 Part 12 DIN German Institute for Standardization, 2021). The disc control samples were prepared with the same dimensions as the test specimens (Ø 27 × 2 mm) to ensure geometric comparability between test and control. To prevent changes to the polymer during manufacturing the samples were prepared by water jet cutting. The printed samples were stored in an air-conditioned room (∼20 °C) under protection from light exposure.

#### 2.7.3 Medium composition and preparation for microbial cultivation

In our group commonly used *Kluyveromyces marxianus* mineral medium (KMM) was chosen for biocompatibility testing as microbial cultivation media. The nutritionally rich medium Lysogeny Broth (LB) was used as preculture 1. The recipe of KMM, LB medium and preparation of agar plates is given in the Supplementary Materials S9, S10.

#### 2.7.4 Preparation for test samples

All test samples used for biocompatibility evaluation were autoclaved prior to leaching, in accordance with ISO 10993-12 (ISO 10993 Part 12 DIN German Institute for Standardization, 2021). The leachate biocompatibility test was therefore performed exclusively on post-autoclaved samples, since steam sterilisation is the prerequisite entry point of the biocompatibility workflow defined in Figure 1. The test samples were placed separately into sterile 100 mL borosilicate glass flasks. For the positive control (+ve), three PE-HD samples were immersed in 1.4% w/v phenol solution (3.1 mL) with 10 mL mineral medium during the leaching period, generating a known cytotoxic response baseline. A blind test was omitted. Leaching (elution) was conducted under sterile conditions by sealing all borosilicate glass flasks to prevent contamination and incubating them at 37 °C to simulate microbial cultivation conditions. The leaching period spanned 140 ± 2 h. These leachates were then subjected to cultivation medium with *E. coli* to determine whether the extracted substances exhibited inhibitory or cytotoxic effects compared with pre-defined positive and negative controls.

#### 2.7.5 Revival and recultivation of *Escherichia coli* on agar plates

For subsequent experiments, the wild-type *E. coli* MG1655 strain was revived from a glycerol stock by aseptically streaking cells onto pre-prepared sterile LB agar plates. *Escherichia coli* MG1655 was selected due to its status as the most commonly used laboratory strain with a fully sequenced genome and well-characterized physiology which exhibits robust growth, genetic stability, and minimal mutations, making it a suitable model organism for reproducible and comparative microbiological studies (Wang and Guo, 2024). The plates were incubated inverted at 37 °C for 24 h to allow for colony development. Following incubation, isolated colonies were available for use as inoculum in liquid culture experiments.

#### 2.7.6 Preparation of precultures

A two-step pre-culture was used to prepare *E. coli* MG1655 for reliable inoculation and reduced lag phase in the main cultivation. In the first step (pre-culture 1), a single isolated colony was transferred from an LB agar plate into 3.5 mL of sterile LB medium in a 15 mL centrifuge tube and incubated in a shaking incubator for 6 h (37 °C, 220 rpm). This actively growing pre-culture was then used to inoculate a pre-culture two comprising 22.5 mL KMM medium, 2.5 mL glucose solution (200 g/L stock), and 1.2 mL of the pre-culture 1 in a 250 mL sterile Erlenmeyer baffled shake flask. The pre-culture two was incubated for 16 ± 2 h (37 °C, 220 rpm). For OD, measurement of pre-culture 2, a total volume of 1,000 µL was used in an acrylic cuvette (10 mm × 4 mm × 45 mm). To maintain measurement accuracy in every test, a dilution was prepared by mixing 25 µL of pre-culture 2 with 975 µL of deionized water before determining the OD600.

#### 2.7.7 Main culture preparation and measurement of optical density

After the leaching period (140 h), 10 mL of KMM medium leachate was transferred from each flask into separate sterile 100 mL baffled Erlenmeyer shake flasks, which served as the main culture vessels. To achieve a standardized starting cell density, each sterile shake flask was inoculated with pre-culture 2, which served as the inoculum to accelerate microbial growth across all replicates. The methodology of inoculating the main culture with pre-culture two is given in the Supplementary Material S11. The optical density of pre-culture two was measured and the back-calculated OD600 enabled precise adjustment of inoculation volumes.

All main cultures were inoculated with calculated volume of inoculum (Supplementary Material S11) with an OD600 of 0.1 and incubated at 37 °C and 220 rpm in a shaking incubator. Microbial growth was monitored by regularly measuring OD600 at defined intervals. Optical density measurements were recorded hourly for a duration of 9 h post-inoculation and once again at 24 h to generate growth curves and calculate exponential growth rates under each tested condition. The sample volume for each measurement was consistently 800 µL after dilution, measured in an acrylic cuvette. When OD600 values exceeded 0.4, samples were measured again using deionized water for dilution in the increments of 2, 4 and 10.

#### 2.7.8 Data interpretation and analysis

Data visualization and additional statistical analyses, including growth curves and exponential phase plots and other graphs were performed using Microsoft Excel (Microsoft Corporation, Redmond, WA, United States) and Origin 2024b (OriginLab Corporation, Northampton, MA, United States). The concentration of biomass was measured as OD600, and the natural logarithm of OD600 readings was computed to identify the exponential growth phase. Within this phase, at least three consecutive data points from the ln (OD600) vs. time plot were selected for calculating the specific growth rate (μ), assessed for reliability by R^2^ values approaching unity. Only those samples that reached the exponential phase were considered in the subsequent analysis. Samples that did not display a clear exponential growth phase were excluded from further evaluation. To allow for comparison across tests, the specific growth rate of each sample was normalized to its corresponding negative control (−ve) as a reference, producing relative specific growth rates. The average relative specific growth rate and associated standard deviation, as well as relative deviation, were then calculated based on results from the three replicate tests. Consistency across triplicates was rigorously evaluated.

The growth curves were validated and fitted using a modified Gompertz sigmoidal model (example in Supplementary Material S12) in Origin to quantitatively describe the transition between lag, exponential, and stationary growth phases under the experimental conditions (Wang and Guo, 2024). OD600 was monitored as a proxy for cell density, and Gompertz model parameters; initial cell density, maximum cell density (a), time at maximum growth rate (xc), and growth rate (k), were fitted via nonlinear least-squares fitting in Origin. Model parameters were extracted and plotted relative to the negative control test sample to validate Gompertz model fit and confirm growth curves followed the expected sigmoidal pattern (Wang and Guo, 2024).

### 2.8 Application check of 3D-printed shake flasks

The materials passing the biocompatibility test were set to evaluate the practical utility of the 3D-printed shake flasks produced through this research where application-relevant microbial cultivation experiments (Supplementary Material S14). The 100 mL glass shake flask geometry was used as the model basis. Due to stability issues in the first printing tests the wall thickness was increased for the following iterations.

#### 2.8.1 Optical density comparison of *Saccharomyces cerevisiae* in glass and 3D-printed shake flask

A comparative study was done to validate microbial growth performance with one of the PLA-HP filaments (Creality PLA HP Black) printed shake flasks relative to conventional glass shake flasks. *Saccharomyces cerevisiae* (Baker’s yeast) was cultivated in Identical Yeast Extract Peptone Dextrose (YPD) medium in parallel under controlled incubation conditions of 30 °C and 220 rpm, with optical density measurements taken at every hour with an in-house developed SeCaT-meter 600 OD sensor (Michl et al., 2026). At each time point, cultures were vortexed to resuspend cells, then OD600 was measured immediately in triplicates. Measurements were completed within 30 s per sample to minimize settling effects.

#### 2.8.2 Microbial cultivation using 3D-printed 100 mL shake flasks with *Escherichia coli*

To validate the application of shake flasks, *E. coli* MG1655 was cultured in KMM supplemented with pre-culture two inoculum prepared as mentioned in Section 2.7. The main cultures were incubated at 37 °C at 220 rpm. OD600 was measured at every hour throughout cultivation to assess growth kinetics. The shake flasks were printed using polymers selected through prior screening.

## 3 Results

The results of this research are organized to reflect the systematic method developed for evaluating and selecting suitable 3D-printed materials for microbial biotechnology applications based on a comparison of materials across all evaluation criteria. All the datasets of the results can be found in the folder of “Data sets” of the OSF Repository (Ghalot et al., 2025). The analyses quantified bacterial growth responses on different 3D-printed materials.

### 3.1 Systematic method development for material evaluation and selection

**FIGURE 2:**
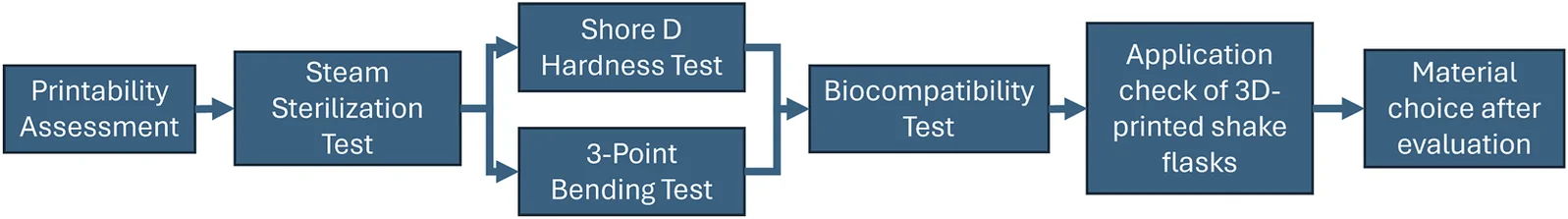
Flow chart of the process method developed for identifying and validating 3D-printed materials for microbial biotechnology applications.

A systematic material selection method was established and implemented to identify 3D-printed materials suited to microbial biotechnology applications as shown in Figure 2. Materials exhibiting consistent printability and robust mechanical performance after autoclaving advanced to standardized biocompatibility testing. Those meeting criteria in all steps were further validated for application.

### 3.2 Material printing and test results

Multiple iterative tests were conducted to optimize print quality by varying printing parameters. For FDM printing, common failure modes were addressed by proper filament storage (prevent moisture absorption), layer height, print speed and nozzle temperature (stringing issues), and heat bed temperature (poor first-layer adhesion). For SLA printing, layer height, print orientation, base exposure times, and initial exposure times were customized depending on part size and geometry. First-layer adhesion failures were addressed by adjusting build plate calibration of the printer and initial exposure time, while insufficient layer bonding was resolved through incremental increases in exposure time. Over-curing of parts during post-printing was mitigated by reducing UV-curing time, while inconsistent washing times in IPA were standardized to preserve mechanical integrity and fine feature accuracy. The optimized printing parameters for all selected brands are provided in Supplementary Material S5.

ColorFabb PLA HP, Liqcreate Tough X, and Liqcreate Premium Tough were excluded from further testing due to unresolvable printing issues (slow speeds, delamination, poor layer bonding, and dimensional inconsistencies) despite extensive parameter optimization (see Supplementary Material S5 for details).

### 3.3 Steam sterilization test results

The materials were thoroughly investigated for surface altering before and after steam sterilization. After autoclaving, visible cracks were observed in the test samples made of SirayaTech Build Sonic Grey, SirayaTech Craft Ultra Clear, Phrozen Aqua Snow Grey, Phrozen Aqua Hyperfine Graphite with the cracks propagating along the layer lines through the sample discs whereas Phrozen Protowhite Rigid was bent along the cross section. As a result, these materials were excluded from further testing.

To verify that this exclusion reflected intrinsic material behaviour rather than insufficient curing under the standardised 20 min protocol, Phrozen Aqua Hyperfine Graphite was additionally tested at 10, 20, and 30 min UV-curing, the last matching the manufacturer-recommended post-cure exposure of 30 min. All three curing conditions produced the same layer-line cracking pattern after autoclaving, confirming that the failure mode is intrinsic to the material’s response to steam sterilisation. The microscopic images of the test samples can be found in the Supplementary Material S15.

### 3.4 Mechanical testing

Mechanical testing was performed to evaluate the structural integrity of preferred 3D-printed materials for biotechnological applications. Shore D hardness test results can be influenced by factors such as material thickness and testing spot selection. While Shore D hardness provides valuable information about surface properties and potential brittleness, it can also serve as an indicator of print quality uniformity across test samples (Mahmud et al., 2025; Mohammed and Mohammed, 2024). It is complemented by 3-point bending test and stability evaluations after post printing processes and autoclaving to assess the functional durability.

#### 3.4.1 Shore D hardness test

**FIGURE 3:**
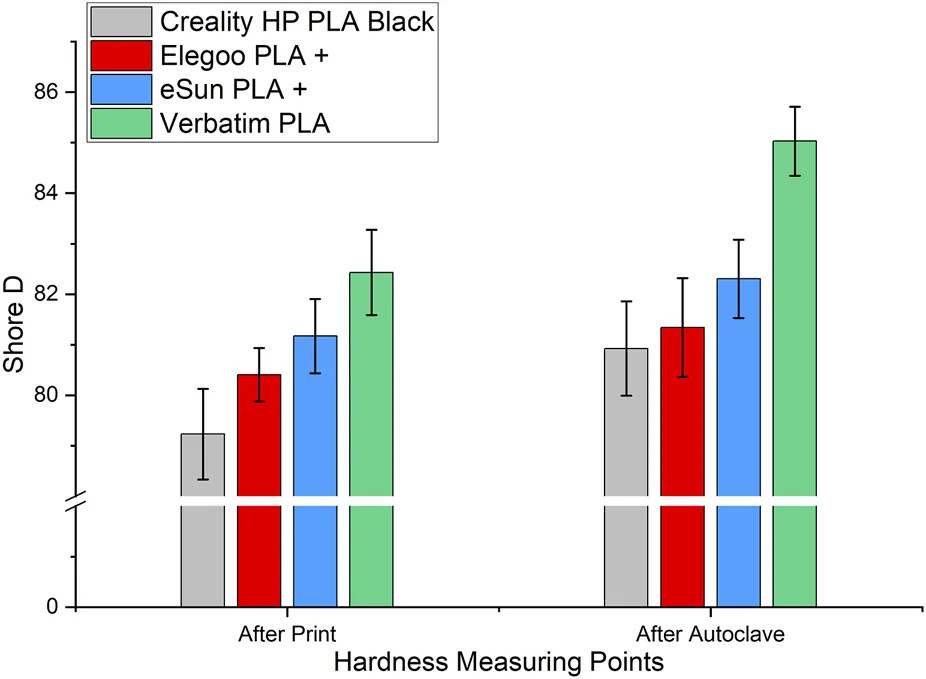
Results of Shore D hardness test for FDM-printed materials tested after printing and after autoclaving with standard deviation (set of triplicates with N = 8 each).

Mean post-printing values, as shown in Figure 3, ranged from 79.2 D ± 0.9 D (Creality PLA HP Black) to 82.4 D ± 0.8 D (Verbatim PLA Blue), indicating consistently high surface hardness among all brands. After autoclaving, hardness values increased across all samples, with Creality PLA HP Black at 80.9 D ± 0.9 D, Elegoo PLA + Grey at 81.3 D ± 0.9 D, eSun PLA + White at 82.3 D ± 0.8 D, and Verbatim PLA Blue at 85.0 D ± 0.7 D. The deviation of the measurements was in a comparable range for all materials and at different states of test.

**FIGURE 4:**
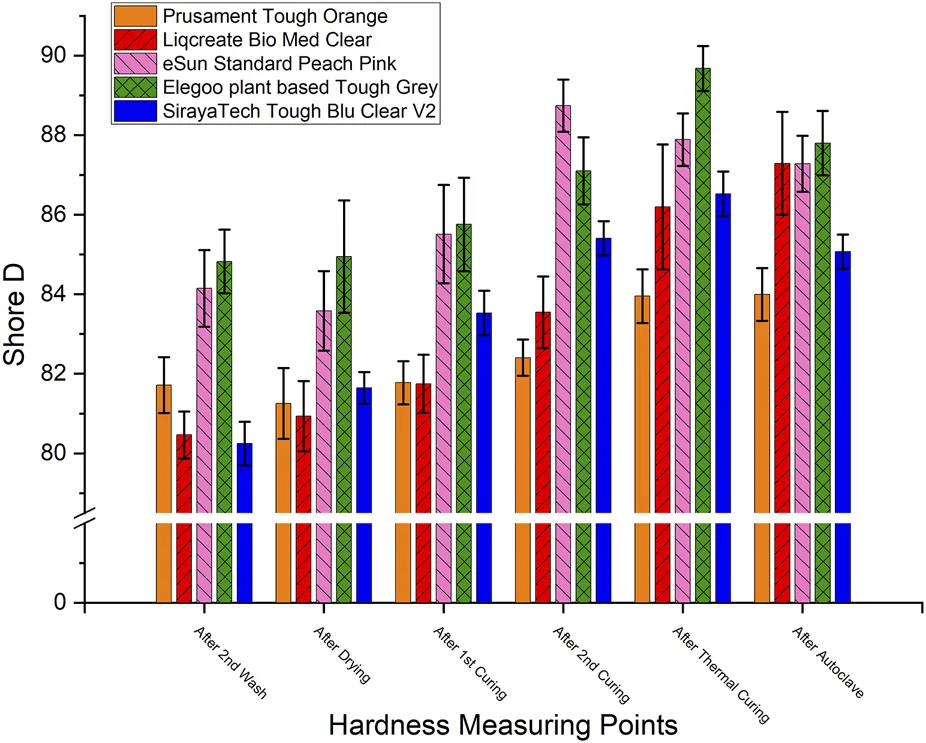
Results of Shore D hardness test for SLA-printed materials tested after each post-printing process and after autoclaving with standard deviation (set of triplicates with N = 8 each).

The photopolymer resins shown in Figure 4 generally exhibited an increase in Shore D hardness throughout the post-processing sequence, particularly from the initial IPA-washed state to the final thermally cured state. Prusament Tough Orange demonstrated the most consistent hardness profile, increasing gradually from 81.7 D (IPA wash) to 84.0 D (thermal cure), while maintaining a low standard deviation below ±0.9 D across all stages. Liqcreate Biomed Clear exhibited the largest overall hardness increase, rising from 80.5 D to 87.3 D after thermal curing, but notably showed increased variability in the final two states (thermal curing and autoclaving), reflecting reduced uniformity under prolonged thermal exposure. eSun Standard Peach Pink reached its maximum hardness after double-sided curing (88.8 D) but subsequently decreased to 87.3 D after thermal curing and autoclaving. Elegoo plant-based Tough Grey achieved the highest absolute hardness values after thermal curing (89.7 D), demonstrating strong post-processing hardening from the initial 84.8 D state. However, hardness decreased by 1.8 D following autoclave. Lastly, SirayaTech Tough Blu Clear V2 displayed a linear hardening trend from 80.2 D to 86.5 D with consistent reproducibility (standard deviation of ±0.43–0.56 D), then declined to 85.1 D post-autoclaving, showing modest but consistent sensitivity to sterilization.

#### 3.4.2 3-Point bending test

The results of the 3-point bending tests conducted on sample parts printed from both PLA filaments and photopolymer resins, as described in 2.6. The test shows the change in their flexural strength before and after autoclaving. The numerical values of this test can be found in the Supplementary Material S8.

All FDM-printed tested samples from Creality PLA HP Black, Elegoo PLA + Grey, and eSun PLA+ White remained intact after autoclaving and exhibited deformation under load without fracture. In contrast, the Verbatim PLA sample showed bending deformation after autoclaving but similarly only experienced flexural deformation during testing. The mechanical assessment of autoclaved photopolymer resins through 3-point bending tests revealed varying mechanical stability among the materials. Prusament Tough Orange and Elegoo plant-based Tough Grey exhibited minor surface cracking along the layer lines after autoclaving, which was accompanied by fracture upon bending. SirayaTech Tough Blu Clear V2 maintained structural integrity, remaining intact post autoclaving and showing only deformation without fracture underload. In contrast, Liqcreate Bio Med Clear and eSun Standard Peach Pink maintained intact surfaces post-autoclaving but fractured during bending tests, indicating reduced flexibility after autoclaving.

**FIGURE 5:**
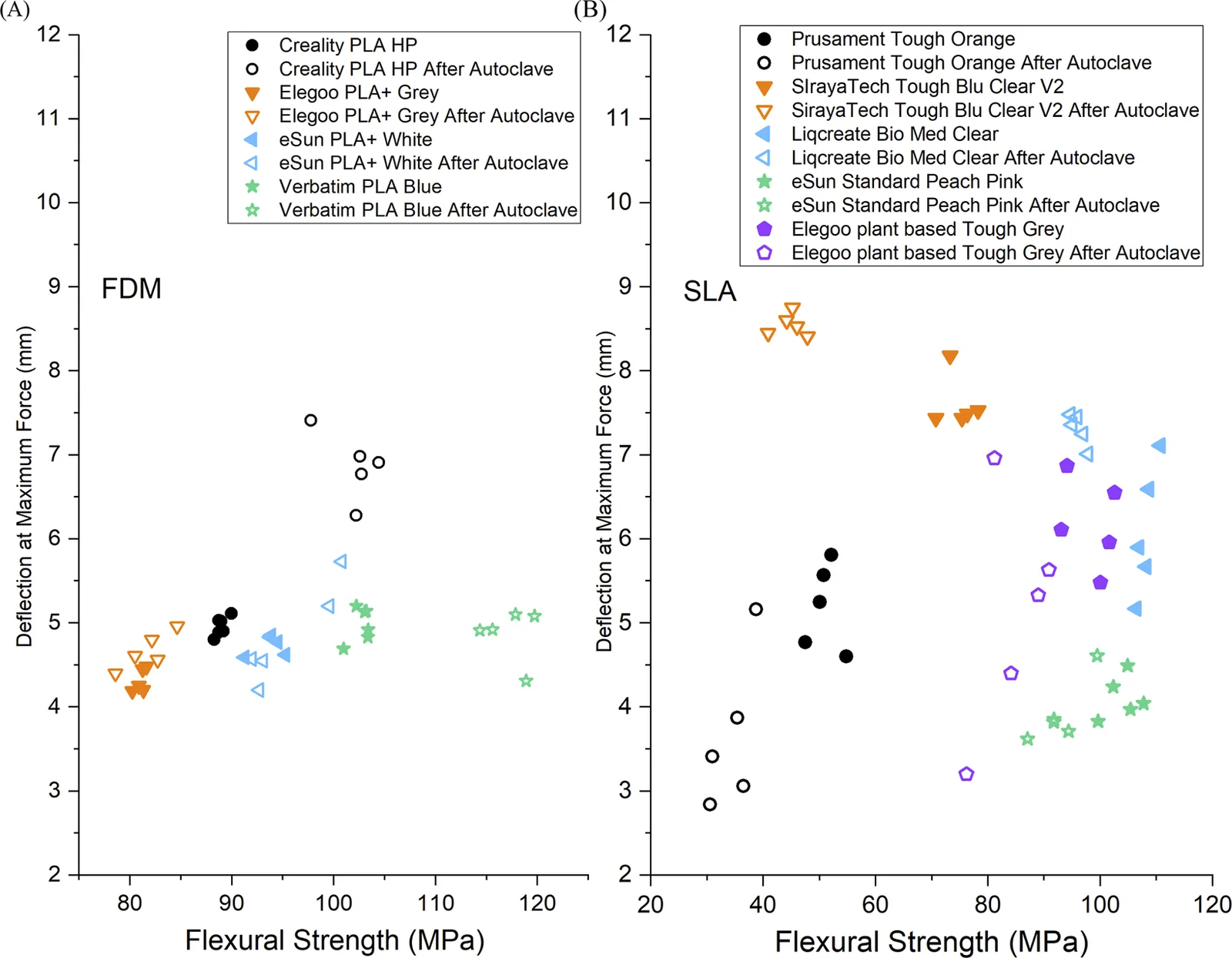
Scatter plot of flexural strength vs. deflection at maximum force for test samples printed from **(A)** PLA filaments (FDM, left) and **(B)** photopolymer resins (SLA, right) (N = 5).

Figure 5 illustrates flexural strength vs. maximum deflection for four PLA filaments and five photopolymer resins before/after autoclaving. For PLA filaments, Creality PLA HP Black showed highest gains (+13 MPa strength, +2 mm deflection from 90 ± 0.6 to 102 ± 2.5 MPa, 5 ± 0.1 to 7 ± 0.4 mm), while Elegoo PLA + Grey (81 ± 0.5 to 82 ± 2.3 MPa, ∼4–5 mm deflection) and Verbatim PLA (103 ± 0.9 to 117 ± 2.3 MPa, ∼4.5–5.5 mm) had increase in bending strength but less change in deflection. For photopolymer resins, Prusament Tough Orange had biggest degradation (−17 MPa strength, −1.5 mm deflection: 51 ± 2.7 to 34 ± 3.6 MPa, 5.2 ± 0.5 to 3.7 ± 0.9 mm) whereas SirayaTech Tough Blu Clear V2 gained deflection (+1 mm: 75 ± 2.9 to 45 ± 2.6 MPa, 7.6 ± 0.3 to 8.6 ± 0.1 mm). Liqcreate BioMed Clear, eSun Standard Peach Pink, Elegoo Plant-Based Tough Grey showed moderate responses. Among the FDM filaments, Elegoo PLA + Grey formed the high-strength group due to its stability in bending strength and deflection after testing, while Verbatim PLA and eSun PLA + White exhibited increase strength improvements with less changes in deflection, forming a distinct intermediate-strength cluster. For photopolymer resins, the values emphasize two visible clusters in the scatter plot: a lower-strength group formed by Prusament Tough Orange and SirayaTech Tough Blu Clear V2 after autoclaving, and a higher-strength group consisting of Liqcreate BioMed Clear, eSun Standard Peach Pink, and Elegoo plant-based Tough Grey that retain comparatively higher flexural strength with moderate deflection.

### 3.5 Biocompatibility test

**FIGURE 6:**
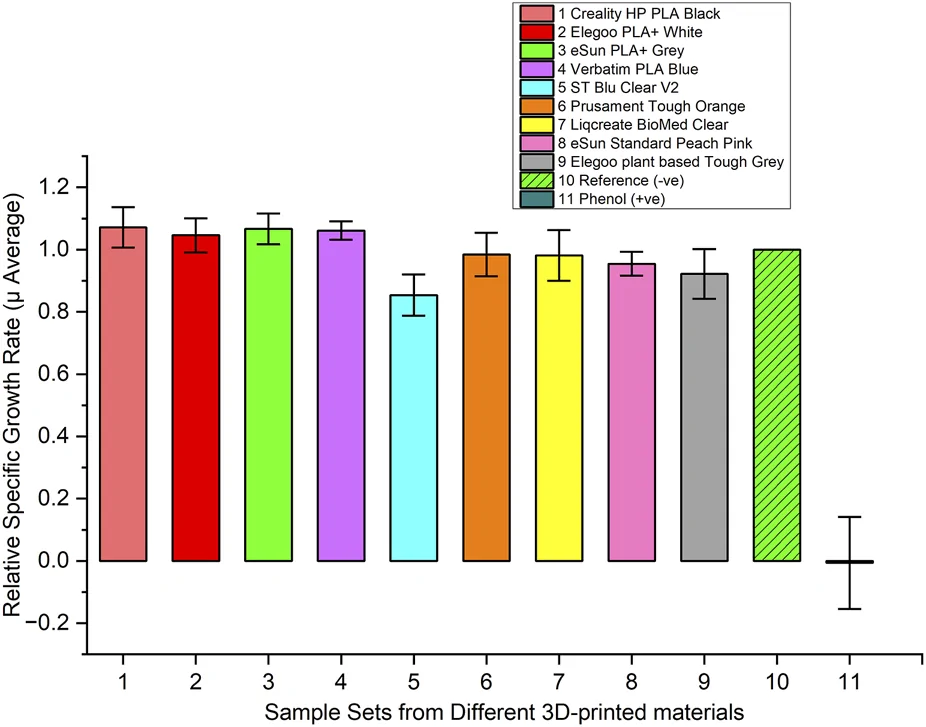
Bar chart of relative specific growth rates normalized to growth of the reference (−ve)in standard KMM medium at 37°C shaking at 200 rpm with respect to the reference (−ve) (p ≥ 0.05, N = 6).

Two independent sets of triplicate analyses were performed to evaluate the consistency and reproducibility of results. In these analyses as shown in Figure 6, the relative specific growth rate of each test sample is determined growth rates normalized to the negative control (-ve) of each test as reference material. Growth characteristics for each sample are further assessed, and the results are presented with associated standard deviations.

Biocompatibility testing as revealed in Figure 6 different 3D-printed material-specific effects of PLA and resin materials on *E. coli* growth relative to the reference. PLAs consistently exhibited bacterial growth support, with relative growth rates exceeding the growth of the reference. Notably, the relative growth of Creality HP PLA Black (+7.1%), Elegoo PLA + Grey (+4.6%), eSun PLA + White (+6.7%), Verbatim PLA Blue (+6. %) with respect to the reference. Among resins, Prusament Tough Orange (−1.6%), Liqcreate BioMed Clear (−1.9%), eSun Standard Peach Pink (−4.5%), SirayaTech Tough Blu Clear V2 (−14.6%) and Elegoo plant-based Tough Grey (−7.8%) exhibited a reduction in microbial growth when compared with the reference for relative specific growth rate. The phenol positive (+ve) control confirmed inhibition of growth, displaying strong bacterial growth suppression in all tests. Brittleness after these procedures was observed in test samples made from Verbatim PLA Blue.

### 3.6 Application of 3D-printed shake flasks

This section demonstrates the practical utility of the evaluated 3D-printed materials by showing their performance in microbial cultivation experiments. For this purpose, 100 mL shake flask designs were created in CAD based on standard glass labware which is analogous to Satzer and Achleitner (2022). The designs were adapted with increased wall thickness of 3 mm (Supplementary Material S14) to ensure reliable printability.

#### 3.6.1 Optical density comparison of *Saccharomyces cerevisiae* in glass and 3D-printed shake flask

A study was conducted to validate microbial growth in shake flasks printed from Creality PLA HP Black compared to standard glass shake flasks.

**FIGURE 7:**
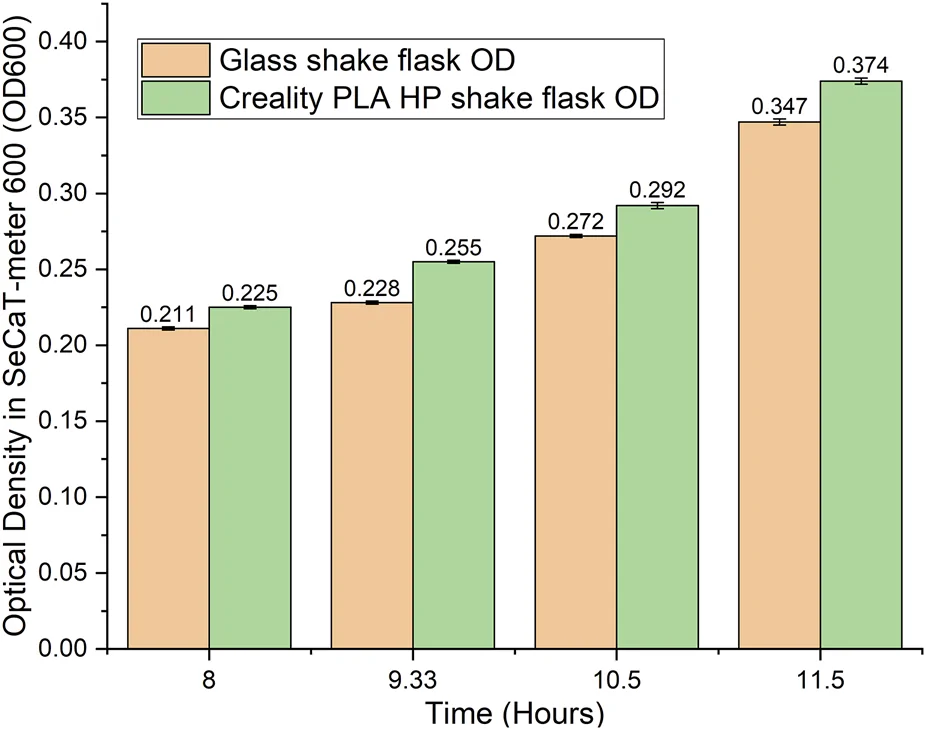
Measurements with SeCaT-meter 600 of cultivation of yeast in glass and Creality PLA HP made 3D-printed shake flasks over time (N = 3).

For each time point, triplicate OD600 measurements (found in the Supplementary Material S13) were taken for both glass and 3D-printed shake flask culture, as shown in Figure 7. The OD600 increased steadily in both shake flasks throughout the cultivation period. At each time-point, cultures grown in Creality PLA HP Black flasks exhibited slightly higher OD600 values than those in glass flasks. The differences between flask types remained small, and error bars were minimal, reflecting low variability.

#### 3.6.2 Microbial cultivation using 3D-printed 100 mL shake flasks with *Escherichia coli*

3D-printed shake flasks were fabricated using polymers identified through prior screening: Elegoo PLA + Grey, Verbatim PLA Blue, SirayaTech Tough Blu Clear V2, Liqcreate BioMed Clear, eSun Standard Peach Pink, and Elegoo plant based Tough Grey and Prusament Tough Orange. Prior to cultivation, all shake flasks were autoclaved at 121 °C for 20 min to ensure sterility. Post-autoclave inspection revealed perpendicular minor cracking of flask walls in eSun Standard Peach Pink and Elegoo Plant-Based Tough Grey vessels. In contrast, Prusament Tough Orange exhibited severe base cracking with total loss of medium. Therefore, this material was excluded from further shake flask applications despite its favourable biocompatibility results. Microbial cultivation was performed using autoclaved Elegoo PLA + Grey, Verbatim PLA Blue, SirayaTech Tough Blu Clear V2, Liqcreate BioMed Clear, eSun Standard Peach Pink, and Elegoo plant based Tough Grey shake flasks. The tested polymers supported bacterial growth without observable loss of medium, affirming the feasibility of selecting PLA filaments and photopolymer resins for shake flask fabrication in microbial biotechnology applications.

**FIGURE 8:**
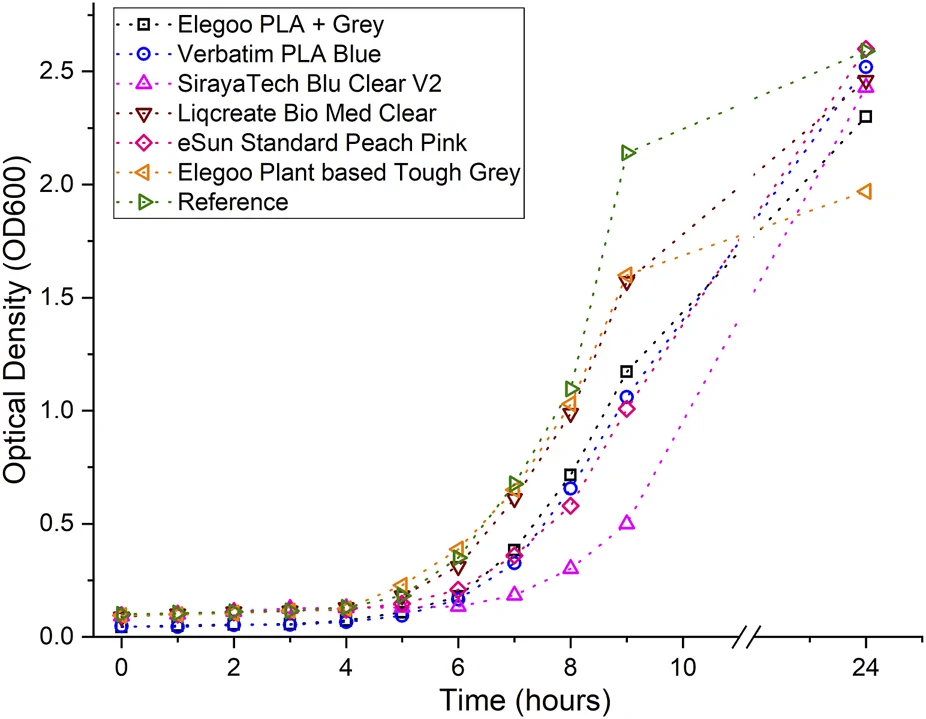
Growth curve of *Escherichia coli* in KMM at 37 °C and 220 rpm with 100 mL shake flasks 3D-printed from different materials.

The analysis of the growth curves, shown in Figure 8, shows that all SLA-printed shake flasks fabricated from Elegoo PLA + Grey, Verbatim PLA Blue, SirayaTech Tough Blu Clear V2, Liqcreate BioMed Clear, eSun Standard Peach Pink and Elegoo plant-based Tough Grey supported +4%, +10%, −15%, −8%, −18% and −18% (refer to “Datasets” in OSF storage) *E. coli* growth when normalized to the reference respectively (Ghalot et al., 2025). The Liqcreate BioMed Clear flask remained structurally intact even after autoclaving eleven times, demonstrating mechanical resilience and autoclavability. In contrast, the SirayaTech Tough Blu Clear V2 flask, eSun Standard Peach Pink and Elegoo plant-based Tough Grey developed cracks after autoclaving along the layer lines after conducting the application and washing. The flasks printed from PLA filaments lost their structural integrity and turned brittle after microbial cultivation. These results indicate that while all six materials tested here can support bacterial cultivation, Liqcreate BioMed Clear combines excellent biocompatibility for microbial cultivation with high durability under repeated autoclaving.

Table 1 summarizes the performance of 3D-printed materials based on their printability, mechanical properties, autoclave resistance, biocompatibility, and their application in microbial cultivation using shake flasks.

**TABLE 1:** Performance summary of 3D-printed materials: printability, mechanical test, autoclave test, biocompatibility and application of shake flasks for microbial cultivation.

Method of 3D-printing	Printability	Autoclave test	Bio-compatibility test w.r.t ref.	3D-printed brand	Hardness test	3-point bending test		Application of 3D-printed shake flasks
						Change in deflection	Change in strength	
FDM	✓	✓	+7.1%	Creality PLA HP Black	+2.0%	+38.5%	+14.5%	Single use****
	✓	✓	+4.6%	Elegoo PLA + Grey	+1.1%	+8.2%	+0.8%	Single use****
	✓	✓	+6.7%	eSun PLA + White	+1.5%	+2.5%	+1.9%	Single use****
	—	—	—	colorFabb PLA HP White	—	—	—	—
	✓	✓	+6.1%	Verbatim PLA Blue	+3.0%	−2.0%**	+14.2%	Single use****
SLA	✓	✓	−14.6%	SirayaTech Blu Clear V2	−0.4%	+12.3%	−40.0%	Single use****
	✓	—	—	SirayaTech Build Sonic Grey	—	—	—	—
	✓	—	—	SirayaTech Craft Ultra Clear	—	—	—	—
	✓	—	—	Phrozen Protowhite Rigid	—	—	—	—
	✓	—	—	Phrozen Aqua Snow Grey	—	—	—	—
	✓	—	—	Phrozen Aqua Hyperfine Graphite	—	—	—	—
	✓	✓	−1.9%	Liqcreate Bio Med Clear	+4.5%	+19.9%	−11.2%	Multiple use
	✓*	✓	—	Liqcreate Premium Tough	—	—	—	—
	—	—	—	Liqcreate Tough X	—	—	—	—
	✓	✓	−1.6%	Prusament Tough Orange	+1.9%	−29.5%	−32.5%	-***
	✓	✓	−4.5%	eSun Standard Peach Pink	−1.6%	−4.7%	−10.6%	Single use****
	✓	✓	−7.8%	Elegoo plant based Tough Grey	+0.8%	−17.6%	−14.2%	Single use****

The percentage changes in Shore D hardness, deflection, and strength test values are calculated by comparing measurements taken before and after autoclaving.

* Printable at very slow speeds.

** Deformed right after autoclaving.

*** Cracks developed after autoclaving.

**** Cracks developed after application.

## 4 Discussion

The output of this research highlights the effectiveness of the developed systematic material selection method for identifying suitable 3D-printed materials for microbial biotechnology applications. The screening was conducted for five PLA filaments and twelve photopolymer resins in order to develop and validate a standardized test method for assessing 3D-printed materials for biotechnology applications which were validated through application of a shake flask. The dual-step process of material validation enabled identification of process limitations and optimized parameters for the actual components used in laboratory-scale applications.

The iterative approach was guided by findings from Satzer and Achleitner (2022), Maker Trainer community and manufacturer recommendations which was the starting point for print parameter selection and underscored the value of model-specific optimization (Satzer and Achleitner, 2022; Achleitner et al., 2024; Maker Trainer, 2022). Larger objects can pose unique challenges in terms of print parameters and post-processing, especially for photopolymer resins, while for PLA filaments, infill percentage and pattern strongly influenced the wall permeability (Satzer and Achleitner, 2022; Gordeev et al., 2018). Similarly, SLA printing benefits from carefully balanced exposure conditions to achieve optimal crosslinking and surface homogeneity, impacting the mechanical resilience of printed parts. These findings underline the importance of balancing print speed and process duration to ensure viable manufacturing, supporting the exclusion of materials like ColorFabb PLA HP White, Liqcreate Tough X and Liqcreate Premium Tough due to slow speeds and print instability (Panico et al., 2025; Husna et al., 2024).

Following printing, the steam sterilization test served as an essential first screening to ensure material suitability for sterile biotechnological use. This test was particularly decisive, as out of 17 tested materials, only ten survived the autoclaving process without visible defects. Photopolymer resins such as SirayaTech Sonic Grey, SirayaTech Craft Ultra Clear, Aqua Snow Grey, and Aqua Hyperfine Graphite, along with Liqcreate Tough X, exhibited cracking that propagated through the diameter of the samples, and some layers delaminated, leading to failure. The test sample of Phrozen Protowhite Rigid was bent after the autoclave test. Mechanical evaluation using Shore D hardness and 3-point bending was prioritized before biocompatibility screening because these tests are faster, cost-effective, and filter out candidates unlikely to withstand process stresses. While 3-point bending tests were performed using the Instron 5564 device to ensure accurate and reproducible results, this equipment is costly and may not be readily accessible. Importantly, the mechanical tests did not lead to exclusion of any of the autoclave-resistant materials, as no minimum mechanical threshold could be defined that would reliably predict downstream performance from just a single biotechnology application. Thus, all materials that survived sterilization proceeded to biocompatibility evaluation.

The observed cracking post-autoclaving suggests material degradation induced by exposure to high temperature and pressurized steam. Such phenomena reflect hydrolytic and thermal stresses compromising resin integrity, consistent with prior findings on photopolymer resins exhibiting loss of mechanical robustness under steam sterilization conditions (Kronenberger et al., 2025). This underscores the importance of evaluating thermal and moisture stability after curing of photopolymer resins (Brooks and Yadavalli, 2025; Mahmud et al., 2025; Katheng et al., 2021). As materials with high post-cure sensitivity fail at the autoclave stage and are filtered out upstream of the biocompatibility read-out, the workflow is inherently robust against post-cure-sensitivity artefacts where cure-sensitive resins cannot produce spuriously degraded biocompatibility results, and cure-tolerant resins are not artificially favoured by the standardised protocol.

The reproducibility across Shore D hardness measurements before and after autoclaving in three test bodies demonstrates consistent measurement precision across materials, despite their differing hardness responses to thermal processing. The standardised post-processing protocol adopted in this study represents a compromise across materials rather than a per-material optimum, practitioners applying the proposed workflow are encouraged to fine-tune UV-curing and washing parameters for their specific printer, post-processing equipment, and target application once a candidate material has been shortlisted (Mahmud et al., 2025; Katheng et al., 2021). For PLA, it is literature known that autoclaving could induce molecular changes primarily due to heat and moisture exposure, leading to increased crystallinity (Neijhoft et al., 2023). This crystallization causes a rise in material hardness and rigidity but can also lead to brittleness and potential shrinkage (Mahmud et al., 2025; Zennaki et al., 2022). The change in Shore D hardness observed across the photopolymer resins during the post printing process could be attributed to ongoing crosslinking reactions facilitated by UV curing and annealing treatment (Mahmud et al., 2025; Guttridge et al., 2022; Bagheri and Jin, 2019). This trend aligns with research indicating that post-curing enhances the degree of polymerization and crosslink density, leading to increased material hardness and rigidity (Husna et al., 2024). Liqcreate Biomed Clear shows the largest overall hardness gain during post-processing (80.5 D to 87.3 D), potentially indicating enhanced crosslinking (Bagheri and Jin, 2019). However, the subsequent hardness drop after autoclaving in other 3D-printed materials may suggest sensitivity to steam-induced hydrolysis or thermal degradation, consistent with mechanisms observed across multiple resins (Oh et al., 2023; Bagheri and Jin, 2019; Kronenberger et al., 2025). The eSun Standard Peach Pink reached its maximum hardness after double-sided curing (88.8 D) with a decrease after thermal curing and autoclaving (87.8 and 87.3 D respectively), suggesting minor thermal softening likely due to heat and moisture exposure (Oh et al., 2023; Mahmud et al., 2025; Katheng et al., 2021). Elegoo plant-Based Tough Grey also exhibited high post-curing hardness but experienced a decline (1.8 D) following sterilization. SirayaTech Blu Tough Clear V2 showed a gradual and reproducible increase in hardness (from 80.2 D to 86.5 D), with only minor reduction after autoclaving, possibly due to thermal softening in the polymer network (Oh et al., 2023; Mahmud et al., 2025; Katheng et al., 2021). While Elegoo plant-based Tough Grey exhibited the highest cured hardness, its lower resistance to hydrothermal stress suggests potential brittleness upon repeated autoclave exposure (Brooks and Yadavalli, 2025; Mahmud et al., 2025; Zennaki et al., 2022). The overall hardness stability reveals that Prusament Tough Orange as the most stable material, showing minimal variance and resistance to both thermal curing and autoclaving. Liqcreate BioMed Clear demonstrated the lowest change in hardness profile across post-processing stages and maintained structural integrity through eleven repeated autoclaving cycles, suggesting superior durability under thermal and moisture stress. SirayaTech Blu Tough Clear V2 showed consistent hardness development with minimal hardness loss post-autoclaving, indicating relatively stable thermal behavior. The eSun Standard Peach Pink achieved uniform polymerization during post-processing but already showed a measurable reduction in hardness after thermal curing, with an additional decrease following autoclaving, indicating that both curing and steam-sterilization-related heat and moisture exposure contributed to the overall change. Elegoo plant-based Tough Grey reached the highest absolute hardness values after curing (89.7 D) but experienced notable hardness reduction post-autoclaving (84.0 D), which is consistent with the literature described broader pattern of hardness loss observed across multiple photopolymers under repeated sterilization.

The three-point bending test results reveal that autoclaving affects the flexural mechanical properties of PLA filaments differently depending on the material formulation from the manufacturer. Creality PLA HP Black and Verbatim PLA Blue showed increased flexural strength post-autoclaving, likely due to literature described enhanced interlayer adhesion or stress relief during heat treatment (Kumar et al., 2023; Zennaki et al., 2022; Klimczuk et al., 2024; Neijhoft et al., 2023). In contrast, Elegoo PLA + Grey and eSun PLA + White maintained relatively stable flexural properties, indicating good sterilization resilience. The annealing (thermal curing) effect enhances its mechanical strength, often compensating for chain scission (Klimczuk et al., 2024; Zennaki et al., 2022). In contrast, photopolymer resins, which cure via cross-linked networks, do not experience crystallization and tend to suffer from more pronounced hydrolytic degradation and polymer chain scission under thermal and moisture stresses, leading to decreased flexural strength (Brooks and Yadavalli, 2025; Bagheri and Jin, 2019; Mahmud et al., 2025). Deflection behavior varied: SirayaTech Tough Blu Clear V2 and Liqcreate Bio Med Clear showed increased flexibility, suggesting matrix plasticization, whereas Prusament Tough Orange and Elegoo Plant-Based Tough Grey had a decrease in flexural strength and deflection. The eSun Standard Peach Pink showed stable deflection but reduced strength. The bending test reveals that autoclaving can benefit certain PLA filaments and are suitable for autoclave-tolerant applications. Photopolymer resins generally show a decline in flexural strength and deflection after autoclaving, suggesting degrading mechanical properties. These observations indicate that crosslink density and material formulation critically influence thermal and moisture stability under steam sterilization conditions (Bagheri and Jin, 2019).

After mechanical testing, microbial cultivation with *E. coli* assesses biocompatibility under conditions relevant to biotechnology (Achleitner et al., 2024; Licata et al., 2024). The proposed framework is methodologically organism-agnostic and can be adapted to other biotechnology by substituting the appropriate cultivation medium and inoculum. The specific microbial parameter recommendations reported here are, however, based on *E. coli* MG1655 as a reference organism, and re-screening with the target host is advised when applying the framework to organisms with differing sensitivities to leachates. Compared to the PE-HD control, tested materials showed material-specific effects on growth rate, indicating varying leachate impacts and initial stress responses (Hamill et al., 2020). PLAs consistently supported *E. coli* growth effectively, often exceeding the reference culture’s growth rates, indicating their suitability as microbial cultivation despite exhibiting material-dependent effects on growth kinetics. PLA degradation products, primarily lactic acid and its oligomers, can impact bacterial growth rates by serving as a metabolizable carbon sources that support microbial growth (Klimczuk et al., 2024; Hamill et al., 2020; Shalem et al., 2024). This highlights the importance of carefully evaluating the suitability of PLA-based materials for such applications, particularly in environments where microbial growth must be optimum. The alignment with literature on commercial PLA formulations showing high biocompatibility, biodegradability, and minimal toxic leachates when properly validated, positioning select variants as viable for biological applications (Pérez-Davila et al., 2022; Neijhoft et al., 2023). Some photopolymer resins showed comparable bacterial growth rates to the reference, reflecting better biocompatibility such as Prusament Tough Orange and Liqcreate BioMed Clear. However, other resins caused significant growth inhibition, likely due to cytotoxic components or leachates, as supported by studies highlighting the variability of resin biocompatibility depending on chemical composition and curing protocols (Guttridge et al., 2022; Brooks and Yadavalli, 2025; Oh et al., 2023; Licata et al., 2024). Although direct chromatographic quantification of residual leachates (e.g., via GC-MS or LC-MS) was beyond the analytical scope of this method-validation study, the standardised washing and post-curing protocol applied here (Section 2.3) is consistent with the post-processing strategies shown by Brooks and Yadavalli (2025), Mohammed and Mohammed (2024), and Oh et al. (2023) to substantially reduce residual monomer content from SLA-printed parts (Brooks and Yadavalli, 2025; Oh et al., 2023; Mohammed and Mohammed, 2024). Chromatographic extractables profiling were identified as a valuable follow-up study.

The modified Gompertz sigmoidal model was selected as the quantitative approach for fitting growth curves in this research based on both statistical and biological relevance to the growth dynamics inherent in microbial cultivation (Wang and Guo, 2024). Asymmetric architecture more faithfully reproduces the biological reality of bacterial adaptation kinetics, where cultures undergo a relatively brief lag phase before entering a steep exponential growth period, followed by a more extended plateau toward stationary phase. The asymmetry is particularly relevant when materials or their leachates exert partial growth inhibition, where the extended lag phase becomes an indicator of material-induced stress (leachates) before exponential growth kinetics are established (Wang and Guo, 2024; Zwietering et al., 1990). Material leachates, unreacted oligomers, or roughness-induced stress can delay exponential growth initiation without necessarily reducing the exponential growth rate once adaptation is complete (Oskui et al., 2016; Hamill et al., 2020). Empirical evidence (Section 3.2 and 3.3) further confirms that temperature and environmental perturbations affect growth rates, influencing model fitting quality. The measured OD data were fitted by the Gompertz model, even when using traditional calculation methods, demonstrating the reliability of this modelling approach for capturing microbial growth dynamics.

Despite prior screening for biocompatibility, several plastics exhibited limitations upon exposure to repeated autoclaving and application-relevant conditions. Specifically, materials such as Prusament Tough Orange failed to maintain structural integrity, displaying major crack where the base of the flask was completely separated from the body and consequent medium leakage leading to exclusion even though their biocompatibility test results were otherwise favourable. Certain shake flasks from materials such as eSun Standard Peach Pink and Elegoo plant based Tough Grey, demonstrated perpendicular cracking of flask walls after autoclaving but did not immediately compromise medium containment. Elegoo PLA + Grey and Verbatim PLA Blue turned brittle post microbial cultivation. These findings suggest that such materials could be repurposed for single-use, where mechanical resilience is less critical (Licata et al., 2024).

Moreover, the observed *E. coli* growth variations for Elegoo PLA + Grey, Verbatim PLA Blue, SirayaTech Tough Blu Clear V2, Liqcreate BioMed Clear, eSun Standard Peach Pink and Elegoo plant-based Tough Grey supported +4%, +10%, −15%, −8%, −18% and −18% when normalized to the glass shake flask reference respectively reveal that both mechanical performance and biocompatibility contribute to overall vessel suitability. These relative growth rates align well with the results from the leachate biocompatibility test, with the exception of eSun Standard Peach Pink and Elegoo plant-based Tough Grey, which showed deviations. This finding underscores the robustness of the proposed evaluation method, as six out of eight materials demonstrated consistent results between the leachate test and the shake flask application. These results highlight the importance of combining mechanical performance, biocompatibility, and microbial growth assessments to comprehensively evaluate vessel suitability for biotechnological applications. The reduced 18% *E. coli* growth reduction in eSun Peach Pink and Elegoo plant-based Tough Grey flasks possibly stems from cracking after autoclaving, which increases surface area (Kronenberger et al., 2025). This exposes internal uncured material, which was previously shielded within flask walls, likely allowing inhibitory leachates into the medium (Brooks and Yadavalli, 2025). This geometry-dependent test illustrates that the disc-based leachate test, while broadly predictive of intrinsic material biocompatibility, cannot anticipate every mode of post-sterilisation failure that may arise under application-specific wall geometries and mechanical conditions. Coupling the standardised leachate screening with an application-stage cultivation check therefore captures both the chemical and the geometry-driven contributions to microbial response, and the two stages together constitute the recommended use of the proposed workflow. Notably, Liqcreate BioMed Clear stood out due to its ability to withstand repeated autoclaving (nine cycles) without loss of structural integrity, as well as its consistent support of microbial growth, making it especially promising for reusable lab-scale cultivation platforms. Previous work confirms that additive manufacturing enables rapid iteration, tailored vessel geometry, and cost-efficient fabrication, but achieving robust performance under bioprocess conditions requires rigorous multi-stage validation (Raddatz et al., 2017; Achleitner et al., 2024). By subjecting the materials to functional testing under process conditions, the proposed method ensured that only those meeting all criteria, printability, sterilization test, mechanical integrity and biocompatibility, was considered a viable approach.

## 5 Conclusion

This research established a reproducible method to evaluate the 3D-printed materials for use in microbial cultivations. The method integrated printability assessment, autoclaving, and standardized mechanical (Shore D hardness and 3-point bending) tests before and after autoclaving with microbial biocompatibility tests. The methods applicability was demonstrated through the fabrication of watertight shake flasks printed by FDM and SLA, which showed similar cultivation outcome with *E. coli*, as the glass made flasks, confirming the practical relevance of the approach for designing functional, sterilizable components.

Among the tested materials, FDM-printed PLA filaments demonstrated suitable properties for biotechnological applications, with trade-offs between mechanical stability and post-sterilization durability. Creality PLA HP Black exhibited a 2.0% increase in hardness after autoclaving but experienced a notable 38.5% deflection increase, a substantial change that reflects post-sterilization embrittlement, alongside a 14.5% increase in flexural strength. This hardness gain, while modest in magnitude, coupled with increased deflection and strength, indicates material degradation that limits post-sterilization mechanical performance. In contrast, Elegoo PLA + Grey and eSun PLA + White variants showed greater stability, with shore D hardness changes of only +1.1 to +1.5% and flexural strength increase up to 2%, suggesting better preservation of mechanical integrity through the sterilization cycle. Across all PLA-based materials, microbial growth rates remained comparable to reference control, with variation between +4 and +7% confirming biocompatibility. These results indicate that FDM-printed PLA vessels can tolerate a single autoclaving cycle with acceptable retention of functional properties, making them viable for single-use applications under static or agitated cultivation conditions, though post-sterilization mechanical performance should be verified for specific operational demands. In contrast, SLA-printed photopolymer resins exhibited more variable behaviour. Liqcreate BioMed Clear outperformed other resins, showing a moderate hardness increase (+4.5%) and limited flexural strength loss (−11.2%), while maintaining good biocompatibility (change in specific growth rate −1.6% w.r.t reference). Although its cost (approximately €110 per kg) is higher than PLA (approximately €20), it is offset by mechanical robustness and resistance to hydrolytic degradation, favoring reuse since it could withstand autoclave conditions multiple times. SirayaTech Blu Clear V2 and Prusament Tough Orange demonstrated moderate stability after autoclaving, each evidencing strength losses exceeding 40% and reduced biocompatibility (−14.6% and −1.6%, respectively when compared to reference). Economical SLA alternatives such as Elegoo plant-based Tough Grey (−17.6% deflection, −14.2% bending strength) and eSun Standard Peach Pink (−4.7% deflection, −10.6% bending strength) displayed brittleness and reduced biocompatibility (−7.8% and −4.5% respectively w.r.t. reference) are thus recommended for single-use applications only. However, it is important to note that shake flasks made from these materials (Elegoo plant-based Tough Grey and eSun Standard Peach Pink) exhibited cracking during use, and validation experiments revealed lesser microbial growth rates compared to the leachate biocompatibility test. The leachate test reliably predicts microbial biocompatibility, but geometry-driven mechanical failure during autoclaving can introduce additional leachate-exposure and the application-specific validation stage is therefore an essential complement to the leachate screening, not an optional supplement. This highlights a critical limitation of these materials for long-term or reusable applications, emphasizing the need for further refinement of material selection criteria to ensure both mechanical stability and biocompatibility under conditions.

As batch stability of commercial PLA filaments and photopolymer resins cannot be ensured on the basis of currently available manufacturer datasheets, we recommend that practitioners re-run the proposed workflow for every new batch of material, at minimum the autoclave, Shore D hardness, and leachate biocompatibility steps, before adopting it for their intended application. Within a given project or production campaign, material should be sourced from a single batch where possible, and batch and lot identifiers should be reported routinely in methods sections to support reproducibility across the field. The workflow in this paper provides a reproducible framework for evaluating and comparing 3D-printed materials across different material batches and suppliers, enabling systematic assessment of batch-to-batch variability in post-sterilization mechanical integrity and microbial biocompatibility. By applying consistent testing protocols, researchers can quantify how formulation differences between batches affect their application performance, thereby establishing which material specifications and production lots are suitable for single or multi-use cultivation applications.

Collectively, these findings support all PLA + filaments as the most reliable and cost-effective choice for single use sterilization and cultivation, combining mechanical durability and biological compatibility. Among the PLA + materials tested, Elegoo PLA + Grey and eSun PLA + White exhibited better performance, demonstrating minimal changes in hardness, deflection, and bending strength after autoclaving, indicating their suitability for printing of single use shake flasks for microbial cultivation. The batch obtained of Liqcreate BioMed Clear is the most promising SLA resin for reusable components requiring higher resolution, multiple autoclavability with promising biocompatibility. Autoclaving emerged as the most detrimental step in the evaluation process. It eliminated half of the tested materials during initial screening due to cracking or significant mechanical degradation. Furthermore, in the validation experiments, autoclaving was again indicative of failure, as materials that passed the leachate biocompatibility test often exhibited poor microbial growth performance after autoclaving, even before cultivation began. This highlights the critical role of autoclaving in determining material suitability and underscores the need for robust material selection criteria that account for both mechanical integrity and biocompatibility under sterilization conditions. The presented method offers a quantifiable and reproducible pathway for assessing mechanical integrity with microbial biocompatibility, enabling informed material selection for 3D-printed vessels and prototype bioreactor components. 3D-printing of suitable small-scale bioreactors for specific applications within our research group will be pursued based on the validated material selection criteria and method developed in this research.

## Data Availability

The datasets presented in this study can be found in online repositories. The names of the repository/repositories and accession number(s) can be found in the article/Supplementary Material.
