# Supplementary material for: Development and validation of material selection method for cost-effective lab-scale 3D-printed platforms in biotechnology
Source: Front Bioeng Biotechnol. 2026 Aug 31;14:1850515. doi: 10.3389/fbioe.2026.1850515 (PMC13572646; doi:10.3389/fbioe.2026.1850515)
Supplement: Supplementary file 1 [file Supplementaryfile1.pdf]

Supplementary Material

OSF Repository Project DOI: https://doi.org/10.17605/OSF.IO/G97S2

1 Printing defects observed in FDM benchmarks

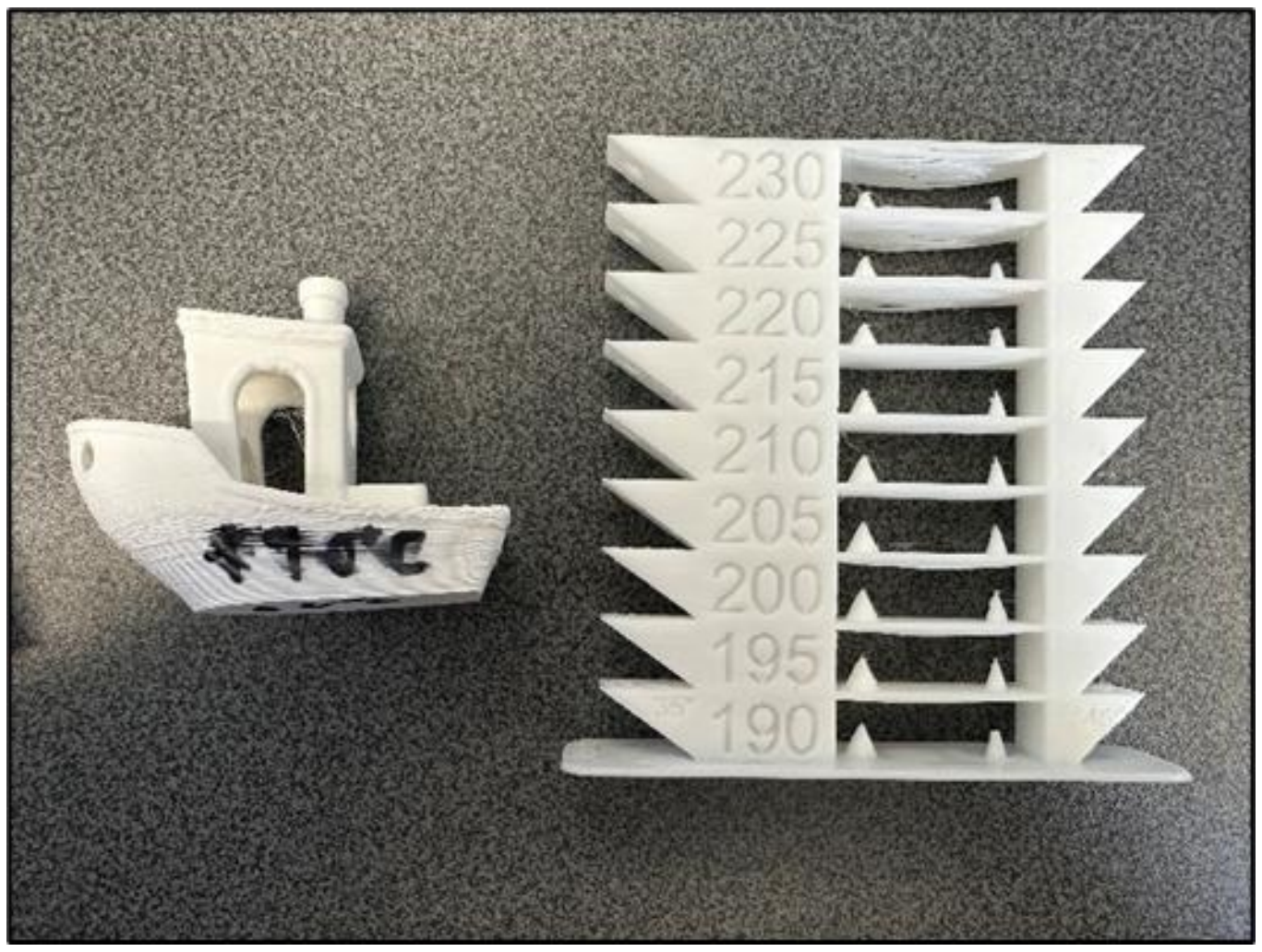

Supplementary Figure 1 Blobs and slits on boat (left), Overhang due to incorrect nozzle temperature (right)

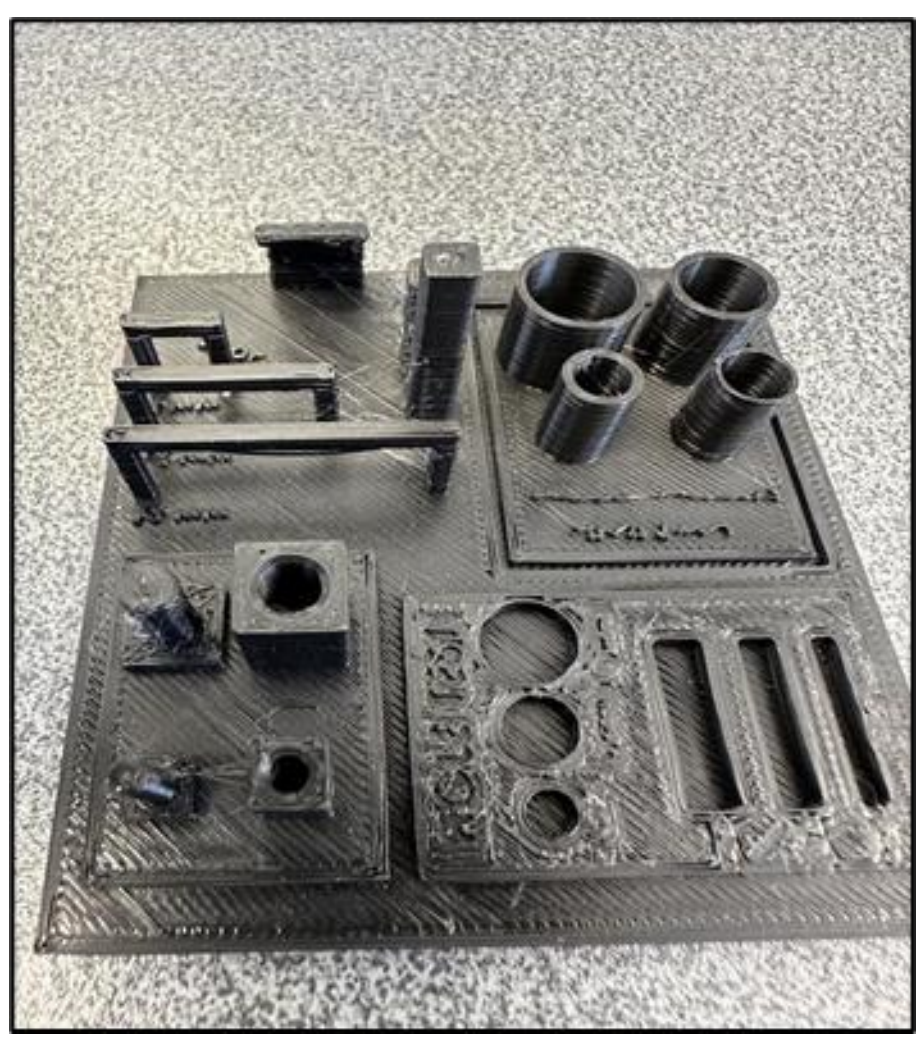

Supplementary Figure 2 Stringing failure in FDM printing

The important parameters which affect the quality of the printed parts are:

Printing speed, heat bed & nozzle temperature.

FDM print failures include poor first-layer adhesion where the print does not stick to the heated bed causing detachment; stringing, which leaves thin plastic threads on the model; and blobs or zits resulting from inconsistent material flow due to incorrect retraction settings, over-extrusion, or excessive nozzle temperature. More information about defects can be found e.g. on Simplify3D.

2 Printing defects observed in SLA benchmarks

The important parameters which affect the quality of the printed parts are:

Exposure time, initial exposure time and layer height

Each resin manufacturer provides proprietary print settings, however, since this research evaluates multiple resin brands, the common requirement is that all resins be compatible with a standard SLA printer.

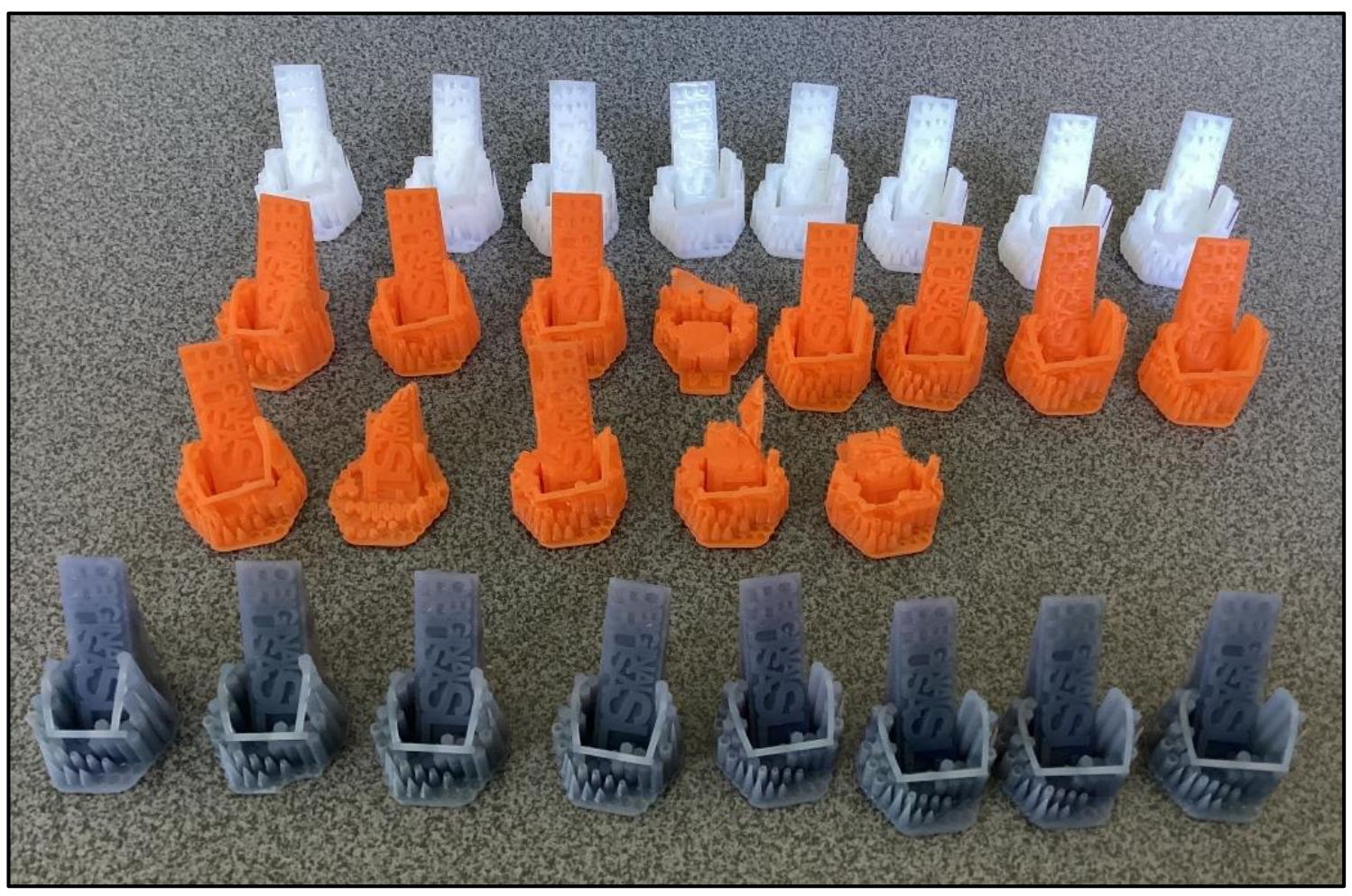

Supplementary Figure 3 Printability assessment of photopolymer resins with Prusa calibration object

To determine the optimal exposure times, the Prusa default SLA calibration object was used as the test specimen which enabled parallel printing of objects with incrementally different exposure times. Starting from the manufacturer-recommended parameters, exposure times were iteratively adjusted based on print quality outcomes. The Prusa calibration object found on Printables, includes features for assessing fine details such as small holes, pointed tips, aviators, and inscribed text, enabling comprehensive resolution evaluation. Different calibration prints at varying exposure times provided systematic insight into tuning to the best printing parameters.

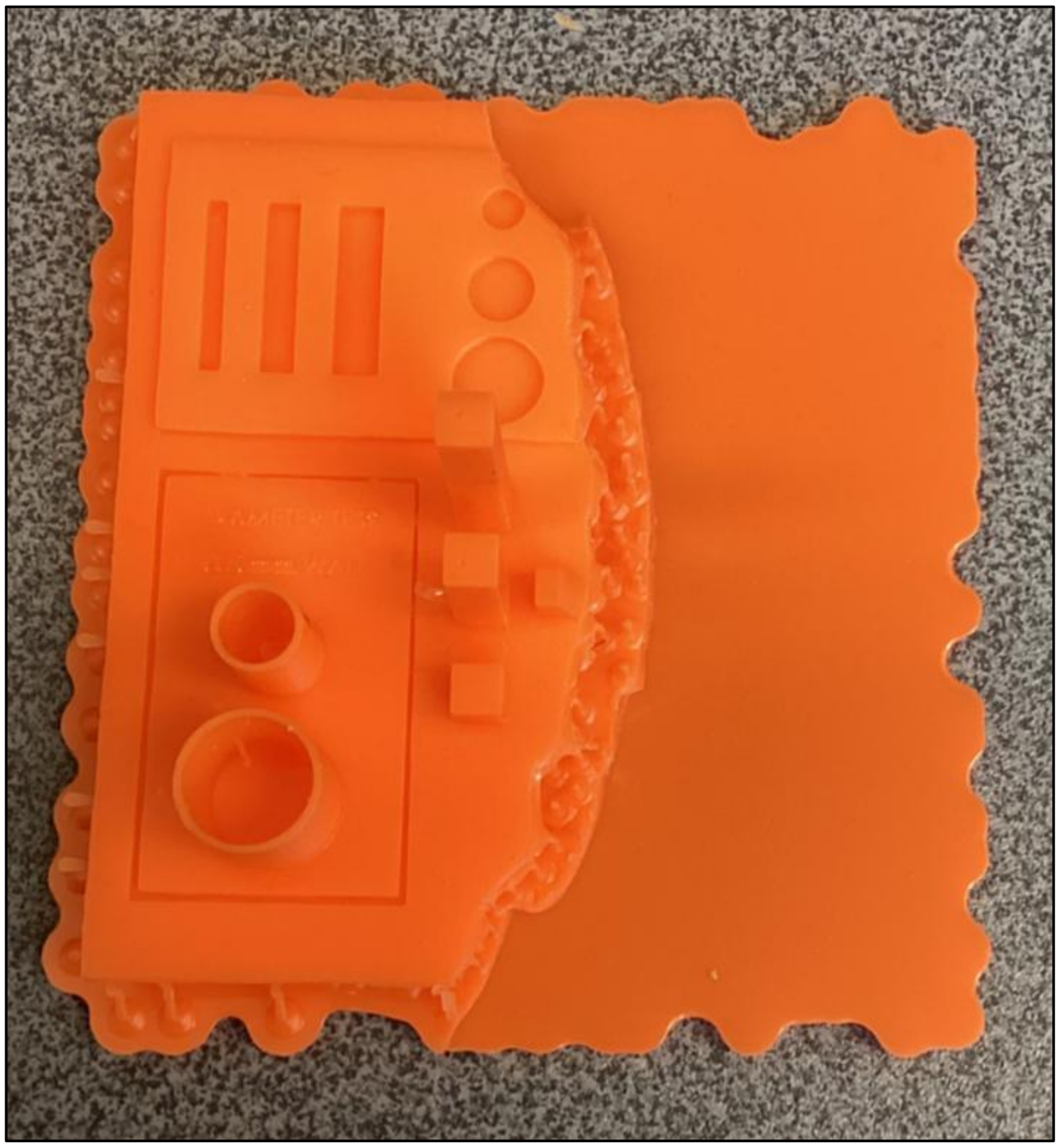

Supplementary Figure 4 Example for SLA print failure – Example of incorrect exposure time

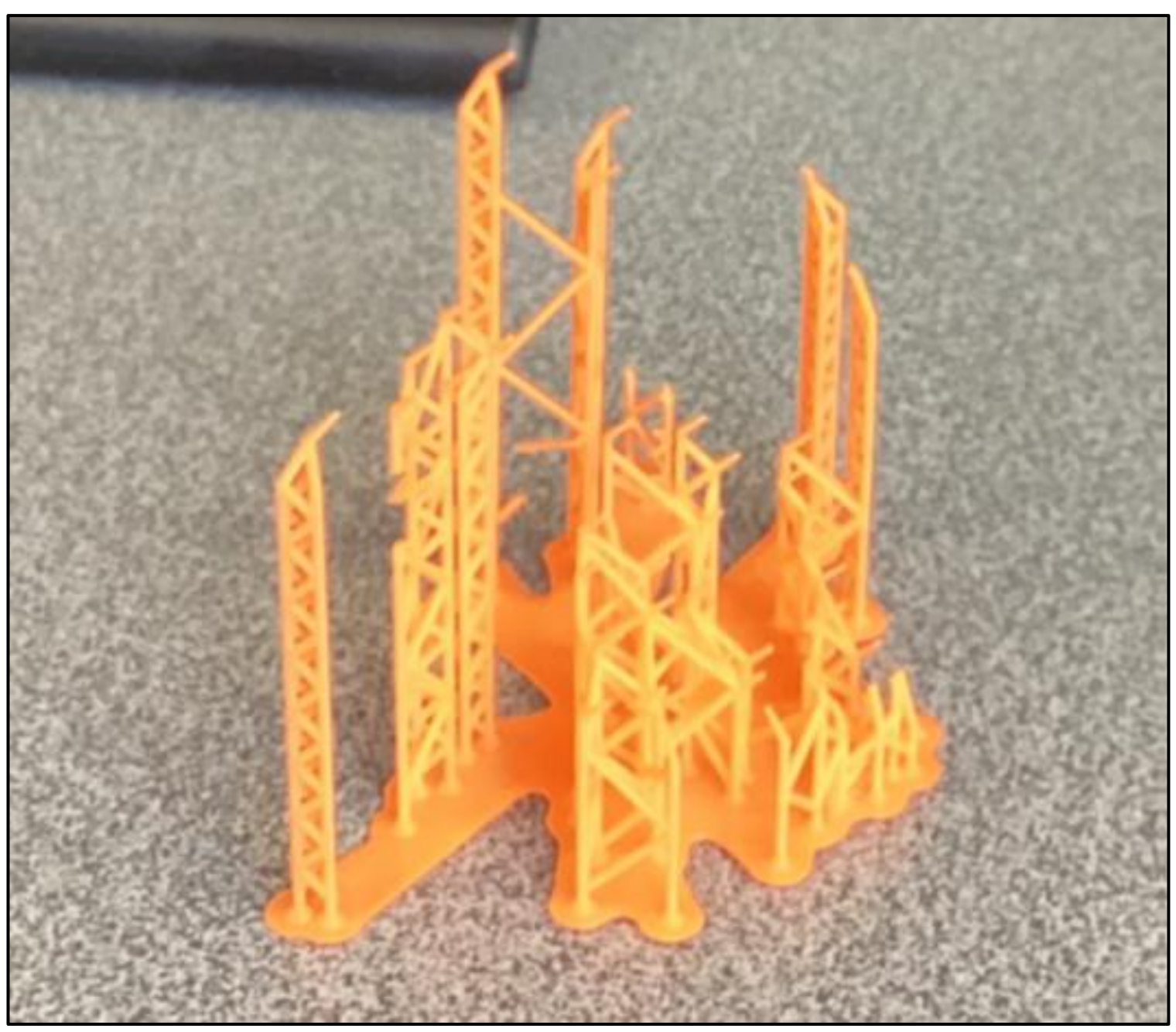

Supplementary Figure 5 Example for SLA print failure - Incorrect print orientation & exposure time

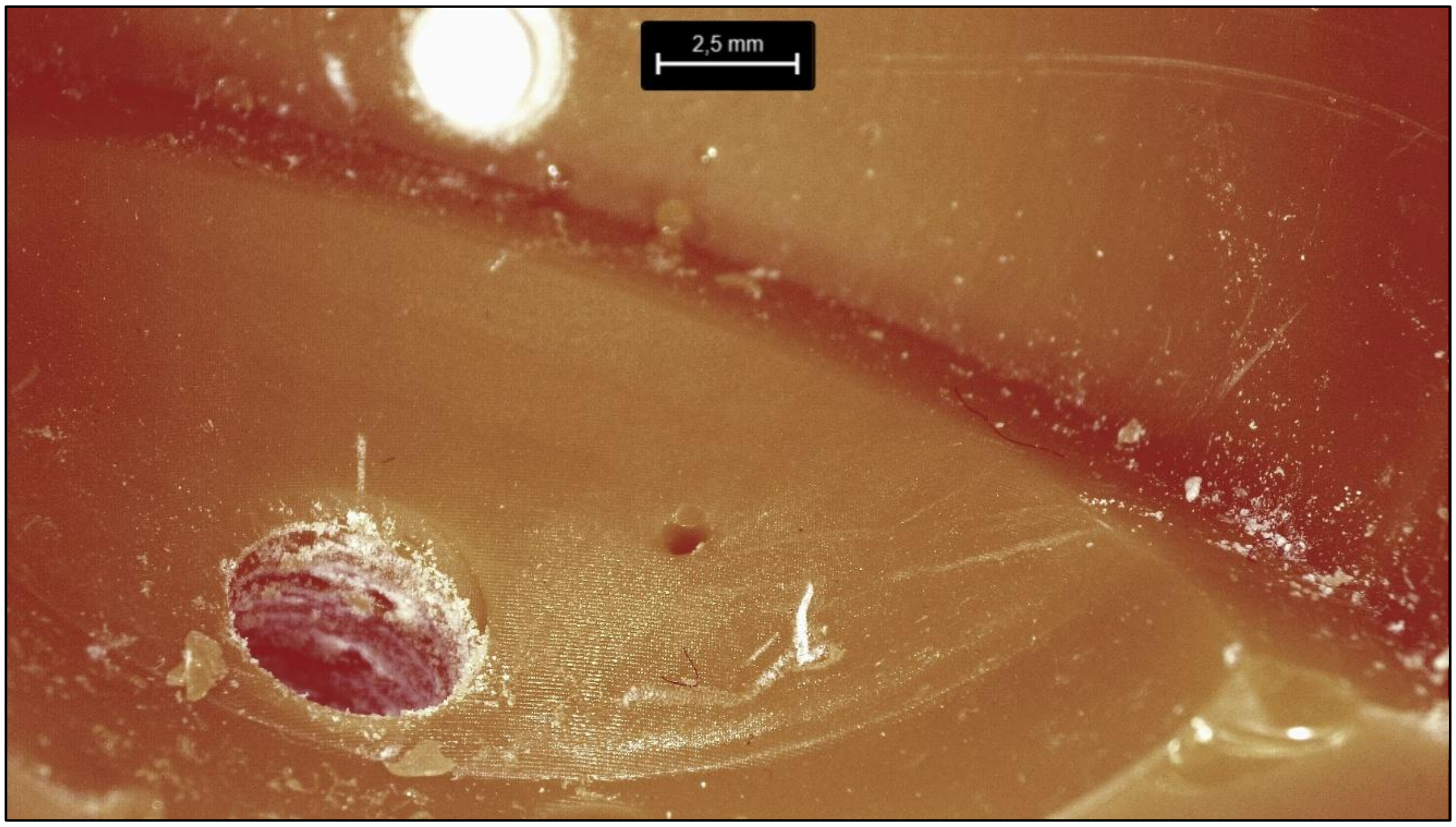

Supplementary Figure 6 Example for SLA post printing process failure (white residue) - Incorrect curing times & lower washing time with IPA

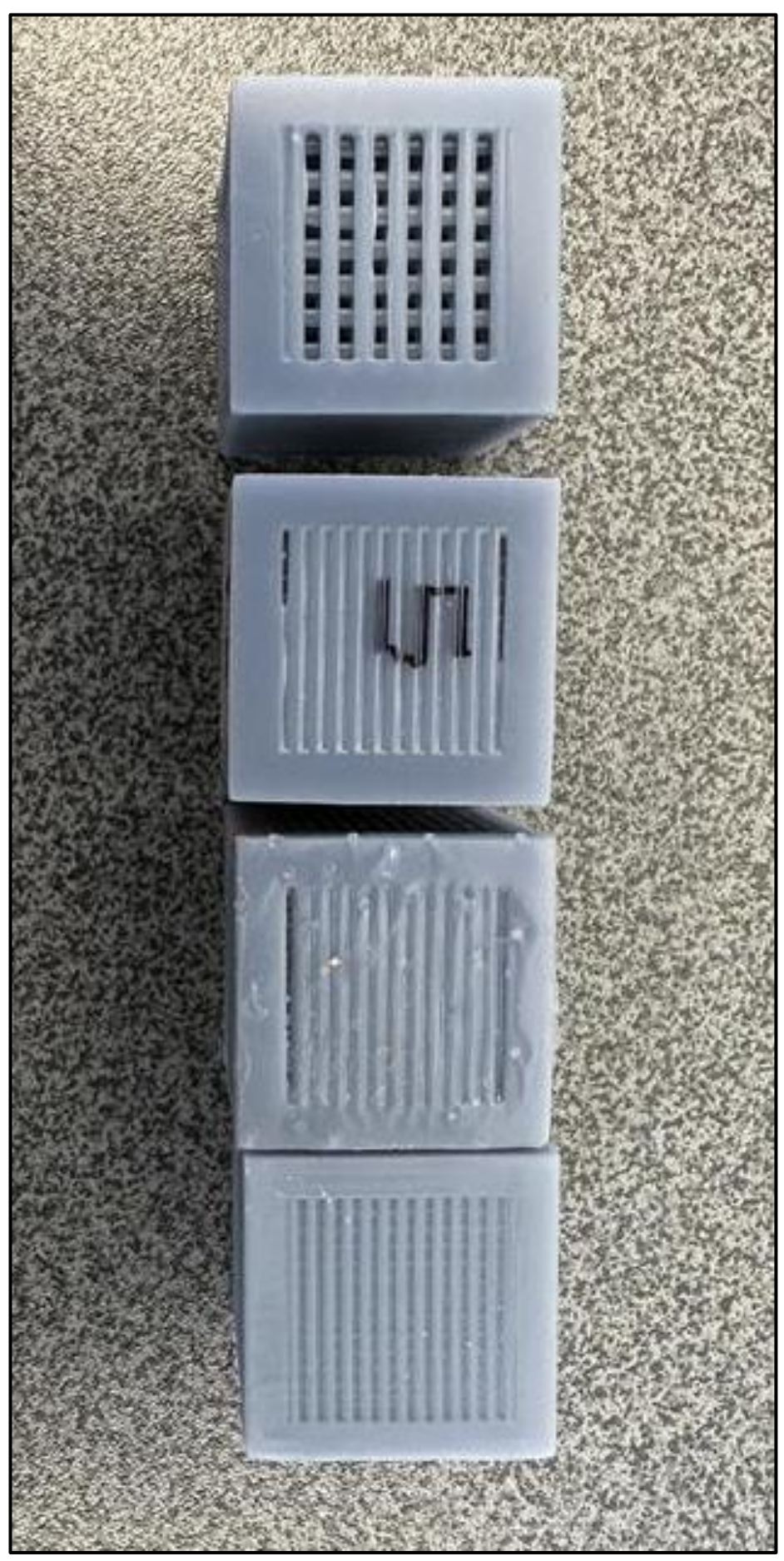

Supplementary Figure 7 Incorrect exposure time and post printing process failure - Difference between curing and overcuring during post printing process

SLA print failures involve first-layer adhesion problems to the build plate, insufficient exposure time causing poor layer bonding, and over-curing during post-processing that leads to brittleness and loss of fine detail. More information about printing failures can be found e.g. on Formlabs support page.

3 Dimension test plate for FDM printed parts

A dimension test plate seen in Figure 8 was designed to assess the accuracy and quality of FDM printed parts concerning print orientation and dimensional consistency.

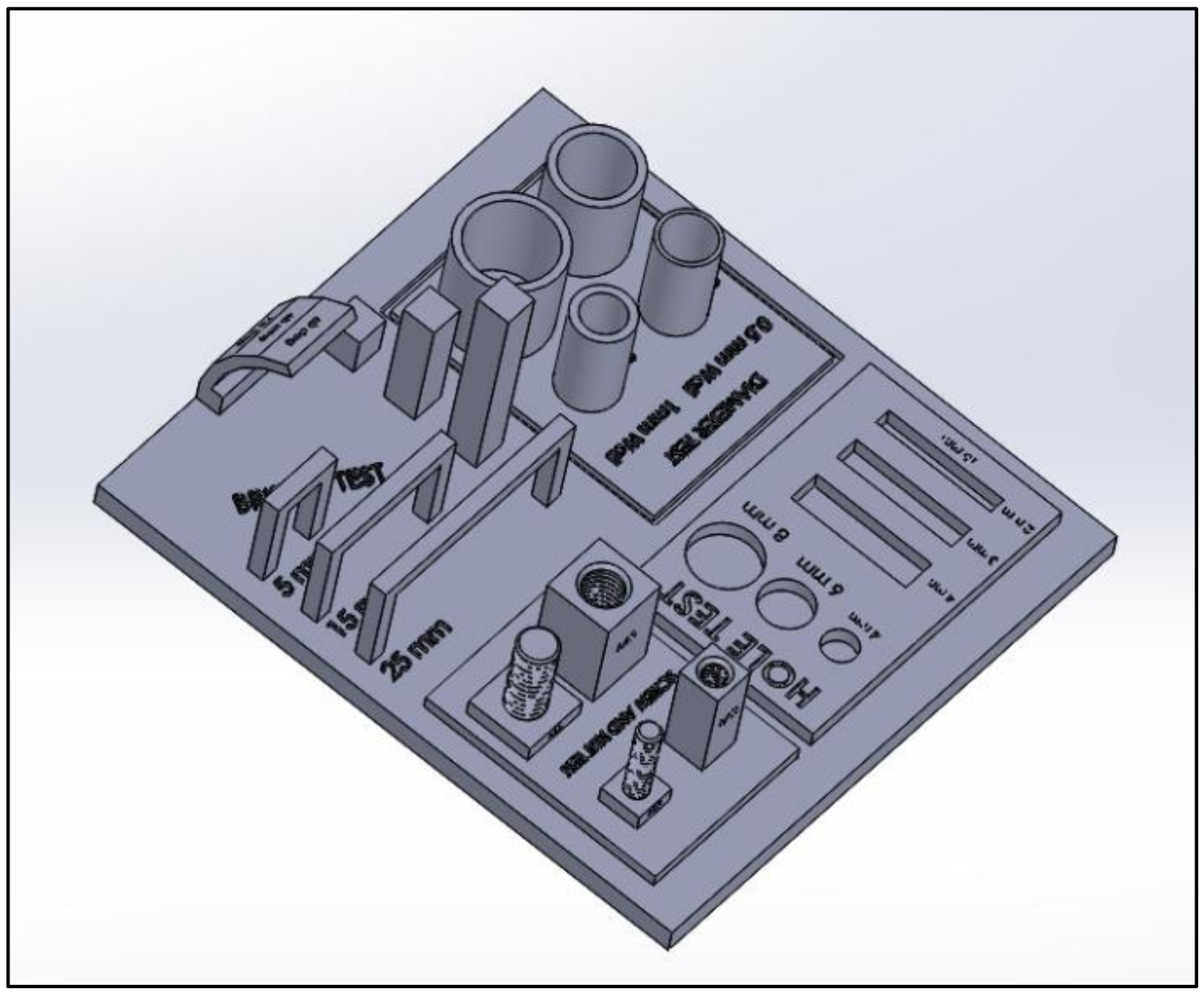

Supplementary Figure 8 CAD model of Dimension test plate for FDM printed parts with several critical elements including circular hole tests to evaluate the printer's ability to accurately reproduce internal geometries.

OSF - Storage – "Dimension_test_plate_FDM.STL" and "Dimension_test_plate_FDM.STEP"

4 Dimension test plate for SLA printed parts

A dimension test plate as shown in Figure 9 was designed to assess the printing quality and dimensional accuracy of SLA parts.

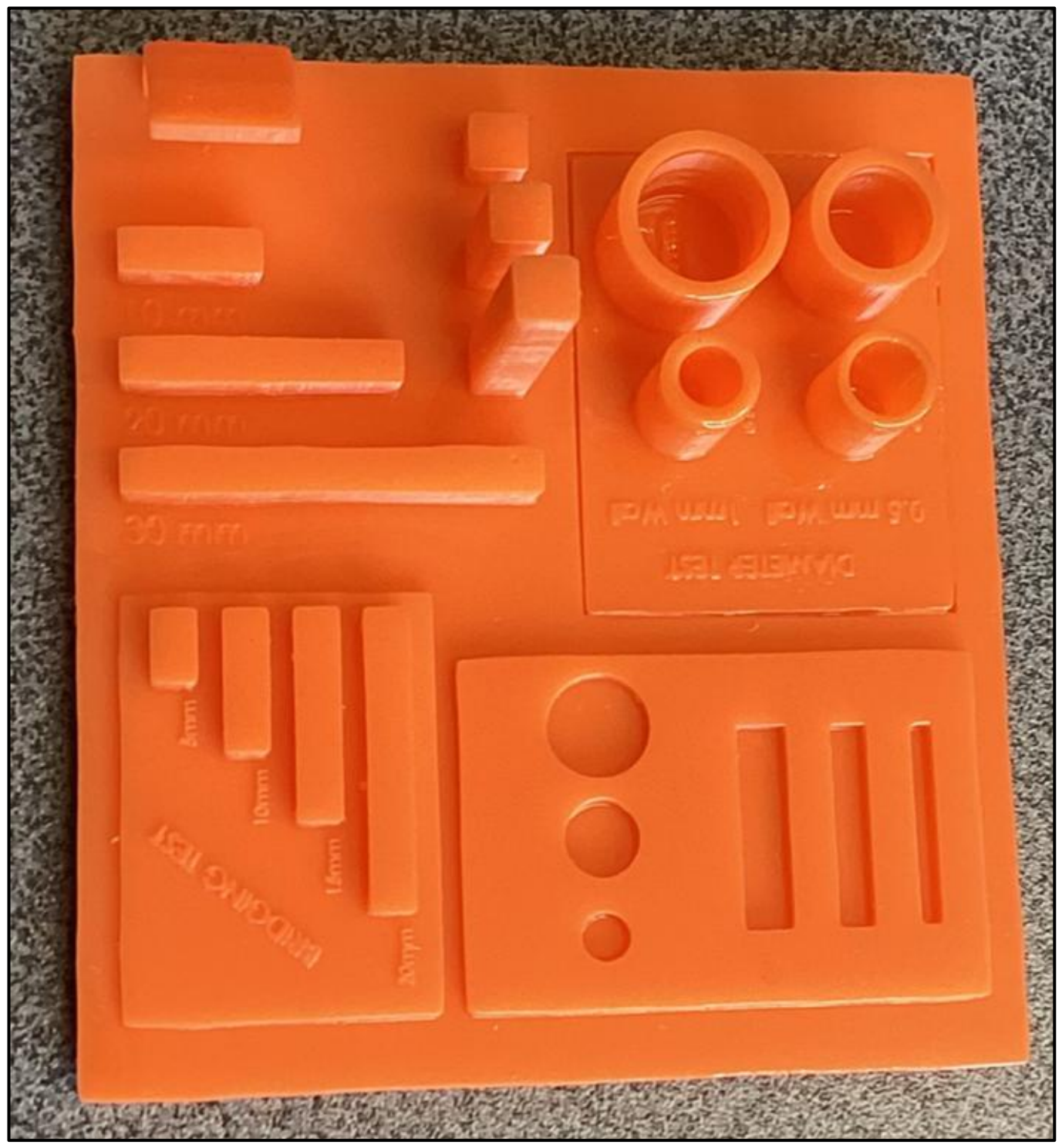

Supplementary Figure 9 Example of SLA-printed dimension test plate to assess the printing quality and dimensional accuracy.

OSF- Storage – “Dimension_test_plate_SLA.STL” and “Dimension_test_plate_SLA.STEP

5 Printing parameters of PLA filaments with their respective print parameters

Supplementary Table 1 List of PLA-based filaments with their respective print parameters

3D-printed brand	Nozzle Temperature	Bed Temperature	Printing Speed	Layer Height
Creality PLA HP Black	185 °C	First Layer: 65 °C<br>Other Layers: 60 °C	100 to 250 mm/s	First Layer: 0.35 mm<br>Layer Height: 0.30 mm
Elegoo PLA + Grey	205 °C	60 °C for all layers	100 to 200 mm/s	First Layer: 0.25 mm<br>Layer Height: 0.20 mm
eSun PLA + White	205 °C	50 °C for all layers	100 to 200 mm/s	First Layer: 0.30 mm<br>Layer Height: 0.25 mm

colorFabb PLA HP White	185 °C	60 °C for all layers	40 mm/s	First Layer: 0.35 mm<br>Layer Height: 0.30 mm
Verbatim PLA	210 °C	60 °C for all layers	100 to 300 mm/s	First Layer: 0.30 mm<br>Layer Height: 0.25 mm

Standard SLA print settings employed the 0.05 mm "Normal" system preset, with a layer height fixed at 0.05 mm and ten faded layers incorporated for optimal transition at the start of printing. Where support structures were required, parameters were set as follows: pinhead front diameter of 0.5 mm, head penetration of 0.5 mm, and pinhead width of 3 mm.

The printing parameters of the chosen photopolymer resins were found as follows:

Supplementary Table 2 Exposure times for the photopolymer resins used in this research

Photopolymer resin	Initial exposure time (s)	Exposure time (s)
SirayaTech Blu Tough Clear V2	24	3.3
Phrozen Protowhite Rigid	25	5
Prusament Tough Orange	25	4
SirayaTech Build Sonic Grey	28	4
SirayaTech Craft Ultra Clear	26	4
Liqcreate Bio Med Clear	35	4
Liqcreate Premium Tough	26*	8*
Liqcreate Tough X	50*	15*
Phrozen Aqua Snow Grey	25	2
Phrozen Aqua Hyperfine Graphite	28	4
eSun Standard Peach Pink	26	2.8
Elegoo plant based Tough Grey	28	3.5

Note: The asterisk (*) indicates the selection of a slow print speed feature on the Prusa SL1S Speed SLA printer. The specified values for initial exposure time and exposure time are based on a sample part measuring 30 × 30 mm x 3 mm. These parameters may change if the part size increases or if the quantity of parts per print is adjusted. It is recommended to re-calibrate exposure settings accordingly for larger prints or batch productions to maintain optimal print quality.

All materials follow the same post printing process for consistency of results which involved sequential washing in iso-propanol (IPA): an initial wash in IPA for 5 minutes, followed by a second wash in clean IPA for an additional 5 minutes.

Samples were then dried at 45 °C for 10 minutes before undergoing curing, which was performed for 10 minutes on each side to ensure full polymerization and material stability.

Material Exclusion:

ColorFabb PLA HP: Slow speed (40 mm/s), delamination despite nozzle (200–230°C), speed (+60 mm/s), heat bed (50–70°C) incremental change.

Liqcreate Tough X: Slow exposure (15/50 s @50 µm); layer (25–100 µm).

Liqcreate Premium Tough: Bonding/surface defects; unresolved by exposure (12–18 s), initial layer (40–60 s).

6 Design of test sample

- To calculate the extraction ratio (ER) (According to ISO 10993 – 12),
  - $ER = \frac{Surface\ Area\ in\ touch\ with\ medium}{V_{reaction}}$
  - $ER = \frac{2\pi rh + \pi r^2}{V_{reaction}}$ where h is the height till which the reactor vessel is filled up to & "r" is the radius of the vessel of the reactor vessel.
  - To find h ; => $\frac{V_{reactor}}{V_{reaction}} = \frac{100}{20}$
  - $\frac{\pi * 2^2 * 8}{\pi * 2^2 * h} = \frac{100}{20}$ ∴ h = 1.6 cm
  - $\therefore New\ ER = \frac{FOS * (2\pi rh + \pi r^2)}{20}$ where Factor of Safey (FOS) = 1.5 ; πr^2 because of lateral surface area of reactor and r = 2 cm & h = 1.6 cm.
  - ∴ New ER = 2.45 cm^2 / ml
- For the biocompatibility test check, we consider a test disc sample disc with diameter (D_t) as 45 mm and height of the test sample (H_t) as 2 mm with ER as 2.45 cm^2 / ml in reference to the bioreactor:
  - $\therefore ER = \frac{Surface\ Area\ of\ the\ test\ sample}{V_{medium}}$
  - $2.45\ cm^2\ /\ ml\ = \frac{2\pi r(r+h)}{V_{reaction}}$
  - V_{medium} = 14.1 ml where V_{medium} is the volume of medium needed to conduct the test per sample.

With multiple iterative trials dimension check, the new diameter (D_t) as 27 mm and height of the test sample (H_t) as 2 mm with the ER as 3 cm^2 / ml (ISO 10993-12) in reference to the bioreactor. The number of a test samples in one flask would be three in order to increase the surface area of the test sample in contact with the medium.

- $ER = \frac{Surface\ Area\ of\ the\ test\ sample}{V_{medium}}$
- $3\ cm^2\ /\ ml\ = \frac{3 * (2\pi r(r+h))}{V_{medium}}$ where r = 27/2 = 13.5 mm, h = 2 mm
- ∴ V_{medium} = 13.14 ml where ∴ V_{medium} is the volume of medium needed per test sample according to ISO 10993 -12.
- Therefore, New (D_t) = 27 mm, (H_t) = 2 mm, n = 3.

7 Hardness Test

a) OSF - Storage – “Dimension embarking plate.STL” & “Hardness test specimen.STL”

b) The testing utilized a Sauter Durometer HDD 100-1 (Sauter GmbH, Balingen, Germany), mounted on a Manual Shore test stand TI-DL (Sauter GmbH, Balingen, Germany), with a calibrated constant load of 50 N for all measurements. Hardness comparison plates AHBD-01 (KERN & SOHN GmbH, Balingen, Germany) were employed for reference to verify durometer calibration.

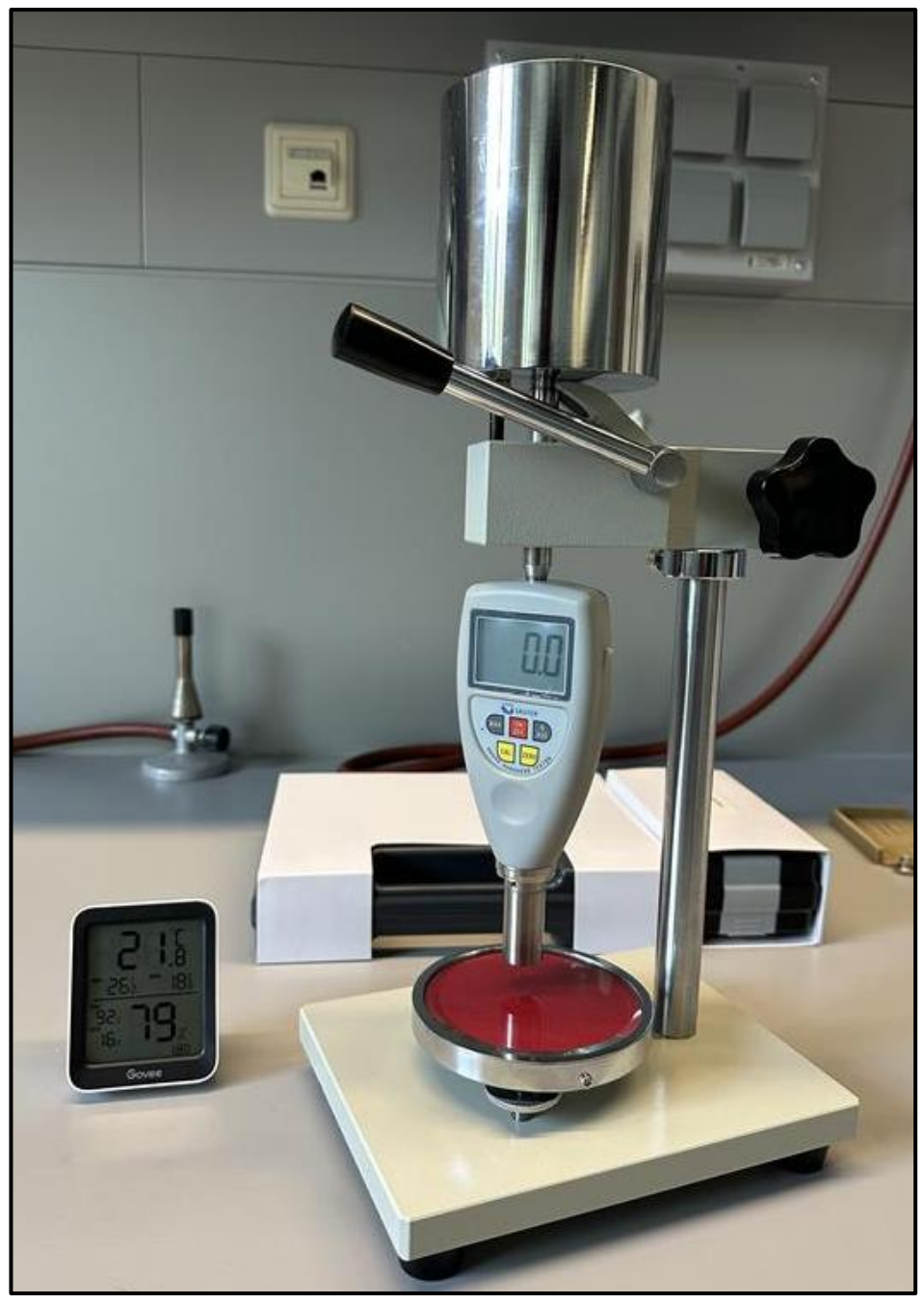

Supplementary Figure 10 Sauter shore D HDD 100-1 attached to manual test stand (Sauter test stand TI-DL) with 50 N constant force measurement

8 3-point bending test

a) OSF - Storage – “Bending Test Specimen.STL”

b) Figure 6 shown below shows the test setup and conditions for three-point bending test

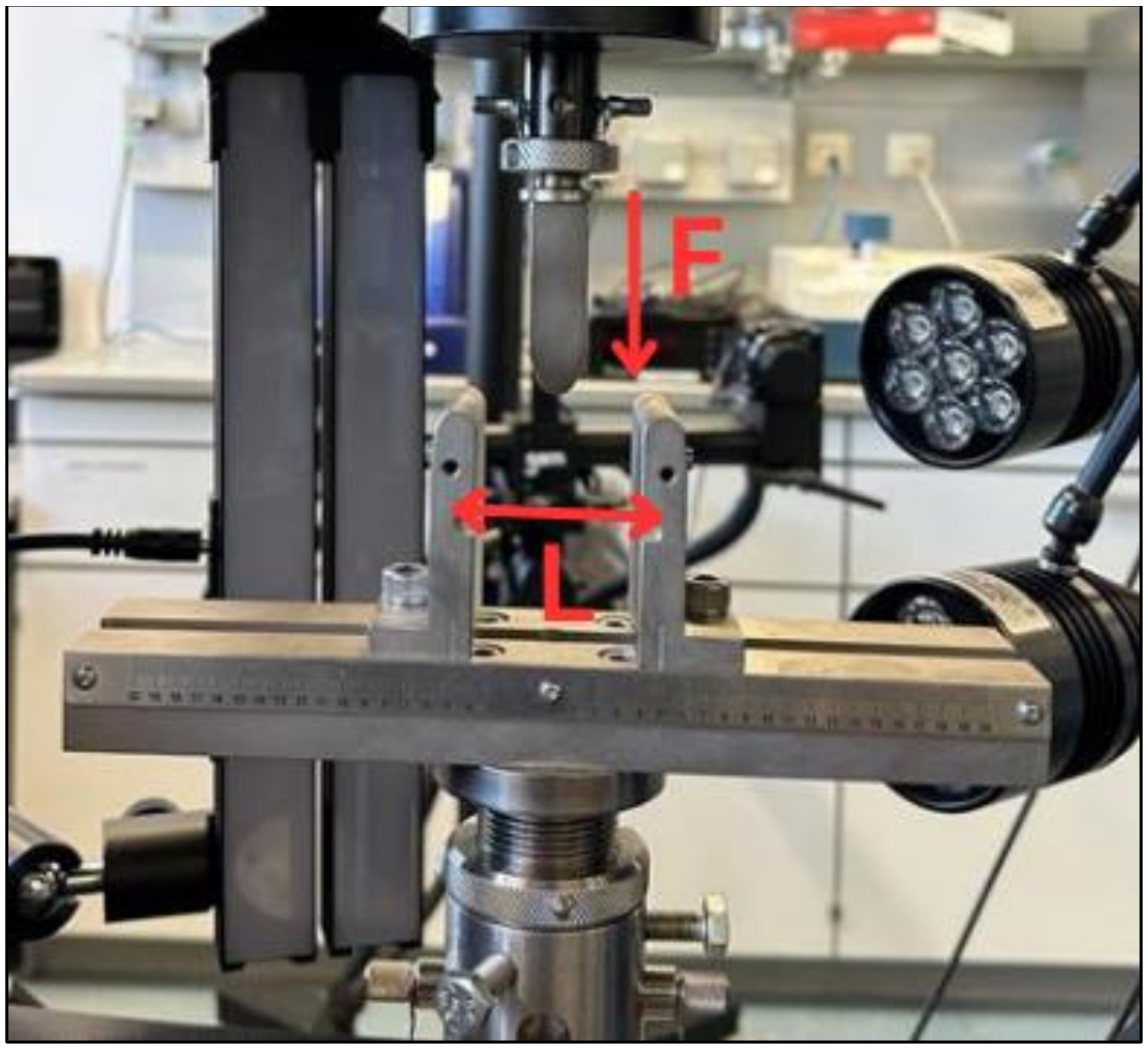

Supplementary Figure 11 3-point bending test setup where "F" as the applied force and "L" is the support span length

c) Bending test results:

Supplementary Table 3 Average flexural deflection and flexural strength of PLA filaments before and after autoclave.

Material	Deflection before autoclave (mm)	Deflection after autoclave (mm)	Flexural Strength before autoclave (MPa)	Flexural Strength after autoclave (MPa)
Creality PLA HP Black	4.9 ± 0.1	6.8 ± 0.4	88.9 ± 0.6	101.9 ± 2.5
Elegoo PLA+ Grey	4.3 ± 0.1	4.6 ± 0.2	81.0 ± 0.5	81.7 ± 2.3
eSun PLA+ White	4.7 ± 0.1	4.8 ± 0.6	93.7 ± 1.5	95.6 ± 4.2
Verbatim PLA	4.9 ± 0.1	4.8 ± 0.3	102.6 ± 0.9	117.2 ± 2.3

Supplementary Table 4 Average flexural deflection and flexural strength of photopolymers before and after autoclave.

Material	Deflection before autoclave (mm)	Deflection after autoclave (mm)	Flexural Strength before autoclave (MPa)	Flexural Strength after autoclave (MPa)
Prusament Tough Orange	5.2 ± 0.5	3.7 ± 0.9	51.0 ± 2.7	34.4 ± 3.6
SirayaTech Tough Blu V2	7.6 ± 0.3	8.6 ± 0.1	74.7 ± 2.9	44.7 ± 2.6
Liqcreate Bio Med Clear	6.0 ± 0.8	7.3 ± 0.2	108.1 ± 1.7	96.0 ± 1.3
eSun Standard Peach Pink	4.1 ± 0.3	3.4 ± 0.4	103.9 ± 3.1	92.8 ± 4.5
Elegoo plant-based Tough Grey	6.2 ± 0.5	5.1 ± 1.4	98.2 ± 4.4	84.2 ± 5.9

9 K. marxianus Mineral Medium Recipe

Supplementary Table 5 Glucose (10× stock solution, 20 g/L) – 500 mL, to obtain a final concentration of 2 g/L

Component	Amount added
Glucose.H_2O	11 g

Supplementary Table 6 Macro Elements (Löser et al., 2021) (1000 mL)

Component	Amount added
$(NH_4)_2SO_4$	5 g
KH_2PO_4	3 g
$MgSO_4$ * 7 H_2O	0,5 g
NaCl	0,1 g

$CaCl_2$ * 2 H_2O – stock solution (0,1 g/L)	10 mL (c = 0,001 g/L)
MOPS (Puffer)	20.926 g
Citrat * H_2O	1,04 g

-> dissolve in 500 mL deionized H2O

-> adjust pH to 7 with 5M KOH / NaOH

-> fill up to 900 ml

-> autoclave (20-25 min, 121 °C)

-> add Glucose solution (volume depending on stock solution) – 100 ml.

+ 0,5 mL (0,625 mL) Trace element solution

+ 2 mL (2,5 mL) Vitamin solution

+ fill up to 1 L with deionized H2O

Supplementary Table 7 Trace Elements (Urit et al., 2012) (1000 mL)

**Component**	**Amount added**
$FeSO_4$ * 7 H_2O	25 g
$ZnSO_4$ * 7 H_2O	25 g
$CuSO_4$ * 5 H_2O	5 g
$MnSO_4$ * 4 H_2O	5 g
$CoSO_4$ * 7 H_2O	1 g
H_3BO_3	1 g
Na_2MoO_4 * 2 H_2O	0,5 g
$NiSO_4$ * 6 H_2O	0,5 g

KI	0,5 g

-> dissolve in 500 mL deionized H_2O

-> add 300 mL 1 N HCl

-> fill up to 1 L

-> autoclave (121 °C, 20 min)

Supplementary Table 8 Vitamins (Löser et al., 2021) (1000mL)

**Component**	**Amount added**
Inositol	12,5 g
-> dissolve in 200 mL of 20 mM NaOH	
Calcium pantothenate	500 mg
Nicotinic acid	500 mg
Thiamine * HCl	500 mg
Pyridoxine * HCl	500 mg
4-Aminobenzoate	100 mg
Riboflavin	100 mg
Folic acid	100 mg
Biotin	25 mg
-> dissolve each in 100 mL 20 mM NaOH	

-> combine with 1 L Vitamin solution and sterile filtrate

Löser, C., Kupsch, C., Walther, T., & Hoffmann, A. (2021). A new approach for balancing the microbial synthesis of ethyl acetate and other volatile metabolites during aerobic bioreactor cultivations. Engineering in Life Sciences, 21(3–4), 137–153. https://doi.org/10.1002/ELSC.202000047

Urit, T., Stukert, A., Bley, T., & Löser, C. (2012). Formation of ethyl acetate by Kluyveromyces marxianus on whey during aerobic batch cultivation at specific trace element limitation. Applied Microbiology and Biotechnology, 96(5), 1313–1323. https://doi.org/10.1007/S00253-012-4107-Z/TABLES/2

10 Preparation of agar plates with LB medium

Step 1: Agar medium: 15g Agar Agar/L (Agar-Agar, Bioscience, Carl Roth GmbH + Co. KG, Karlsruhe, Germany) & 25g LB medium/L [LB-Medium(Luria/Miller), Carl Roth GmbH + Co. KG, Karlsruhe, Germany]

--> preparation time ~15 min.

Step 2 : Fill in the fitting flask with distilled H2O, stir, put in autoclave (open lid), heat up to 121 °C for 20 minutes.

Step 3 : While the autoclave is running, heat up the water bath to 60 °C. When the Agar-LB mixture is done, take your flask from the autoclave to the water bath and store it there until use (in our case we will directly use the liquid after autoclave so no need for water bath). Autoclaving process takes about ~ 2 to 2h 30 min.

Step 4 : Prep your plates under the sterile bench , label as "LB" on the bottom of the plates. Add your desired antibiotic to the flask under the sterile bench (or add nothing if you just want blank LB plates - in our case we won't add any antibiotics).

Step 5 : Pour medium in plates, keep lid off, wait ~5-15 min depending on the thickness of your plates, then close the lid on each plate and store them at 4 °C in a plastic bag (preparation time ~45 min).

11 Inoculation of main culture calculation:

The inoculum volume required for each main culture was calculated based on the following relations:

$OD_{pre\text{-}culture} * V_{pre\text{-}culture} = OD_{main\ culture} * V_{main\ culture}$

where :

$OD_{pre\text{-}culture}$ = measured OD of pre-culture 2

$V_{pre\text{-}culture}$ = volume of pre-culture 2 to be added (µL)

$OD_{main\ culture}$ = approximate starting OD of main culture (0.1)

$V_{main\ culture}$ = total volume OD of main culture (10 ml per flask)

Using this calculation, the appropriate inoculum volume of pre-culture 2 was added to each 10 mL eluate-containing Erlenmeyer shake flask (experimental, positive, and negative controls alike). All shake flasks were incubated under standardized conditions at 37 °C and 220 rpm in an orbital shaking incubator.

12 S-Gompertz Model

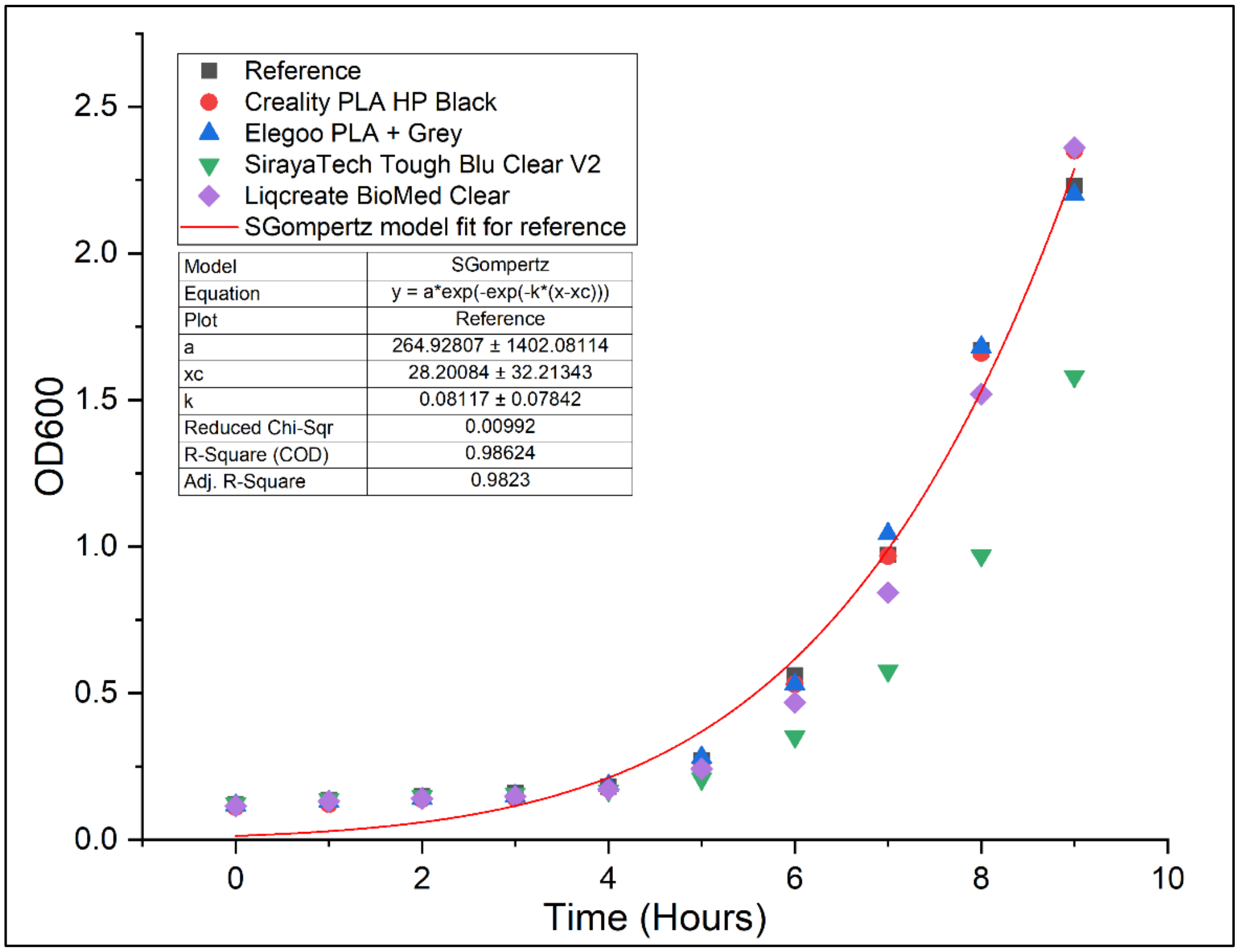

Supplementary Figure 12 Gompertz-fitted growth curves for PLA filaments and photopolymer resins vs. reference.

The SGompertz model was fitted with the values obtained from the reference sample. In biological and growth contexts like microbial proliferation or population dynamics, these parameters represent:
a: Maximum population size or carrying capacity; the upper limit the biological system (e.g., cell count or OD).
xc: Time or age at maximum growth rate.
k: growth rate.

13 SeCaT-meter 600

Supplementary Table 9 OD Measurements of cultivation of yeast in glass and Creality PLA HP made 3D-printed Shake Flasks Over Time

Time	Glass shake flask OD (Genesys150)	Creality PLA HP 3D-printed shake flask (Genesys150)	Glass shake flask OD (SeCaT-meter 600)	Creality PLA HP 3D-printed shake flask (SeCaT-meter 600)
**8 h**	0.210<br>0.210	0.231<br>0.231	0.211<br>0.211	0.225<br>0.224

	0.209	0.232	0.212	0.225
**9h 20 min**	0.232<br>0.230<br>0.234	0.252<br>0.253<br>0.253	0.228<br>0.228<br>0.229	0.254<br>0.255<br>0.256
**10 h 30 min**	0.273<br>0.272<br>0.272	0.294<br>0293<br>0.292	0.272<br>0.272<br>0.272	0.293<br>0.292<br>0.290
**11 h 30 min**	0.345<br>0.343<br>0.346	0.376<br>0.381<br>0.382	0.344<br>0.348<br>0.348	0.373<br>0.376<br>0.374

14 OSF – Storage – "Conical Flask 100ml.STL"

15 Test sample images before and after autoclave

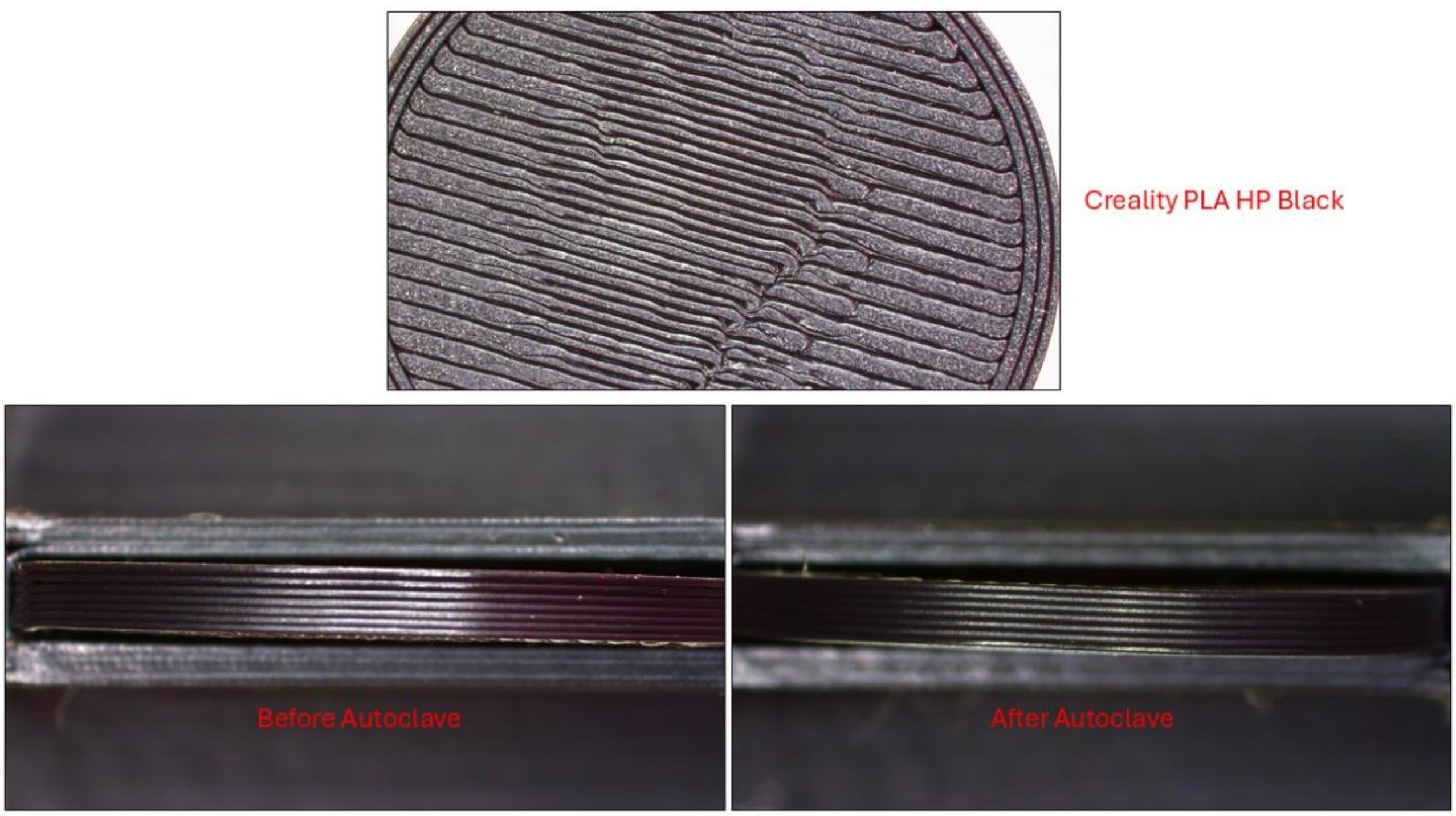

Supplementary Figure 13 Test sample of Creality PLA HP Black before and autoclave (no crack).

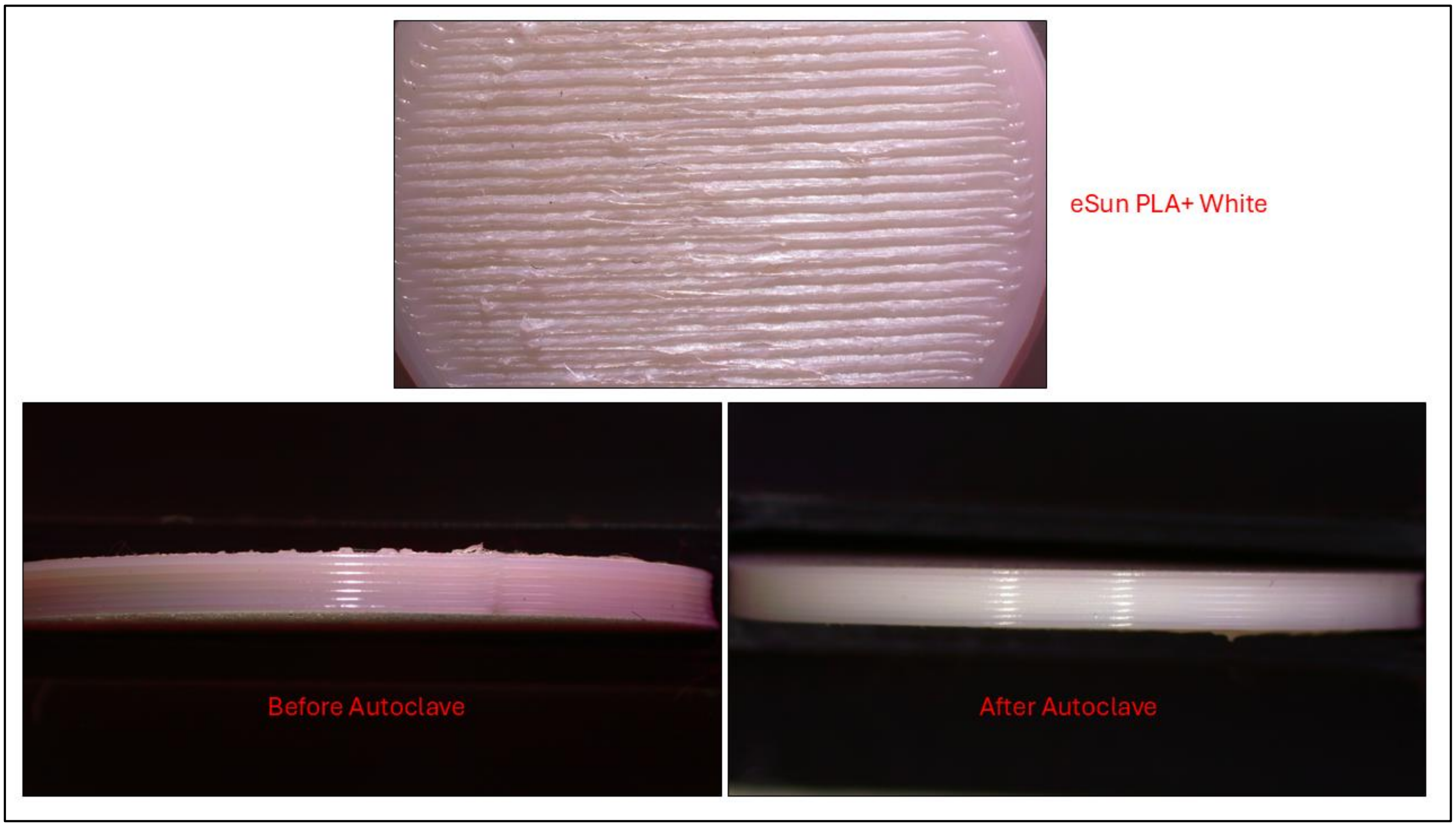

Supplementary Figure 14 Test sample of eSun PLA+ White before and autoclave (no crack).

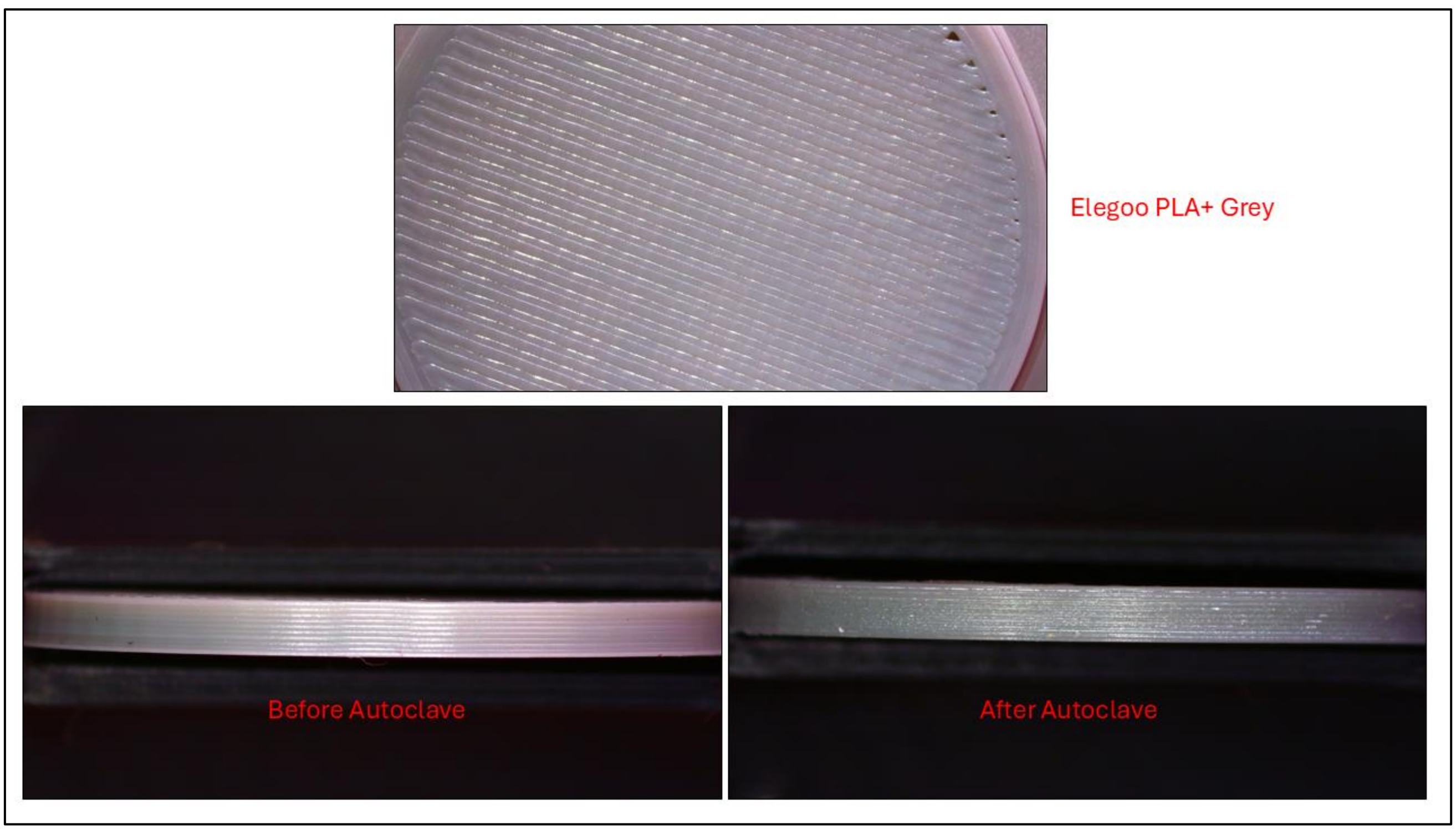

Supplementary Figure 15 Test sample of Elegoo PLA+ Grey before and autoclave (no crack).

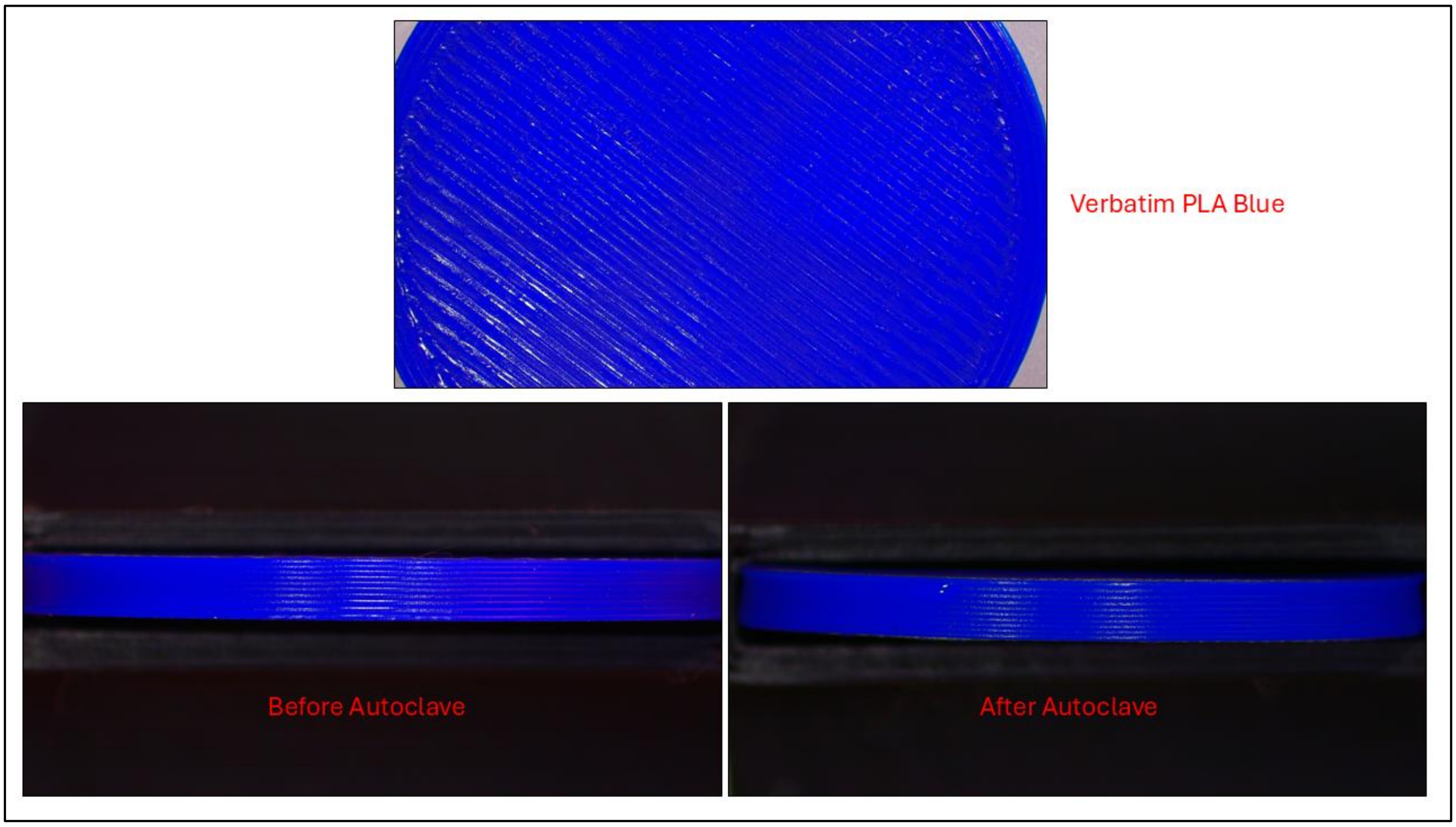

Supplementary Figure 16 Test sample of Verbatim PLA Blue before and autoclave (no crack).

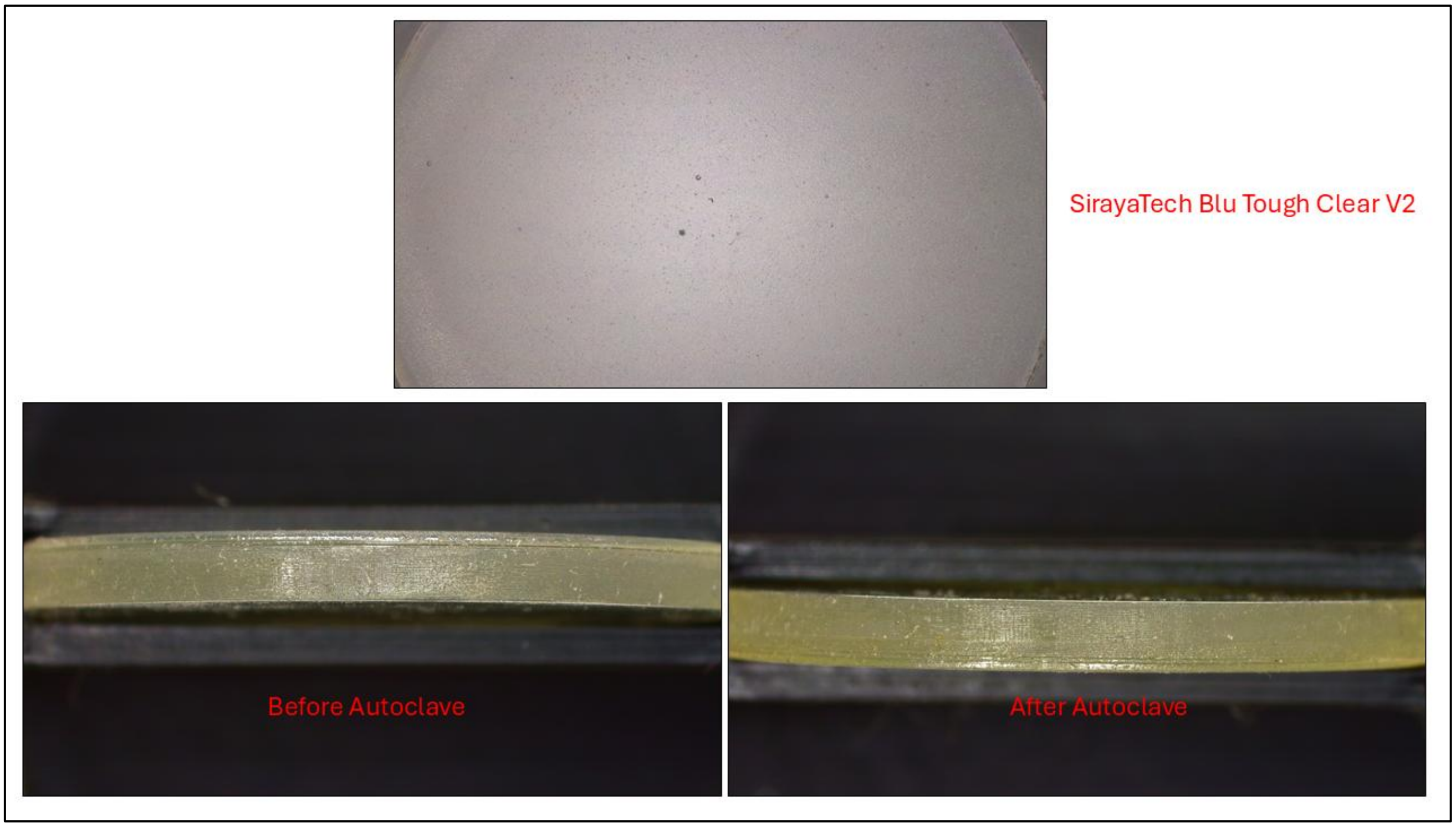

Supplementary Figure 17 Test sample of SirayaTech Blu Tough Clear V2 before and autoclave (no crack).

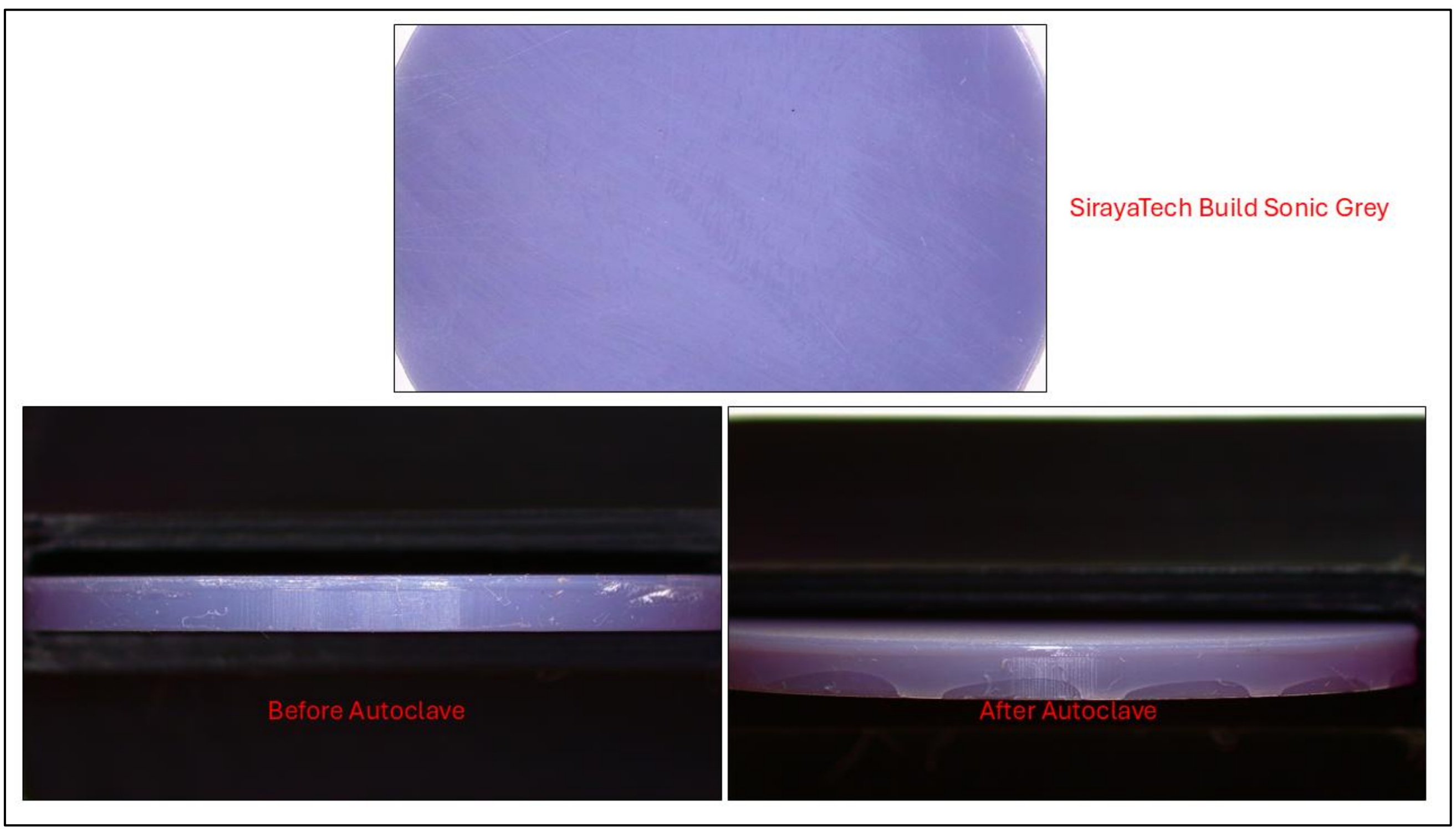

Supplementary Figure 18 Test sample of SirayaTech Build Sonic Grey before and autoclave (crack along layer lines).

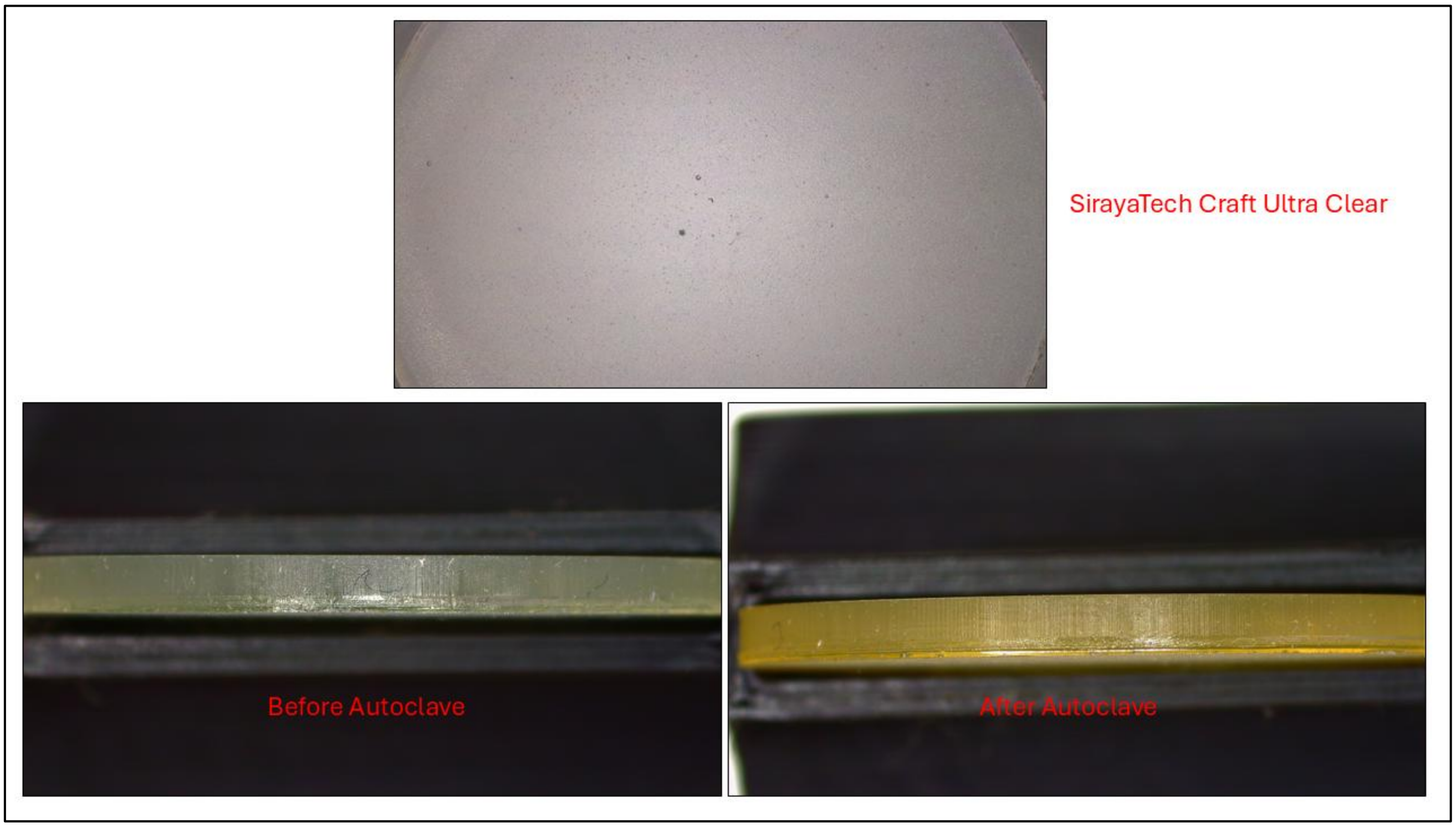

Supplementary Figure 19 Test sample of SirayaTech Craft Ultra Clear before and autoclave (crack along layer lines).

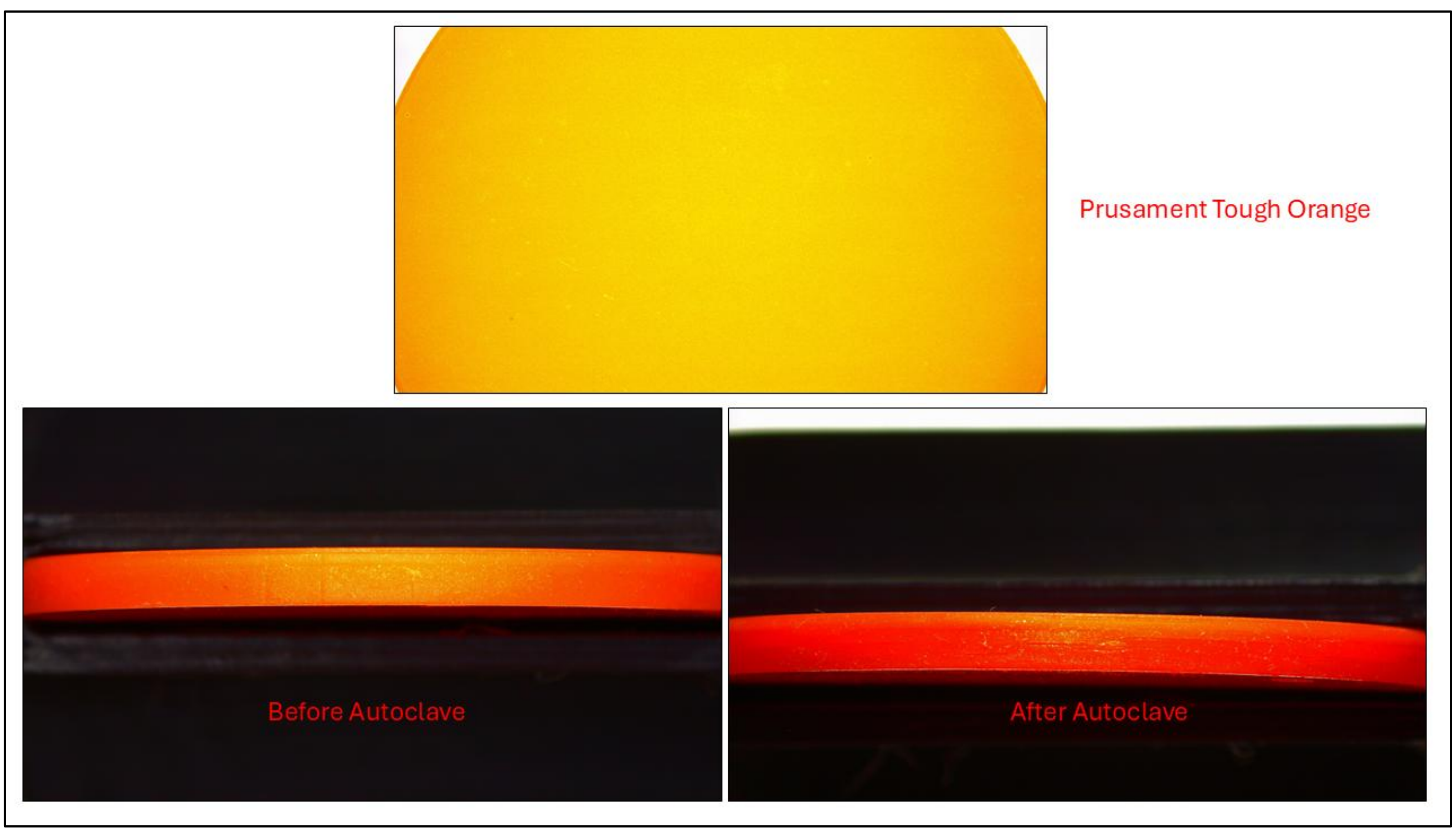

Supplementary Figure 20 Test sample of Prusament Tough Orange before and autoclave (no crack).

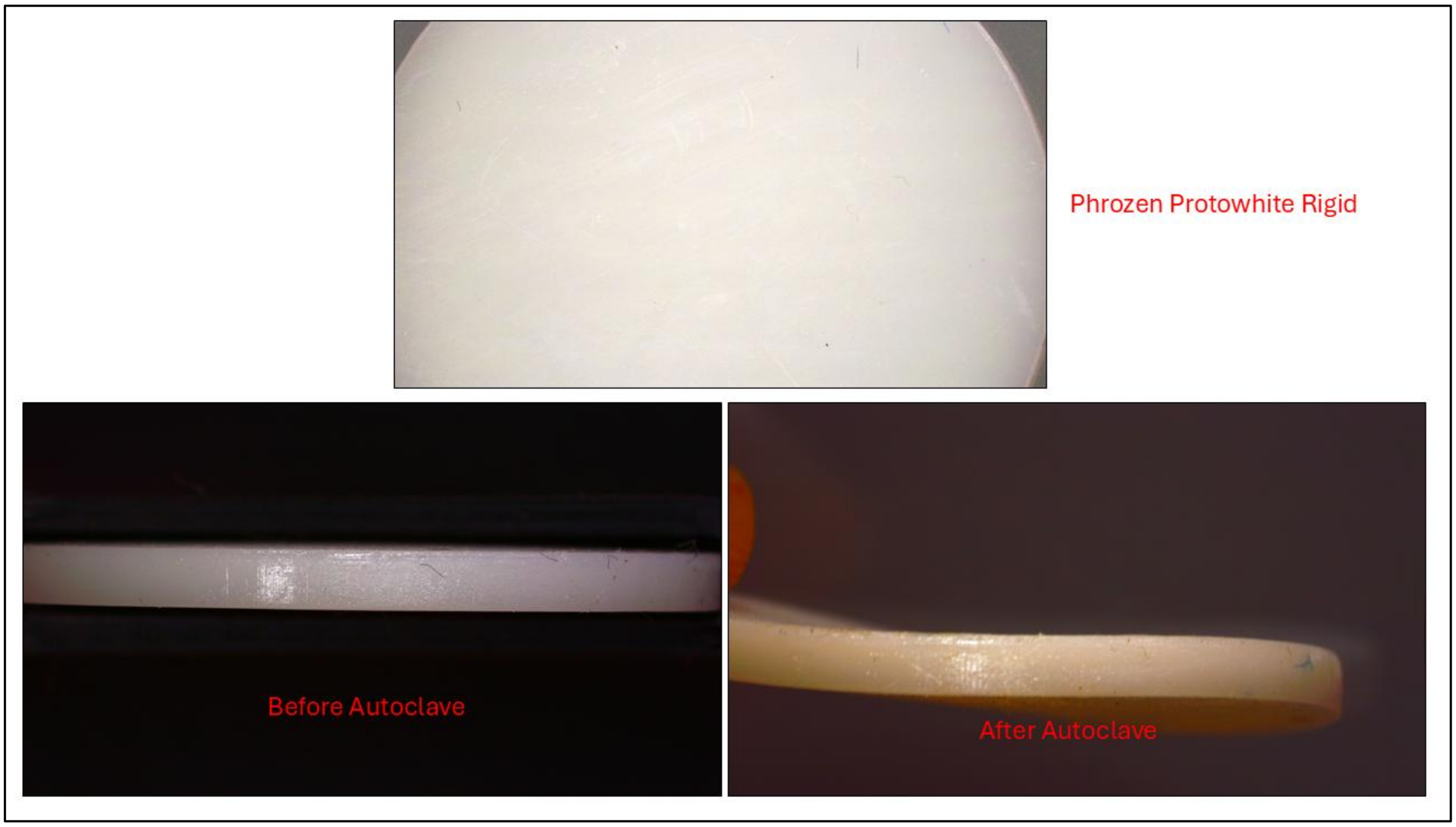

Supplementary Figure 21 Test sample of Phrozen ProtoWhite Rigid before and autoclave (bent)

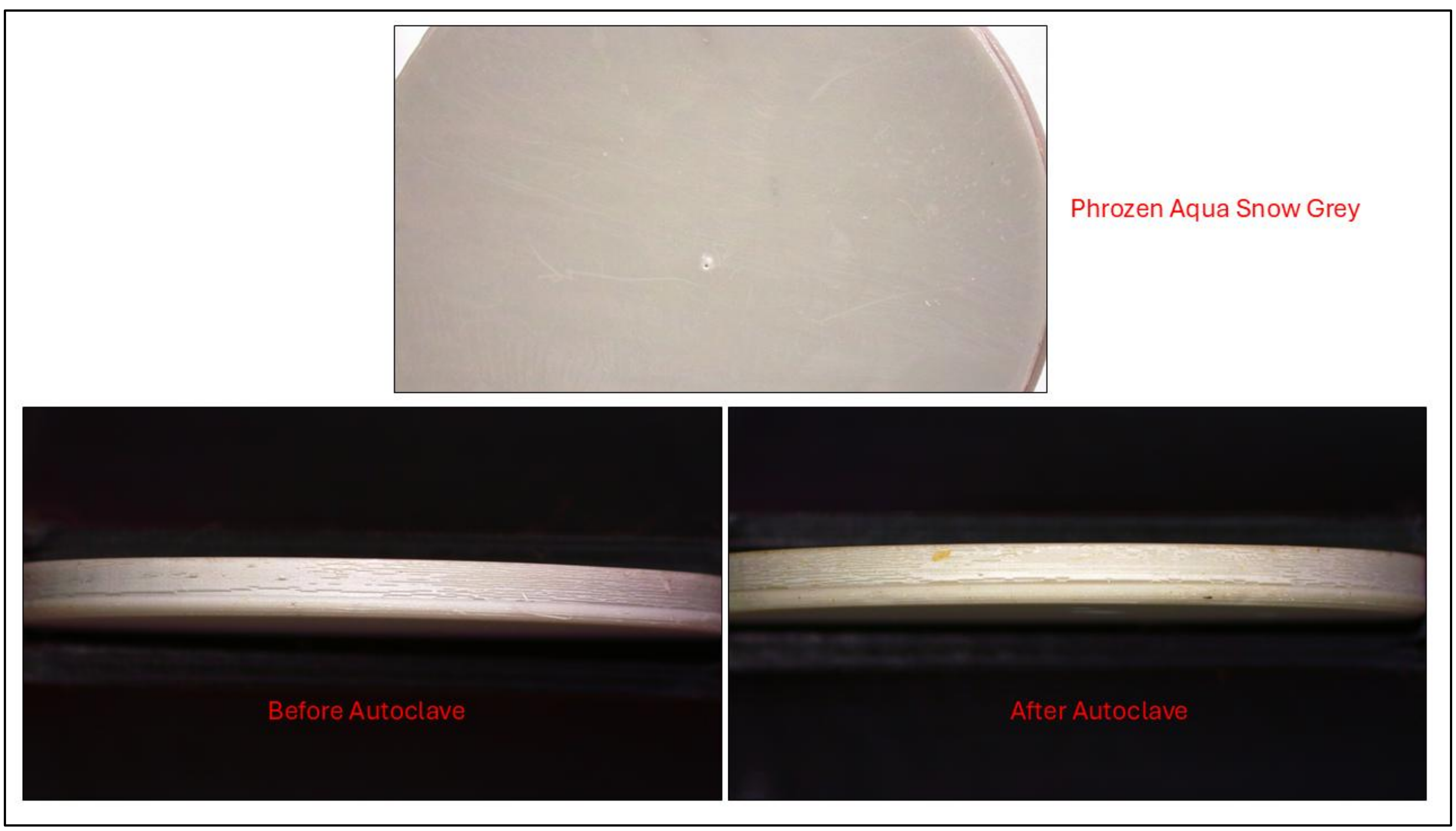

Supplementary Figure 22 Test sample of Phrozen Aqua Snow Grey before and autoclave (crack along layer lines)

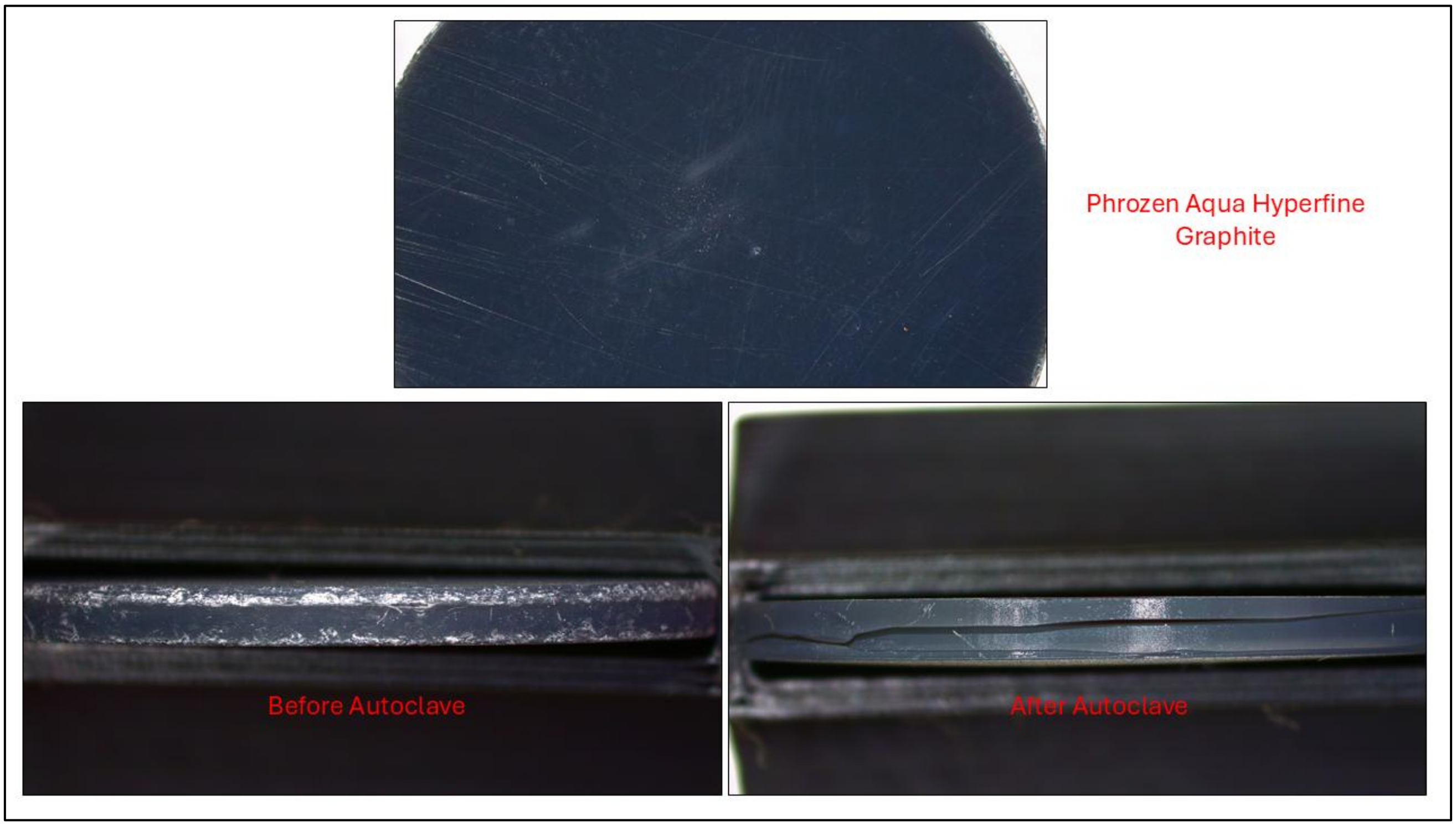

Supplementary Figure 23 Test sample of Phrozen Aqua Hyperfine Graphite before and autoclave (crack along layer lines)

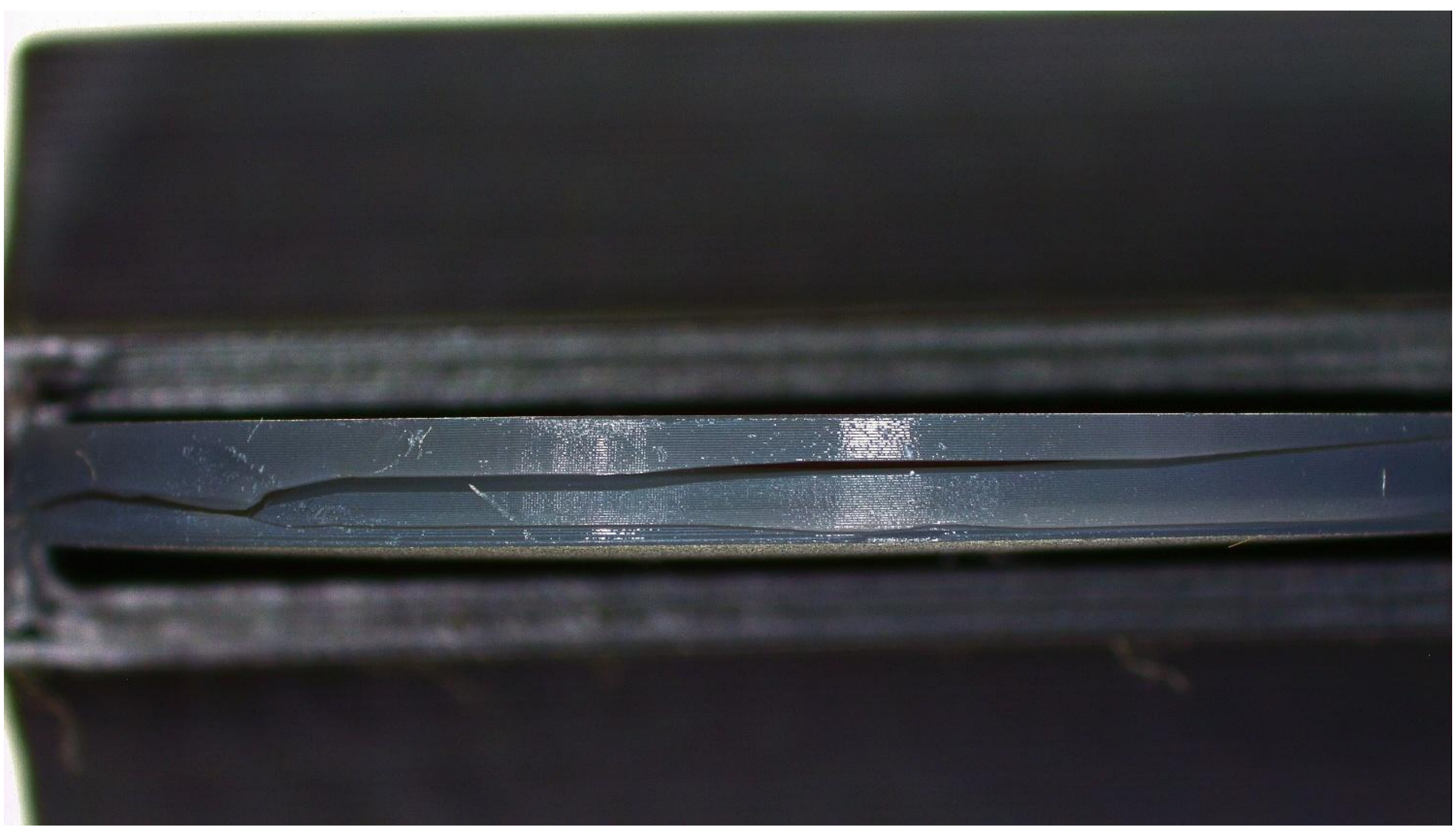

Supplementary Figure 24 Test sample of Phrozen Aqua Hyperfine Graphite (10 minutes of curing) before and autoclave (crack along layer lines)

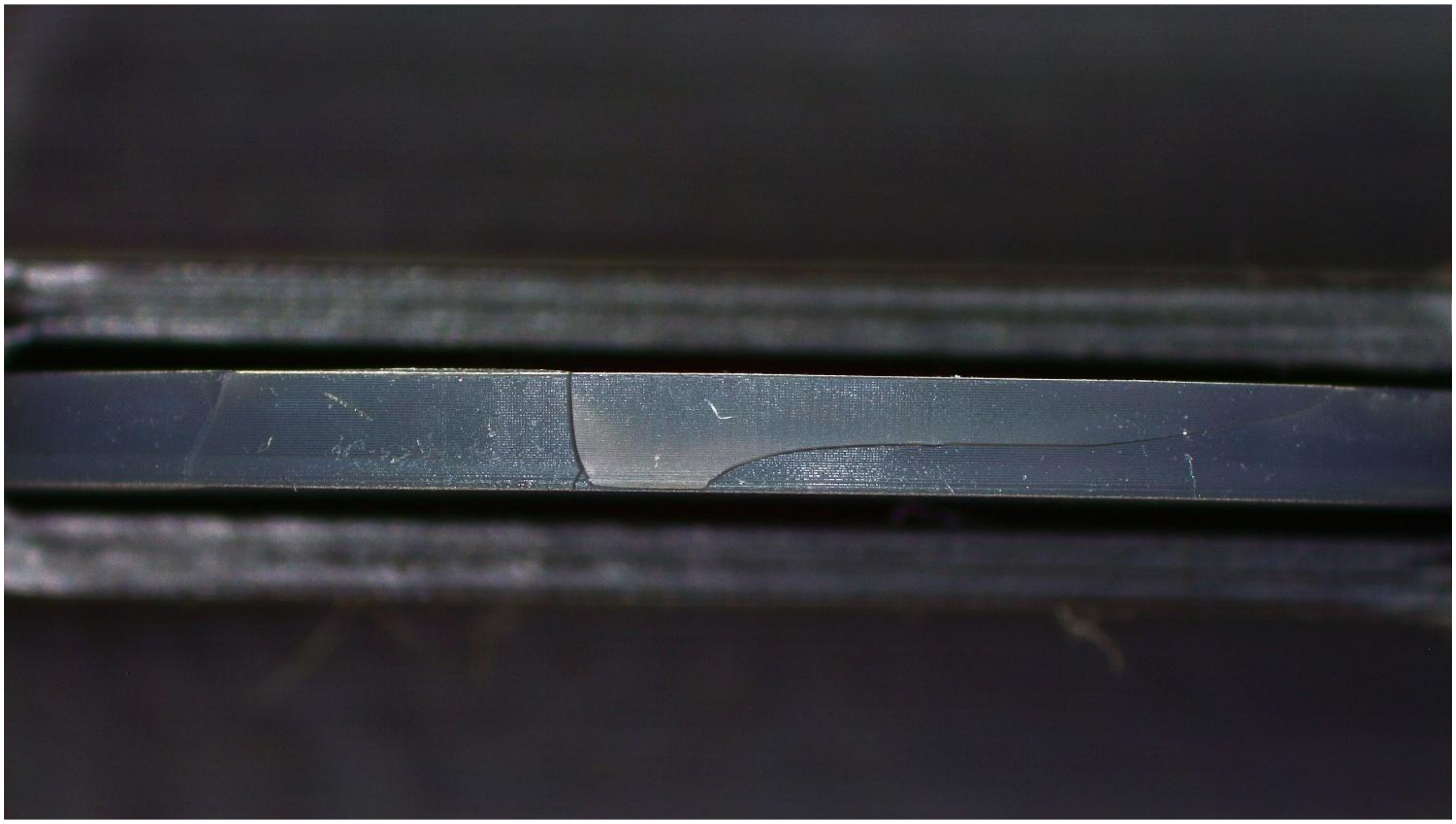

Supplementary Figure 25 Test sample of Phrozen Aqua Hyperfine Graphite (20 minutes of curing) before and autoclave (crack along layer lines)

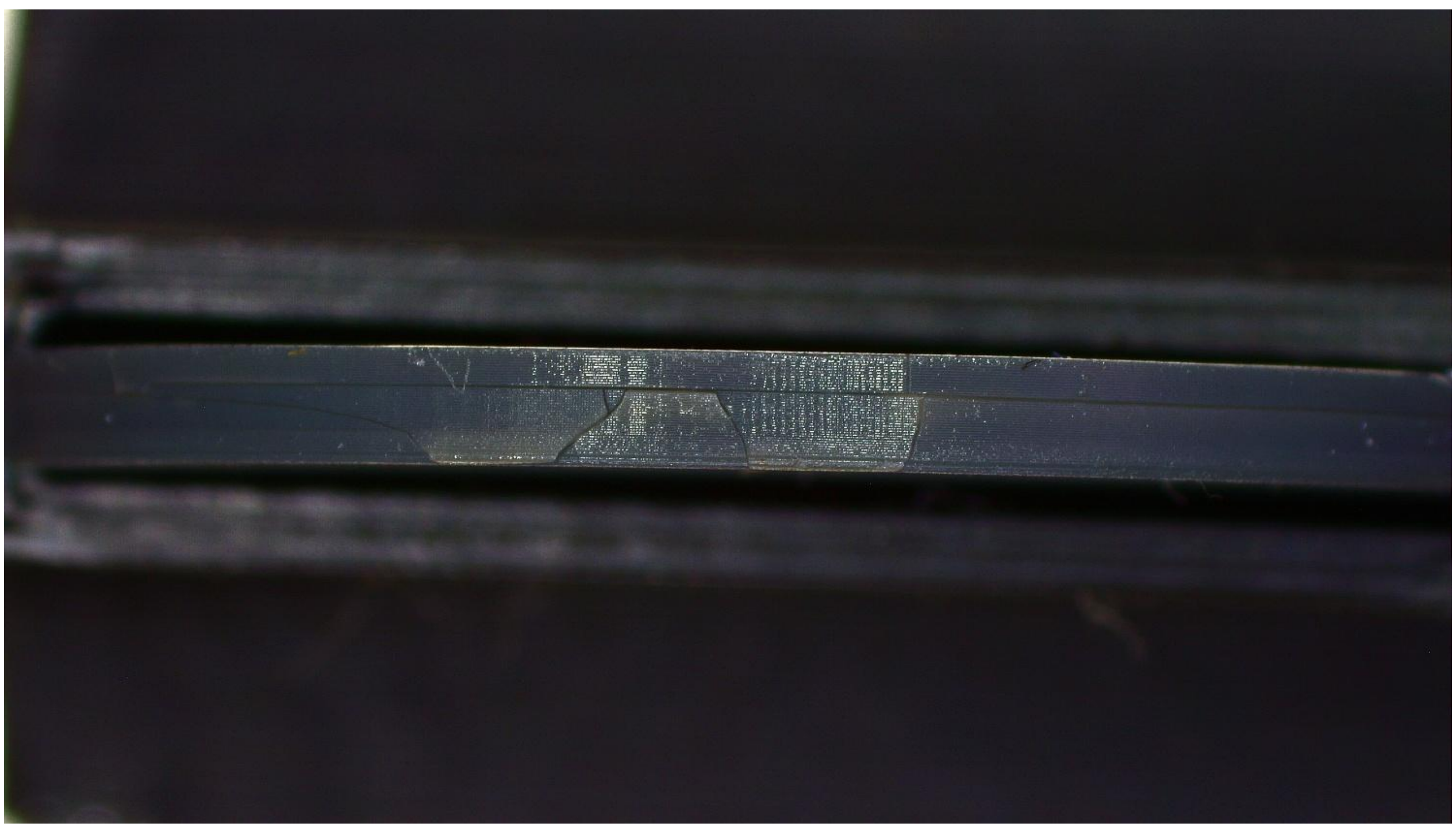

Supplementary Figure 26 Test sample of Phrozen Aqua Hyperfine Graphite (30 minutes of curing) before and autoclave (crack along layer lines)

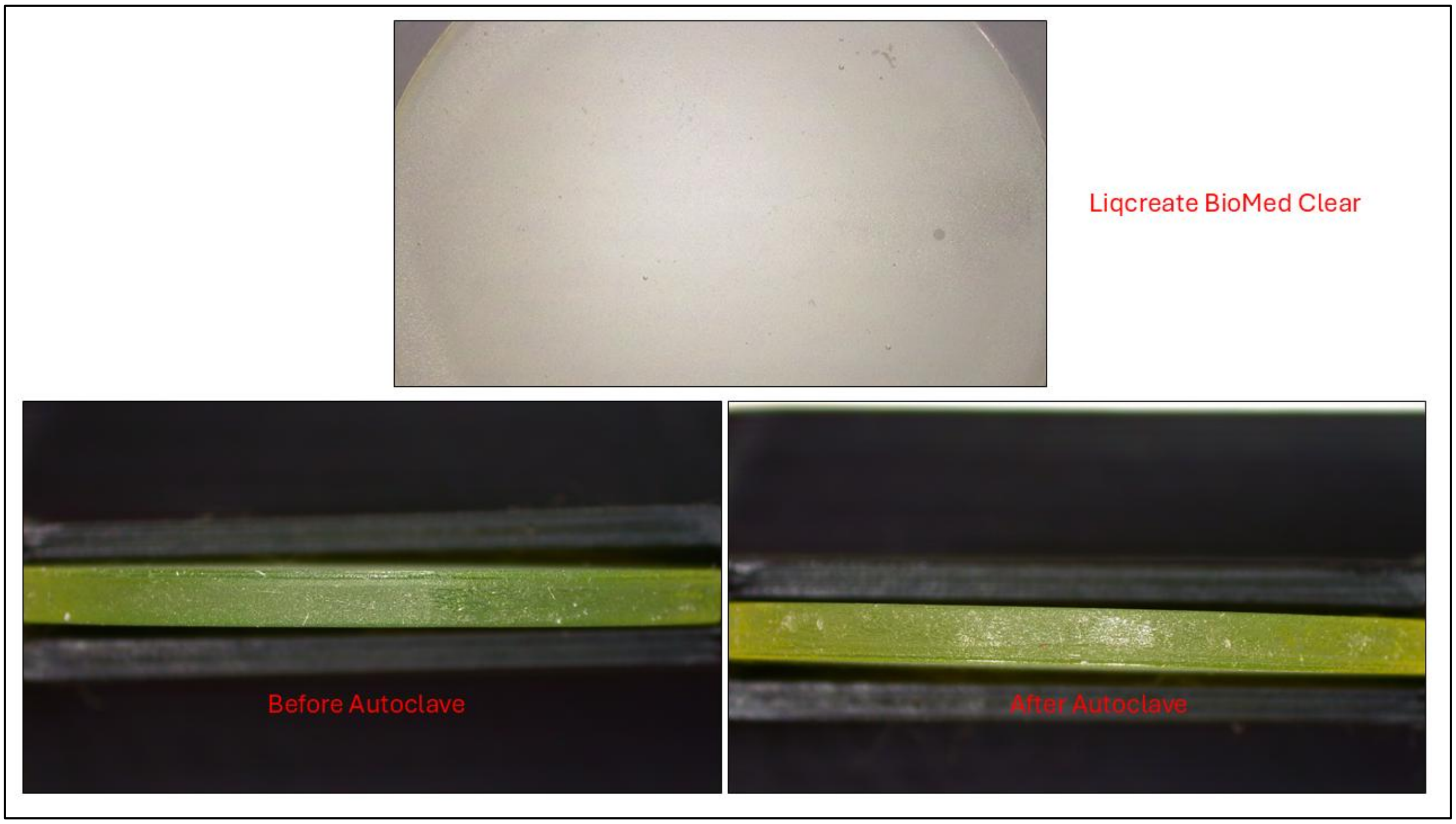

Supplementary Figure 27 Test sample of Liqcreate BioMed Clear before and autoclave (no crack)

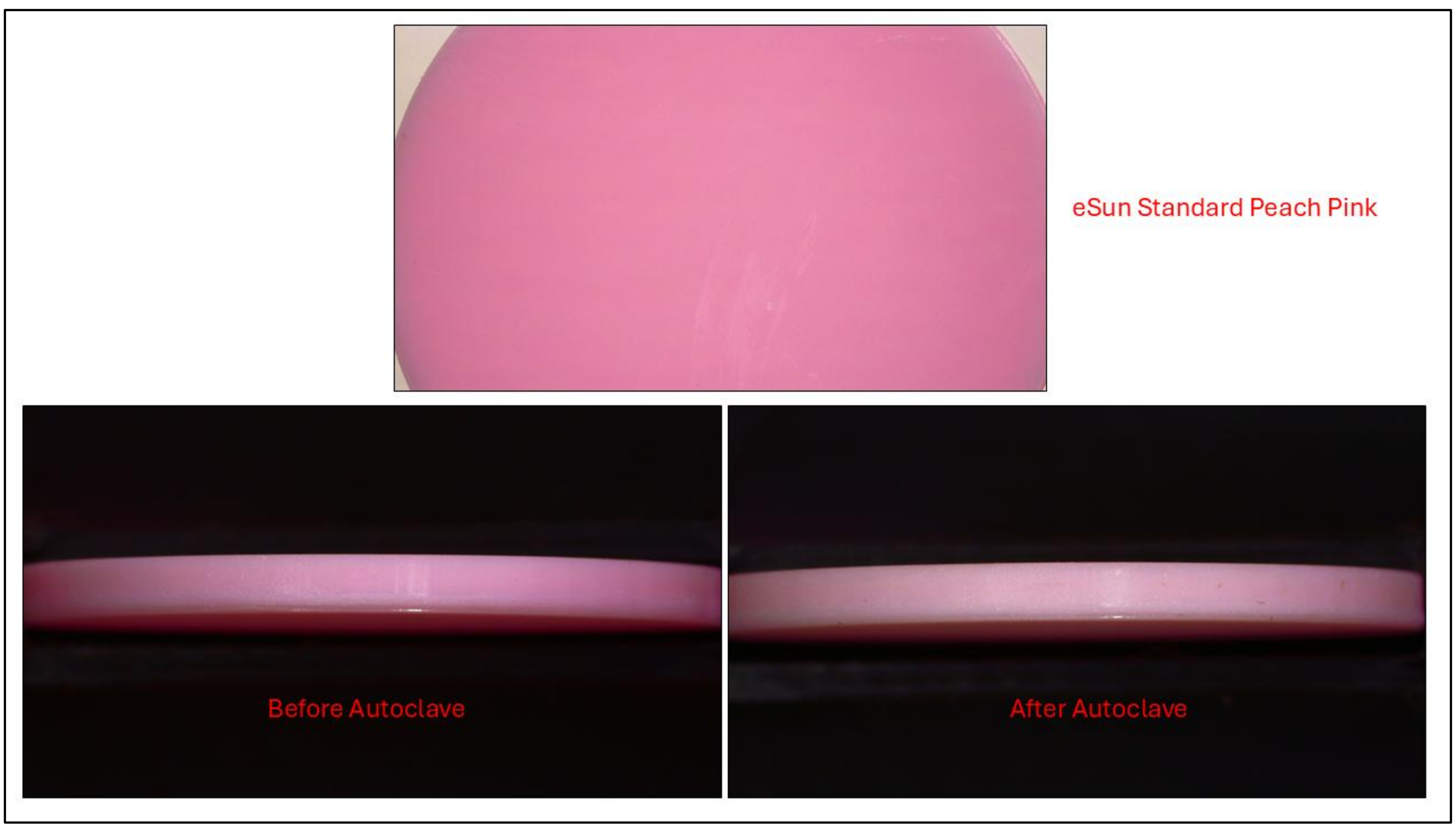

Supplementary Figure 28 Test sample of eSun Standard Peach Pink before and autoclave (no crack)

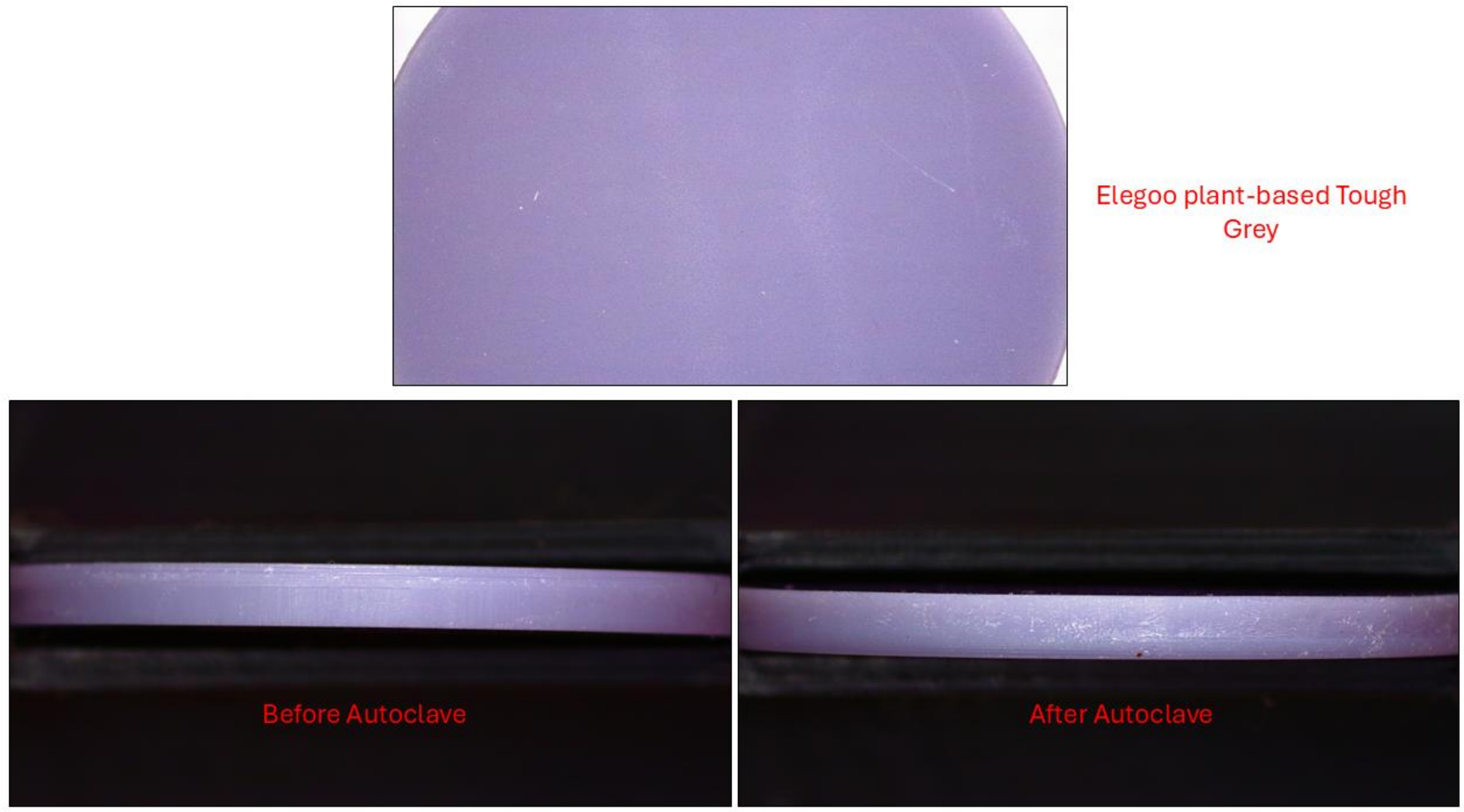

Supplementary Figure 29 Test sample of Elegoo plant-based Tough Grey before and autoclave (no crack)

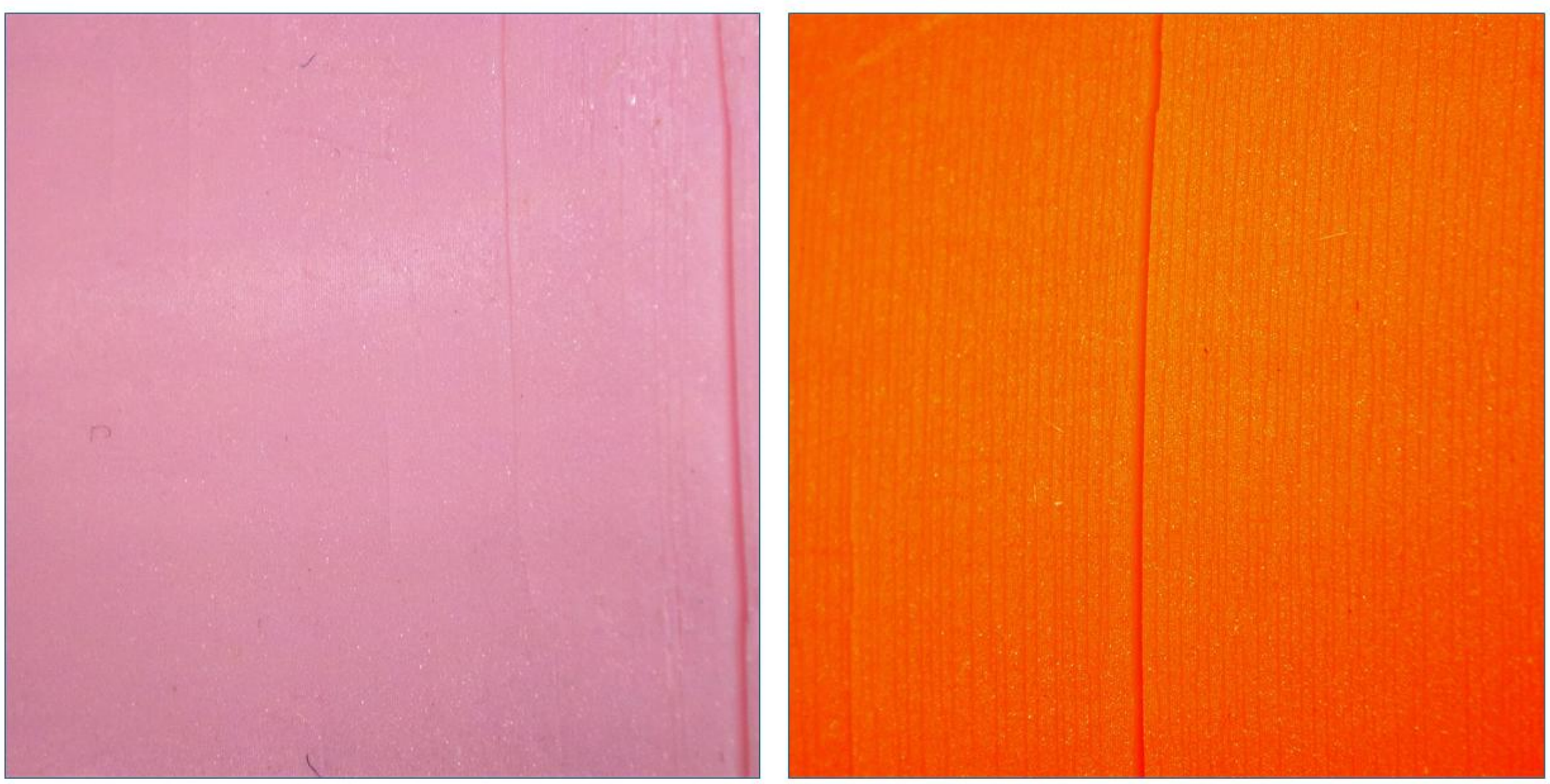

eSun Standard Peach Pink printed shake flask after 2nd autoclave cycle

Prusament Tough Orange shake flask after 2nd autoclave cycle

Supplementary Figure 30 Cracks observed at the outer wall of 3D-printed shake from eSun Standard Peach Pink (left) and Prusament Tough Orange (right)

16 Sensitivity analysis of sample sets with different curing times

Test Sample	Biocompatibility w.r.t reference	Shore D hardness test (Shore D)	3-point bending test (MPa)
eSun Standard Peach Pink 10 mins cured	0.91 ± 0.008	87.06 ± 0.582	94.68 ± 4.938
eSun Standard Peach Pink 20 mins cured	0.94 ± 0.019	89.12 ± 0.624	88.63 ± 11.192
eSun Standard Peach Pink 30 mins cured	0.99 ± 0.049	89.96 ± 0.637	97.36 ± 3.569
eSun Standard Peach Pink + EtOH wash + 20 mins cured	0.93 ± 0.014	-	-
Prusament Tough Orange 10 mins cured	0.96 ± 0.014	80.76 ± 0.468	44.92 ± 7.965
Prusament Tough Orange 20 mins cured	0.99 ± 0.010	81.86 ± 0.436	54.84 ± 0.757
Prusament Tough Orange 30 mins cured	1.01 ± 0.010	82.91 ± 0.557	31.551 ± 7.856
Prusament Tough Orange + EtOH wash + 20 mins cured	0.99 ± 0.002	-	-
Liqcreate BioMed Clear 10 mins cured	0.93 ± 0.034	85.86 ± 0.688	71.288 ± 10.212
Liqcreate BioMed Clear 20 mins cured	0.96 ± 0.037	86.47 ± 0.364	80.633 ± 22.378
Liqcreate BioMed Clear 30 mins cured	1.00 ± 0.016	86.42 ± 0.342	95.723 ± 4.923
Liqcreate BioMed Clear + EtOH wash + 20 mins cured	0.96 ± 0.049	-	-

17 General info on HPLC analysis

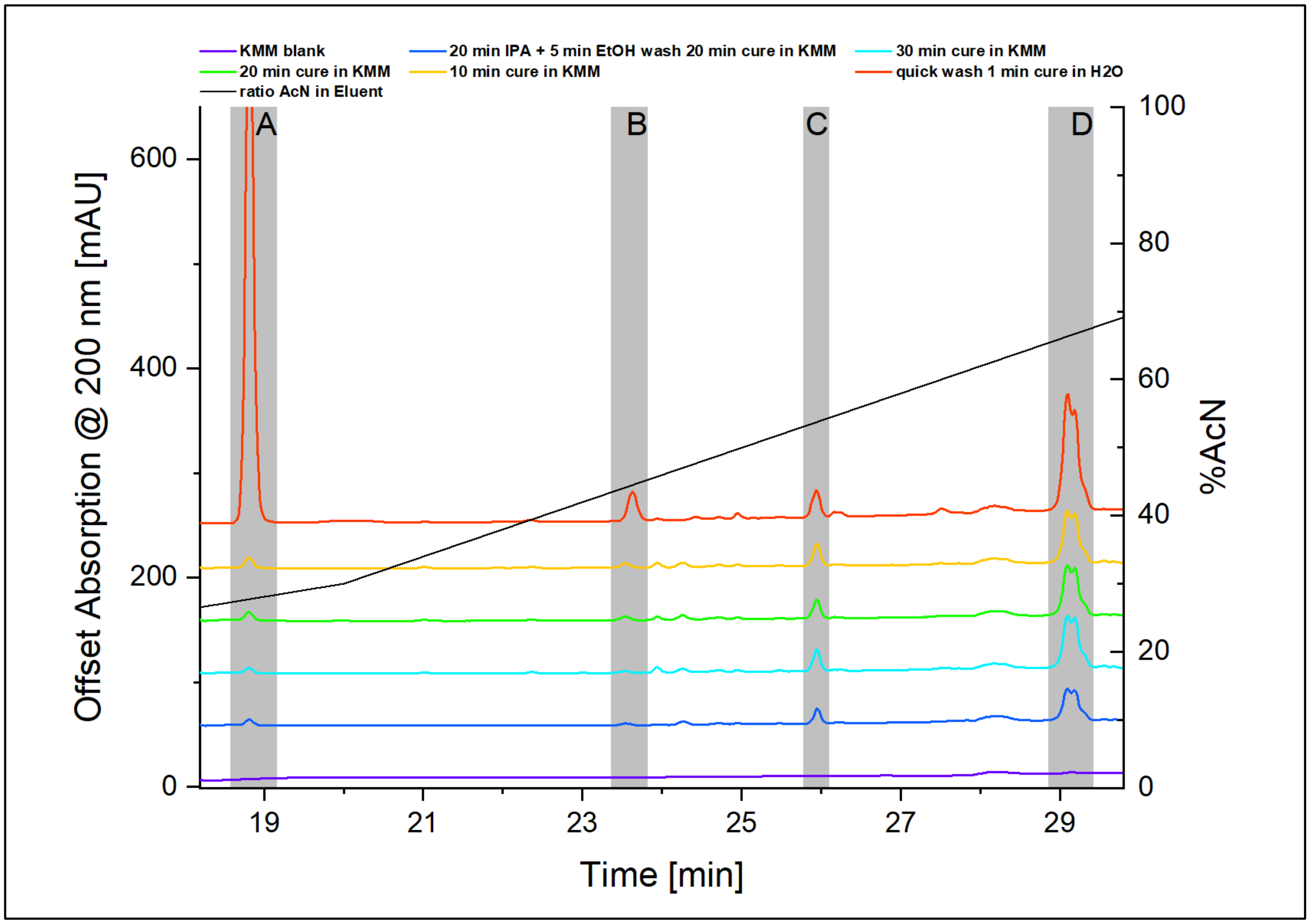

Supplementary Figure 31 Stacked Chromatograms (Offset: 75 mAU) of the KMM blank and leachates of five Liqcreate Biomed Clear samples with different post processing regimes. Referenced Peak Regions (A to D) marked in gray and liquid phase given as Acetonitrile ratio (black). Column: Luna® 5 µm C18(2) 100 Å (250 x 4,6 mm; Phenomenex, XX), Gradient: H2O/ Acetonitrile, 1 mL/min; 95:5 (0 – 7 min) → 70:30 (20 min) → 30:70 (30 min) → 0:100 (40 – 47 min) → 95:5 (57 – 70 min); detection wavelength: 200 nm / reference wavelength: 360 nm.

Stacked Chromatograms (Offset: 75) of the KMM blank and leachates of five Liqcreate Biomed Clear samples with different post processing regimes. Referenced Peak Regions (A to D) marked in gray and liquid phase given as Acetonitrile ratio (black). Column: Luna® 5 µm C18(2) 100 Å (250 x 4,6 mm; Phenomenex, Inc., US), Gradient: H2O/ Acetonitrile, 1 mL/min; 95:5 (0 – 7 min) → 70:30 (20 min) → 30:70 (30 min) → 0:100 (40 – 47 min) → 95:5 (57 – 70 min); detection wavelength: 200 nm/ reference wavelength: 360 nm.

Method description (for SI)
All liquid samples tested were previously filtered through 0.22 µm regenerated cellulose filters (VWR, DE) and transferred to 1,5 mL HPLC vials. Elution was performed with a gradient of deionized water additionally filtered (0.2 µm PES filter, VWR, DE) and Acetonitrile (HPLC grade, Knauer, DE). Samples were analyzed on an AZURA® 862 bar HPLC system (Knauer, DE), equipped with a P 6.1

L pump (LPG), CT 2.1 column oven (set to 20°C), and UV/Vis detector (MWD 2.1 L; at 200 nm). The injection volume was 20 µL.

Outcomes:
In the HPLC analysis we characterized four peak regions of interest, of which the final one consists of two peaks. For comparison tests were conducted on leachate samples of the four different post processing protocols (20 min IPA wash + 5 min EtOH wash with 20 min curing; 10 min IPA wash each and 30 min curing, 20 min curing or 10 min curing) on Liqcreate Biomed Clear. Additionally, a KMM blank sample was measured, as well as a additional leachate sample which was prepared to act as a worst-case scenario. For this extract three similar test disks were printed, but the post processing was reduced to a quick manual wash with IPA (approx. 1 min) and two curing steps (1 min each) to create sample disks with non-sticky surfaces on each side. These sample disks were sterilized in the glass vessel, before the extraction medium was added and leached similar to the other samples for 140 ± 2 hours at 37°C. To further increase the extraction, effect the leaching was done with 13.1 mL deionized water instead of KMM.
The peak area of component A (retention time: 18,82 min) is continuously increasing for samples with shorter curing times and shows a drastic rise for the samples which were under processed on purpose. In comparison of the two samples with 20 min curing time component A was less present in the sample intensified washing step. The Peaks of component B (retention time: 23,56 min) shows a similar trend, although the effect of the increased washing is proportionally higher. Component C (retention time: 25,95 min) shows an increased area for the quick washed sample and a decreased area for the sample with the intensified washing regime. The samples with total curing times of 10 to 30 min show similar peak areas in comparison. Finally, component D (retention time: 29,10 min) appears as a split peak and shows a similar trend as the previous component C.
In total the chromatograms show a general trend, that additional washing and curing reduces the total amount of components extracted by leaching, as the sample with drastically decreased post processing exhibits bigger peak areas for all of the four characterized components. Additionally, it was found that the more intense washing step, with longer exposure to IPA and an added step in EtOH leads to lower peak areas in direct comparison to the second sample cured for 20 minutes in total. For components A and B, the washing seems to have a more minute effect than an increase in curing time. Components C and D show an inverse effect comparing longer curing and increased washing intensity. For the latter two components clear trends with changing the curing time could be found.
